# A global scoping review of community-based mentoring interventions for adolescent girls during pregnancy and after birth

**DOI:** 10.7189/jogh.16.04135

**Published:** 2026-06-02

**Authors:** Cristina Fernandez Turienzo, Mangenda Kamara, Lucy November, Philemon Kamara, Kaat De Backer, Hannah Rayment-Jones, Appiah M Kingsford, Prince T Williams, Jane Sandall

**Affiliations:** 1 Department of Women & Children’s Health, Faculty of Life Sciences & Medicine, King’s College London, London, UK; 2 University of Sierra Leone, Freetown, Sierra Leone; 3 Lifeline Nehemiah Projects, Freetown, Sierra Leone; 4 Welbodi Partnership, Freetown, Sierra Leone

## Abstract

**Background:**

Integrated, evidence-based interventions across health, education, and social systems are needed to support child and adolescent development, especially for pregnant adolescents. While youth mentoring interventions show promise in improving health and well-being outcomes, existing studies largely exclude this population group. To address this gap, we conducted a global scoping review of community-based mentoring interventions for adolescent girls during pregnancy and after birth.

**Methods:**

We performed a scoping review using the Joanna Briggs Institute scoping review methodology. First, we searched (Embase, MEDLINE, CENTRAL, CINAHL, PsycINFO, Web of Science, IBSS, and Global Health), grey literature, and international registers from inception until July 2025 (no language, setting, or time limitations) for studies that reported on a mentoring intervention for adolescent girls (aged 10–19 years old) during pregnancy and after birth that were delivered in any community settings by local members. To be included, the studies had to report on the intervention theory, methods, and components; any health, social, educational outcomes; and key challenges and lessons learnt. Following screening, we extracted and narratively synthesized the data from the included studies.

**Results:**

We screened 1085 titles and abstracts and 97 full text records and included 13 studies (25 records) on community-based mentoring interventions for adolescent girls during pregnancy and after birth. These studies came mostly from the USA, with one delivered in Malawi and Sierra Leone each. They were conducted between 1991 and 2024 and used varied designs, including randomised trials or qualitative and mixed-methods research. Interventions were home-, school-, or community-based and aimed to reduce mortality and improve maternal, perinatal and infant health; reduce repeat pregnancies; support education and employment; and enhance well-being. Mentors, often local volunteers, provided emotional support, practical guidance, and links to services. The included studies reported on improved health practices and outcomes, reduced rapid repeat pregnancies, increased school engagement, and greater self-esteem and well-being. Key challenges were recruitment and retention, role clarity, limited resources, and structural barriers like poverty and stigma. Strong, culturally sensitive mentor-mentee relationships and integration with existing services were critical for success.

**Conclusions:**

Community-based mentoring interventions for adolescent girls during pregnancy and after birth were reported to provide crucial support in improving health and enhancing educational attainment, economic stability, and overall psychosocial well-being, especially when relationships were sustained and culturally sensitive. The strength of evidence, however, varied across different outcomes and contexts. Further research is needed to fully understand efficacy, generalisability and mechanisms, particularly for specific outcomes and adolescent populations in low- and middle-income countries.

The latest Lancet series on optimising child and adolescent health and development highlights the need to support a holistic agenda to improve the integration and implementation of evidence-based interventions across health, education, and social systems to protect, nurture, and support the health and developmental potential of every child and adolescent [1]. In addition to health systems, social systems such as communities, families, and schools are crucial for promoting health and shaping the trajectories of young people’s lives, including those of pregnant and parenting adolescents [2]. These groups face a unique convergence of challenges that distinguish them from both non-pregnant peers and adult mothers, including heightened social stigma, disrupted education, limited access to age-appropriate health and social services, and increased vulnerability to poor physical and mental health outcomes [3]. The perinatal period presents a crucial window for intervention in this context, not only to support maternal health and perinatal outcomes, but also to address adolescents’ psychosocial development, educational continuity, and long-term well-being. Evidence from recent global research underscores the need for tailored, developmentally appropriate, and context-sensitive approaches that respond to the specific needs of adolescent mothers and their children [4]. Mentoring, particularly when embedded in community-based systems, may offer a promising strategy to provide this sustained, relational, and empowering support [5].

A previous Cochrane review found that community-based interventions improved maternal and neonatal health outcomes, particularly in low-income and middle-income countries (LMICs) [6]. However, it did not include mentoring interventions and it was unclear how many trials included adolescents. Meta-analyses of youth mentoring programmes, including community-based ones, have found that they generally improve outcomes across academic, behavioural, emotional, and social areas of young people’s lives [5,7]. The evidence from these studies suggests that longer mentoring relationships are linked to better outcomes [5,7]. The role of the matching process, training, and motivation, and the need for goal-oriented programmes are keys to mentoring success [7]. Goldner and Ben-Eliyahu [8] tried to unpack the relational processes in community-based youth mentoring that promote high relationship quality and generate the most significant benefits. They found that sufficiently long, supportive, reliable, trustworthy, and balanced mentoring relationships serve as building blocks in promoting mentees’ development and minimising adversity, and consideration needs to be given to mechanisms of change and moderating factors, *i.e.* to both mentors’ and mentees’ characteristics, as well as to how they are matched [8]. In addition, most mentoring studies were conducted in North America and Europe, rarely included pregnant adolescents, and provided insufficient data on health and longer-term well-being effects [5,7]. To address these gaps, we conducted a global scoping review of community-based mentoring interventions aimed at improving the health and development of pregnant and parenting adolescent girls.

## METHODS

We selected the scoping review as the most appropriate methodology for identifying and summarising different types of evidence on community-based mentoring interventions in pregnant, birthing, and postnatal adolescent girls to identify research gaps and formulate recommendations for the future research [9]. We ran a preliminary search in PROSPERO, PubMed, and the Cochrane Database of Systematic Reviews and found no such existing or in-progress review. The Joanna Briggs Institute (JBI) scoping review methodology guided this review through the following steps [10]: identifying the research question, developing a search strategy, selecting studies, and analysing/presenting. We report our findings per the PRISMA-ScR guidelines [11].

Our review question was: what evidence supports the use of community-based mentoring interventions for adolescent girls during pregnancy and after birth?

### Search strategy

We developed the search strategy and inclusion criteria using the population, concept, and context (PCC) framework recommended for scoping reviews [12], as follows:

– population: adolescent girls during the antenatal, intrapartum, or postnatal period;

– concept: community-based mentoring interventions;

– context: any country, any community setting.

We searched Embase, MEDLINE, CENTRAL, CINAHL, PsycINFO, Web of Science, IBSS, and Global Health for published studies up to 1 July 2025, with no language, setting, or time limitations. We also hand-searched the bibliographies and reference lists of identified studies and other reviews; reviewed relevant websites, national and international reports and guidelines, and dissertations and theses; and performed searches in Google Scholar, the ISRCTN registry, and the Australian and New Zealand Clinical Trials Registry. Search terms, keywords and strategies (Text S1 in the **Online Supplementary Document**).

### Inclusion and exclusion criteria

We included studies of community-based mentoring interventions targeting adolescent girls (ages 10–19, per World Health Organization definition [13]) during pregnancy and/or up to one year postpartum and aimed at improving their health and well-being. Community-based mentoring was defined primarily by its relational, supportive foundation rather than purely clinical or case-management objectives.

We included studies of interventions that included community-based mentoring irrespective of whether they included other elements (*e.g.* education, income generation support). Mentoring had to occur in community settings (*e.g.* homes, schools, health centres) and be delivered by more experienced individuals who are community members. Community members could include both volunteers and community-embedded leaders (*e.g.* church leaders, activists, school officials, business leaders, woman’s leaders, community chiefs). Mentors could provide one-on-one or group-based support, as long as the intervention was relationship-based (in-person and/or *via* mobile phone). Studies could include or omit comparison groups (*e.g.* usual care or alternative interventions) and be conducted in any setting or country. They had to report on at least one of the following: intervention theory; study population; design; challenges; setting; organisational context; intervention components; effect on health outcomes; engagement in education, social, or economic indicators; and key challenges and lessons learnt.

We excluded studies of interventions that did not include a relational community-based mentoring component; studies that included mixed age samples (*e.g.* ages <22 or <24), but did not have separable adolescent data; or studies where the sample did not meet World Health Organization age threshold.

### Study selection

We uploaded all records to Covidence (Covidence, Melbourne, Australia) and removed any duplicates. Study selection occurred in two stages. Title/abstract screening was conducted by one experienced author (CFT) for efficiency, while full-text review and data extraction were conducted independently by two authors to ensure reliability (CFT, LN, MK, AOH, KDB, HRJ). To mitigate risk of missing studies in initial screening, we employed a highly conservative approach, where any record that was potentially relevant or lacked sufficient detail for exclusion was automatically progressed. Two authors independently extracted the study data and assessed quality using the Mixed Methods Appraisal Tool (MMAT) [14], which was used descriptively to provide an overview of the evidence base and identify common methodological limitations across the included studies, rather than to exclude studies or numerically weight the synthesis of findings.

Discrepancies were resolved through discussion or by consulting a third author. We did not exclude studies based on quality, though we considered this in the synthesis.

### Data extraction and analysis

We designed a data extraction form and piloted it on a small sample of papers, after which two authors applied it to all studies (CFT, LN, MK, APH, KDB, HRJ). A third author was available to discuss any discrepancies or disagreements if necessary. The data extraction form included a description of the study’s details (*i.e.* name, institution, year, country, setting), design and methodology, participants, intervention procedures and comparator (if applicable), underpinning theory of change, outcomes, and a blank field for reviewer’s notes (Text S2 in the **Online Supplementary Document**). When information regarding any of the above was unclear, we contacted the authors of the original studies to provide further details.

We synthesised and summarised the data narratively and tabulated them to examine patterns according to (where appropriate) context, patient characteristics, intervention components, outcomes, size of treatment effect, and engagement in social, educational, and economic activities. Our aim was to explore current evidence and identify gaps and primary research needed.

## RESULTS

**Figure 1:**
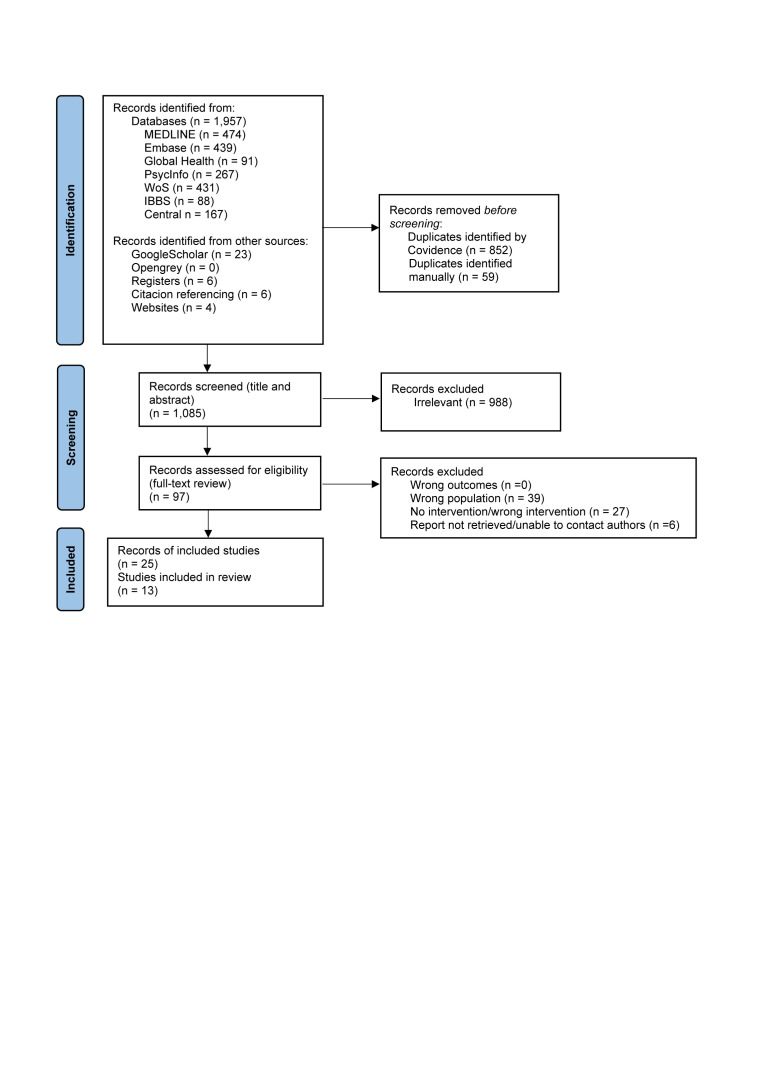
PRISMA flow diagram for study selection.

The searches produced 1996 records, of which 911 were removed as duplicates, leaving 1085 for title and abstracts screening. After this stage, 97 records remained for full-text review, where 72 were excluded due to covering the wrong population (*e.g.* adult women, adolescent boys and girls, adolescent girls not pregnant/postpartum, mixed sample of <22, <24, or <25 years of age; adolescents’ parents), having no or a wrong intervention (*e.g.* adolescent pregnancy programmes, peer educator support or community-based care, but no mentoring), or not having their full text available, or because we were unable to contact the study authors. We finally included 25 records representing 13 studies [15–27] (**Figure 1**, **Table 1**).

**Table 1:** Characteristics of included studies

Study (year)	Country	Design	Aim	Population	Interventions	Theory of change	Outcomes
Black *et al*. (2006) [15]	USA	Quantitative (RCT)	To examine whether a home-based mentoring intervention was effective in preventing second births within two years of first birth	181 participants. First time mothers <18 years at delivery, Black race, no chronic illness, or drug use; and infants >37 weeks and >2500 g with no congenital problems, chronic illnesses, or disabilities. Black: 43%; Hispanic: 45%; White; Other: 2%.	Postnatal: a home-based intervention curriculum, based on social cognitive theory, and focused on interpersonal negotiation skills, adolescent development, and parenting. Mentors: college-educated, black, single mothers, ‘big sister’. Duration: one year. Control: similar in terms of age, education, location, ethnicity, and risk factors. After baseline, they received no further contact until evaluation visits.	Grounded in social cognitive theory, emphasising cultural norms, modelling, self-efficacy, and social support. It enhanced self-efficacy through role playing and goal setting, and fostered support by involving family, conducting sessions at home, and using mentorship.	Risk behaviours and psychosocial factors at baseline and 24 months. Both groups (adolescent mothers with and without a second baby) showed declines in risk behaviours like fighting and arrests over time. At baseline, second-baby mothers were slightly older, more likely to have dropped out of school, and reported higher parenting efficacy. By 24 months, they were more likely to be married or living with a partner, had higher self-esteem and reported positive life events, but not more likely to have advanced in education.
Anne Bogat *et al*. (2008) [16]	USA	Qualitative (as part of a modified RCT)	To describe experiences and stages of mentor-mentee relationships in a programme for pregnant and parenting adolescents	175 pregnant/parenting girls enrolled at special school during a four-year period, 68 randomly assigned to experimental group, and 51 received mentors: 53% African-American; 18% European-American; 12% Latino; 5% Native American; 2% Asian American.	Antenatal/postnatal: Local female parents recruited through community outreach. Mentors completed a four-hour training seminar and committed to a nine-month relationship, spending at least two hours per week – one with their mentee and one in mentor support meetings. Control: Not described.	Background information on theory of mentoring interventions for youth and pregnant/parenting girls, but not specifically articulated for the study.	Relationship progressed through three stages, each with distinct challenges. Early on, mentors struggled to build connections as some mentees withdrew, often due to fear of intimacy or rejection, leading to disengagement from both parties. In the middle stage, relationships were strained by conflict, especially when mentees became overly dependent, and mentors felt overwhelmed or unable to set boundaries. Toward the end, difficulties discussing termination led to reduced contact, limiting opportunities to transition into a more informal, ongoing relationship after the programme ended.
Carbone *et al*. (2019) [17]	Malawi	Qualitative (focus group discussions)	To explore the adaptation of Mothers2Mothers Mentor Mother Model for adolescent mothers living with HIV in Malawi	Black African HIV-infected adolescent mothers ages 15–19 years in four districts. Two groups: those who had experience with m2m programming (8 FGDs, n = 38); those who did not (8 FGDs, n = 34)	Postnatal: Mothers2Mothers (m2m) Mentor Mother Model: engages women living with HIV who have completed the PMTCT process to support other HIV-positive mothers. The model, implemented in community and healthcare facilities, is being adapted to better address the unique needs of adolescent mothers and improve care engagement and health outcomes in this group.	Tailoring the programme to meet adolescent mothers’ specific needs would improve their engagement with HIV care, and do increase retention, and reduce the risk of HIV transmission to their infants.	Numerous barriers to PMTCT care; preference for peer-driven support services; with relatable, non-judgmental young women who are also mothers living with HIV, ensuring privacy and addressing stigma, poverty, and food insecurity. M2m programs to include socioeconomic aspects *e.g.* cash transfers/income-generating activities, to reduce structural barriers. To effectively engage/retain adolescent mothers in the PMTCT cascade, programs should empower them to meet basic needs, build livelihoods, and access continuous, peer-led support.
Estrada (1997) [18]	USA	Mixed methods	To evaluate the impact of a mentoring program's accountability, utilisation, and entrenchment	Teenagers: less-than/equal to 18 years must be pregnant and have referral tickets from two local hospitals or three district schools, or self-referrals (n=14); steering committee members (n = 10); coordinator (n = 1); and mentors (n = 9). (34 participants in total). White 4 (36%); Mexican American: 6 (54%); Other: 1(9%)	Antenatal/postnatal: The mentoring programme pairs trained volunteer mentors, many with lived experience, from the local community with pregnant/ parenting adolescents for up to 1one year, offering support, advocacy, guidance while connecting them to community services. Recruitment occurs through local hospitals, schools, and agencies. Mentoring in the community. Mentors are supported *via* a buddy system with steering committee members and attend bi-annual gatherings for connection and recognition.	The mentoring programme would help prevent repeat future pregnancies, support healthy infant outcomes, and promote school retention. Mentors were expected to serve as positive role models and provide guidance to girls lacking supportive relationships; offer important info on health topics and improve access to key community resources.	At steering committee level: the programme met initial goals by recruiting/training mentors and establishing foundational structures, with strong commitment from committee members and community. Challenges: difficulty in recruiting mentors, lack of full-time coordination, limited volunteer time, and unclear accountability systems. Mentors joined out of a desire to support girls, with most forming positive relationships and finding the experience meaningful, despite facing unclear role expectations, limited guidance, and resource access issues. Suggestions included improving training, enhancing comms with committee, and organising more group activities. Mentees reported overwhelmingly positive experiences, describing mentors as supportive guides who helped them access educational and social services, build confidence, and feel heard. They suggested: more social opportunities, job training support, and services addressing mental health and childcare needs.
Flynn (1999) [19]	USA	Quantitative (before and after)	To analyse the effectiveness of a programme aimed at improving infant outcomes by enhancing health practices and parenting skills among low-income, urban pregnant and parenting adolescents at risk for child maltreatment	137 adolescents; high-risk, girls who were: aged ≤18; Medicaid eligible; residents in the area; not clients of the Division of Youth and Family Services; either pregnant with their first child or within six weeks postpartum; and who screened at risk for potential child maltreatment using the Family Stress Checklist. African American: 71%; Hispanic: 27%; other: 2%.	Antenatal/postnatal: Adolescent Parenting Program of the Essex Valley Visiting Nurse Association: designed to enhance the parenting skills and reduce high-risk behaviours of adolescent mothers. Based on Healthy Families America model of intensive home visitation for three years ‘big sister’ model. The programme served girls and their infants, with services provided by trained paraprofessional family support workers from the local community (for mentoring, goal setting and navigating system) and community health nurses (health guideline). Initially weekly visits for parenting education, health practices, and emotional support, then decreasing through four visits as goals were met. Referrals were primarily made by local clinics, self-referrals considered.	Home visitation programs using paraprofessional mentors are based on mentorship theories, *e.g.* Vance's model, where experienced mentors provide emotional support, guidance, and education, helping mentees become independent. Mentorship is relational, intensive, and aims to guide the mentee toward positive outcomes. Social support theory, *e.g.* Weiss's concept of guidance, aligns with mentorship by emphasising importance of advice and information in improving health behaviours. Research supports that mentorship and social support positively influence maternal health practices, reducing infant mortality and improving outcomes for both mothers and infants.	Findings were compared to local or national data. Mean length of gestation: 39.27 weeks (SD = 41.55). Low birthweight: programme: 4.6%; local and national: 13.5% and 9.42%. Infant mortality rate: program: 0 local and national: 15.8 per 1000. Child neglect cases: programme: 4 (2.91%) local and national: 11%. Only 74 participants completed the Child Abuse Potential Inventory at time 1. Only 19 of the participants completed the CAP at both time 1 and time 2 and had valid scores.
Havens *et al*. (1997) [20]	USA	Quantitative (randomised trial)	To evaluate a mentoring programme designed to decrease the risk of repeat pregnancy among unmarried primiparous teens	Unmarried teens (aged 12–19 years) and in the third trimester of her first pregnancy. Recruited through an alternative school for pregnant teenagers (46%), hospital's obstetrics clinic (36%) and (16%). 110 teens recruited and randomised: 53 mentor group; 57 control group. White: 5%; Black: ~90%; Hispanic: 2%; Native American 2%.	Antenatal/postnatal: The Mentoring Program for Pregnant and Parenting Teens was a collaborative initiative between a medical school, an urban hospital, and a minority-led social service agency. It paired trained mentors with pregnant teens for one year to offer role modelling, education, and social support. Most mentors were African American working women, some with lived experience, who completed six weeks of training on healthcare, contraception, and community resources. They spent at least 12 hours monthly with mentees, combining social activities with structured support on school, parenting, and goal-setting. Mentors received stipends based on hours and attended quarterly training for continued support. Matches were made formally based on observed interactions. Follow-ups occurred at one, two, and five years. Control: Not described.	The programme assumed that the mentor would serve as a role model, an information/education resource, and a source for social support for the teen and her family.	Number of repeat pregnancies: 0: 17 (34.0%) mentor group; 15 (31.2%) control group; 1: 20 (40.0%) mentor group; 20 (41.7%) control group; 2: 12 (24.0%) mentor group; 11 (22.0%) control group; 4: 1 (2.0%) mentor group; 2 (4.2%) control group; unknown: 3 mentor; 9 control group. School advancement: graduated or advanced 2 grades: 28 (52.8%) mentor group; 27 (47.4) control group. Delayed: 15 (28.3%) mentor group; 14 (24.6%) control group. Dropped out: 5 (9.4%) mentor group; 7 (12.3%) control group. Missing: 5 (9.4%) mentor group; 9 (15.8%) control group. Lower, but no significant differences in the Piers-Harris Self-Concept Scale, the Nowicki-Strickland Locus of Control Scale, and the Beck Depression Inventory to explore low self-esteem or psychological distress: 36% 1 repeat pregnancy; 24% had two or more pregnancies 62% of the pregnancies with livebirths (89); 26% abortion.
Hurd *et al*. (2010) [21]	USA	Mixed methods (informal)	To assess potential natural mentoring long term effects on adolescent mothers’ wellbeing as they transitioned from adolescence into adulthood	93 participants; eligible participants were African American teen mothers (about 17–18 years), pregnant or parenting during their senior year or equivalent, with an eight-grade grade point average ≤3.0 and no disabilities.	Postnatal: Natural mentors, defined as non-parent adults (aged ≥25 years) identified by participants as sources of support, guidance, or inspiration. If a parent or guardian was named, participants were prompted to name another adult. Responses were used to create a binary variable (0 = no mentor, 1 = natural mentor).	Based on resilience theory, which highlights promotive factors like individual strengths, supportive relationships that help youth thrive despite adversity. For adolescent mothers facing high risks, resilience theory emphasises strengths in the individual and environment rather than deficits. Natural mentors act as protective resources buffering stress’s negative effects. Authors hypothesised girls would show less depression and anxiety and a weaker link between stress and mental health symptoms over time, demonstrating mentors’ role in fostering resilience.	Various scales measured depressive and anxiety symptoms, stress, parental support, natural mentor presence, and demographics. Of 93 participants, 57 (61%) reported a natural mentor, mainly female extended family (*e.g.* grandmothers, aunts, cousins) or older siblings; others included godparents, neighbors, ministers, and parents’ friends. Natural mentors moderated the link between stress and both depression and anxiety, suggesting they may support resilience through coping help, emotional support, material aid, or childcare assistance. Participants with natural mentors showed fewer depression and anxiety symptoms over time.
Klaw *et al*. (2003) [22], Klaw *et al*. (1995) [23]	USA	Quantitative (interview administered survey)	To explore academic attainment of pregnant and parenting African American adolescents as they transitioned from pregnancy or recent delivery to two years postpartum [23] and understand the extent to which natural mentors were associated with positive educational and career outcomes [22].	198 [23] and 204 [22] African-American adolescents between the ages of 11 and 19 years who were enrolled in an alternative school for pregnant and parenting student during the academic year 1992–1993.	Antenatal/postnatal: natural mentors, defined as non-parental adults, not peers or partners, who offered guidelines, support, and served as role models. Participants were asked if they had such a person (older, more experienced, and personally invested in them). Key traits included reliability, belief in the participant, inspiration, influence on choices, and modelling of personal and career success. Interviewers ensured participants clearly understood this definition.	Natural mentoring relationships (supportive, trusting connections with older, experienced nonparental adults within a young person’s existing network) are expected to improve outcomes for African American adolescent mothers. These mentors provide emotional, practical, and motivational support during the postpartum period, helping young mothers stay engaged in education and future goals. As a result, adolescent mothers may experience lower depression, greater emotional support, stronger academic and career engagement, and an increased sense of future orientation. The type and strength of mentoring support (*e.g.* emotional *vs*. academic) may shape these effects. Natural mentors act, overall, as a protective factor by fostering resilience and reducing the risk of school dropout.	Over half of participants (57.8%) reported having mentors, most often aunts, grandmothers, or other trusted adults. These relationships were typically long-standing (often over 15 years), frequent, and geographically close, with many expecting them to last a lifetime. Mentor support was associated with greater involvement in career activities, more positive beliefs about future opportunities, and higher optimism, though it did not fully close the gap between aspirations and expectations [22]. Simultaneously, long-term mentoring was strongly linked to improved educational outcomes: after two years, dropout rates were much lower among those with long-term mentors (35% *vs*. 64%). These mentors, often relatives or parental figures, provided consistent emotional, social, and practical support, including help with school, advice, and skill-building. Compared to maternal support, mentoring was rated as more important and satisfying and played a key role in fostering resilience and positive career outlooks over time [23].
	USA	Quantitative (interview administered survey)	To explore the academic attainment of African American adolescents as they made the transition from pregnancy or recent delivery to two years postpartum	198 African-American adolescents between the ages of 11 and 19 years who were enrolled in an alternative school for pregnant and parenting student (unclear academic year and unclear if same sample as Klaw 1995).	Antenatal/postnatal: During interviews, participants were asked if they had a nonparental adult mentor who provided support and guidance. A mentor was defined as an older, more experienced adult (not a peer or romantic partner) who takes a special interest in the participant. To qualify, the mentor had to meet four criteria: being dependable, caring, inspiring, and influential in the participant's life. Interviewers ensured participants clearly understood this definition.	Natural mentors act as a protective factor for African American adolescent mothers by supporting their educational success. These nonparental adults, often relatives, provide emotional, practical, and motivational support during the difficult postpartum period. By modelling positive behaviours, reinforcing the value of education, and offering guidance and assistance, mentors help young mothers stay engaged in school and build resilience, reducing their risk of dropout.	Differences in educational attainment function as relationship duration: after two years, 35% with long-term mentors dropped out of school versus 64% with no mentors. Long-term mentoring cut dropout odds by over three times, while maternal support had no significant effect. Characteristics of long-term mentoring: long-term mentors were often relatives and seen as parental figures or older siblings. These relationships were strong, with weekly or daily contact, lasting about 14 years on average and expected to last lifelong. Mentors supported by helping with school, giving advice, lending items, teaching job skills, and providing fun opportunities. Long-term mentor versus maternal support: maternal support levels stayed the same, but satisfaction was higher (in the terminated mentor group). Over time, both mentors and mothers gave less support, but mentors provided more emotional, social, and guidance support. Participants rated mentor support as more important and satisfying at both times.
Lin *et al*. (2019) [24]	USA	Quantitative (interview administered survey + open ended questions)	To examine educational attainment, employment, and repeated pregnancies of adolescent mothers 1–5 years post mentoring programme	74 teen mothers of a single child, under 20 years old and not currently pregnant. >90% were African American/biracial.	Postnatal: The Maikuru Program: ‘Maikurus’, at least 15 years older and screened for suitability, were recruited through schools, clinics, and community outreach, with matches based on shared interests. Maikurus completed a one-day training, then attended eight weekly group sessions with their mentees, followed by quarterly meetings. Sessions, led by professional facilitators, covered topics like contraception, finances, relationships, parenting. Mentors provided guidance and emotional support, with at least monthly contact encouraged. Community settings. Ongoing mentor support was provided through regular peer meetings. After the formal program, continued contact was promoted through social media and optional monthly gatherings. Outcomes were assessed at 6, 12, 18, and 24 months.	The programme was designed to increase girls’ self-esteem within a supportive, non-judgmental environment. Its core mechanisms involved providing a comprehensive mix of messages, relationships, and services: linking mentees to existing educational and health services, supplemented by workshops on parenting, employability, and life management, as well as group and individual counselling. Mentes were paired with adult ‘community women’ who offered psychological and practical support. The programme theorised that this holistic approach, including comprehensive brokered services, supportive mentor relationships, and individualised planning, would lead to improved parenting skills, better child development, and greater self-sufficiency	Educational attainment at 1–5 years follow-up: attending high school: 5 (26.3%); high school graduate/GED completed: 5 (26.3%); some post high school education: 8 (42.1%); college graduate: 1 (5.3%). Employed at follow-up: 12 (63.2%). No subsequent pregnancies: 10 (52.6%). <20 years old subsequent pregnancy: 2 (10.5%). ≥20 years old subsequent pregnancy: 5 (26.3%). Unknown age subsequent pregnancy: 2 (10.5%). Satisfaction: participants shared mostly positive feedback about the benefits: appreciation for parenting support, guidance, and resources. One teen said it helped her become a better mom and inspired her to open an orphanage. Others felt it helped them stay focused and feel less alone as young mothers. Of the 74 teen mothers, 23 dropped out, 51 completed the programme, 19 reached for follow up
Fernandez Turienzo *et al*. (2025) [25]	Sierra Leone	Cluster RCT	To evaluate the feasibility and potential effects of a community-based mentoring programme from pregnancy up to one year after birth	673 pregnant adolescent girls younger than 18 years living in those cluster communities and presenting for maternity care were eligible (six clusters with 372 girls in intervention; six clusters with 301 girls in control group)	Antenatal/postnatal: 2YoungLives four core components: community engagement and involvement; recruitment, training, and supervision of mentors; mentor-mentee matching; and mentoring and additional activities. 2YoungLives delivered alongside standard maternity care, provides mentoring and supports pregnant adolescents for up to one year after birth. Volunteer mentors, chosen for their community reputation, each support three girls through weekly one-on-one sessions. Working in teams with a coordinator, mentors also attend monthly group gatherings with mentees, including shared meals and guest speakers. The programme offers parenting support, health advocacy, and small business training, with each girl receiving a start-up fund. Mentors receive a stipend and three days of training in safeguarding and maternal health. Girls are recruited informally, and follow-up continues for one year. Matching based on language and geographical proximity. Control: non mentoring + standard maternity care.	It was hypothesised that 2YoungLives could save lives and improve the health and wellbeing of pregnant adolescents and their babies while simultaneously enhancing livelihoods through relationship building, access and engagement with maternity services, advocacy with families and health workers, empowerment (health, social, and economic) and respectful community engagement.	Maternal + perinatal mortality composite: 2YL: 23/361 (6%); control: 35/279 (13%). Maternal death: 2YL: 0/361); control 1/279 (<1%). Stillbirth: 2YL: 12/361 (3%); control: 17/279 (6%). Neonatal death: 2YL: 11/361 (3%); control: 18/279 (6%). Post-abortion: 2YL: 7/358 (2%); control: 4/277 (1%). Vaginal births: 2YL: 316/345 (92%); control: 248/266 (93%). Caesarean sections: 20/345 (6%); control: 9/266 (3%). Preterm birth: 97/332 (29%); control: 83/261 (32%). Births by skilled health professionals: 2YL: 254/346 (73%); control: 109/266 (41%). Place of birth: community: 21/346 (6%) ; control: 26/267 (10%); peripheral health unit: 2YoungLives: 239/346 (69%); control: 192/267 (72%); or hospital): 86/346 (25%); control: 49/267 (18%). Complications: 2YL >50%; <50% control. Apgar score greater than 7 at 5 min: 11/295 (4%); control: 8/224 (4%). Mean birthweight: 2895.90 (SD = 468.37); control: 3048.34 (SD = 386.23). Resuscitation: 119/317 (37%); control: 71/242 (29%). Immediate breastfeeding: 307/316 (97%) control: 236/243 (97%). Kangaroo mother care: 248/318 (78%); control: 203/247 (83%). Admission to neonatal intensive care unit: 6/320 (2%); control: 4/243 (2%). Other processes reported in Appendix 2 in the **Online Supplementary Document**.
Quint *et al*. (1991) [26]	USA	Mixed methods	To improve employment, welfare, and child-related outcomes among disadvantaged school-age mothers (aged ≤17 years)	805 girls enrolled; ethnically mixed group of pregnant girls and mothers who were 17 or younger, lacked a high school diploma, and were receiving or eligible for AFDC. Sites initially drew almost all their participants from hospitals, health clinics, welfare offices, schools, and other agencies serving young mothers. Black: 43%; Hispanic: 45%; White/other: 2%	Antenatal/postnatal: Project Redirection: 18-month intervention for pregnant and parenting teens combining practical services, emotional support, and mentorship. The programme emphasised health care, education, employment preparation, and personal development through workshops, individual and group counselling, and individualised participant plans. Its most distinctive feature was the use of community women as mentors, each supporting 1–5 teens with at least five hours per week of personalised assistance, including escorting them to appointments, offering emotional support, and reinforcing programme goals. Mentors recruited from local communities, often with lived experience, and received training and supervision. The intervention focused on fostering self-esteem in a non-judgmental environment as a foundation for service use and long-term self-sufficiency. Services were coordinated through a brokerage model, linking teens to existing community resources. Control: a comparison group from similar cities receiving usual care was used to assess outcomes (follow-up at one, two, and five years).	It was hypothesised that providing adolescent mothers with tailored services, supportive relationships with community women mentors, individualised participant plans, and peer group sessions lead to increased education continuation, improved employment skills, delayed subsequent pregnancies, and greater personal and economic self-sufficiency. These supports were expected to have immediate benefits for both mothers and their children and foster lasting positive changes in attitudes and behaviours aligned with programme goals.	Health/service use: most participants attended scheduled maternal and infant health visits. Participation in life management activities (*e.g.* parenting, family planning) was high, though attendance varied. Education: School enrollment was higher in the Redirection group at one and two years, but by year five, both groups had similar high school/ GED completion rates (48%). Employment/income: Redirection participants had slightly higher employment rates and worked more hours by year five, earning more weekly. Overall household income was similar. Welfare dependency: At five years, fewer Redirection participants were on welfare (49% *vs*. 59%). Fertility/contraception: programme teens had fewer pregnancies and abortions by year five, but overall small differences. Contraceptive use at last intercourse was similar. Parenting & child development: redirection mothers had better home environments, were more likely to breastfeed, and enrolled their children in Head Start more often. Their children showed fewer behavior problems and had slightly better vocabulary scores at five years.
Waller *et al*. (1999) [27]	USA	Other (comparative report)	To contrast historical and psychosocial experiences of parenting teens with common myths and to develop guidelines for implementing a community mentoring programme	45 volunteer mentors; 50 teens. The only stipulation is that volunteer mentors need to agree to keep appointments with their mentors. Includes girls who fall into a moderate risk category (*i.e.* conditions such as social isolation, childhood abuse, lack of knowledge/experience related to parenting, previous child protective services, multiple life stresses). Those with more serious issues (*e.g.* mental health, substance misuse) are referred elsewhere.	Antenatal/postnatal: community-based mentoring programme for pregnant and parenting teens, cantered on a seven-week volunteer mentor training (topics: prenatal care, parenting, coping skills, family violence, community resources) using interactive methods like role play, discussion, presentations. A key goal is fostering empathy and understanding teens’ experiences. Mentors are recruited *via* community outreach/screened for suitability. After training, they commit to weekly one-on-one meetings for one year, supported by monthly group supervision and guidance from a programme coordinator. Mentors serve as educators, role models, and advocates, helping teens navigate parenting, access services, and build self-esteem. Teens volunteer for the programme and matching is based on relationships developed during training. The programme emphasises trust, support, empowerment through emotional and practical guidance.	Providing consistent social support through mentoring relationships mitigates many of the biopsychosocial risks associated with early pregnancy and parenting. By fostering trust and connection, mentors can promote healthier behaviours during pregnancy, reduce stress through emotional support, and encourage continued education and skill-building. These supports are hypothesised to lead to improved maternal and child outcome, such as better birth health, reduced child maltreatment, and increased self-sufficiency, by addressing social isolation and strengthening young mothers’ capacity to adapt and thrive.	Authors suggest two strategies: crucial to evaluate the effectiveness of the mentoring programme in terms of impact on pregnancy/birth outcomes, as well as abuse and neglect reports during the infants’ one year life; but also evaluate the effectiveness of the mentoring programme in terms of the impact on the teen parents: social support (*i.e.* Perceived Social Support Scale by Procidano & Heller, 1983) administered before a mentor has been assigned, and again one year later. Consistent findings indicate that perceived support, defined as the perception that social support is or would be available if it were needed, is the best indicator for the overall construct of social support.

AFDC – Aid for Families with Dependent Children, CAP – Child Abuse Potential, FGD – focused group discussion, m2m – Mothers2Mothers, PMTCT – prevention of mother to child transmission, RCT – randomised controlled trial

The included studies were conducted in USA (n = 11) [15,16,18–24,26,27] , Malawi (n = 1) [17] , and Sierra Leone (n = 1) [25] among more than 2600 participants between 1991 and 2024, with four published before 2005 [19,20,22,27] and four before 1998 [18,23,26,27]. Several used studies quantitative approaches, including randomised trials [15,20,25], before-and-after interventions [19], and surveys, some of which were longitudinal [22-24]. Others used qualitative methods [16,17] or mixed-methods designs, such as exploratory evaluations [21] or quasi-experimental studies with ethnography and interviews [26]. One study focused on developing implementation guidelines based on an existing programme [27].

The primary aims of the 13 studies varied and included: preventing rapid repeat pregnancies [15,20]; reducing mortality and improving maternal, perinatal, and infant health outcomes [19,25]; enhancing education, employment, and well-being [21–24,26]; evaluating programme impact on accountability and utilisation [17]; exploring mentor-mentee relationship dynamics [16,27]; and identifying care barriers and adapting mentor models for HIV-infected adolescent mothers [17]. Study durations ranged from one-year evaluations to multi-year follow-ups, with some having unclear timelines [16,19,21–23].

### Study population

Participants were pregnant and/or parenting adolescents, mainly from urban, low-income, and disadvantaged settings. Many studies focused on Black/African American adolescents [20–24] while others included ethnically mixed groups (*e.g.* Black, Hispanic, Caucasian, mixed) [15,16,18,19,26] or Black African participants [17,25]. Most were single mothers, though some studies included married adolescents [17,25]. Inclusion criteria often specified first-time mothers, specific age ranges (*e.g.* under 18 or 19), or risk factors like Medicaid eligibility, child maltreatment, substance use, or sexually transmitted infections. One study focused on HIV-infected adolescent mothers [17]. Sample sizes ranged from small qualitative studies [17] to large interventions enrolling hundreds [26]. Mean ages at delivery or enrolment generally ranged from 15 to 17 years.

### Community-based mentoring interventions

The mentoring programmes in included studies aimed to provide crucial support, guidance, and resources to improve outcomes for both adolescent mothers and their children. The interventions were mainly home-based [15,18], community-based [17,18,21,24–26], or community school-based [16,20,22,23], but were structured in various ways overall, often adapting to the specific needs and contexts of the target populations [17,24,27]. Many studies shared core objectives, such as reducing mortality and improving health outcomes for mothers and infants, preventing rapid second pregnancies, enhancing educational attainment, and fostering self-sufficiency [15,18,19,24–26], and often leveraged the power of social support and mentorship as key mechanisms for positive change [15,19,20,23,27]. They also emphasised the importance of a caring, adult connection to help young mothers navigate complex life challenges [15,16,22,25].

We identified both formal mentoring programmes, structured deliverables with defined recruitment and training, and natural mentoring relationships (spontaneously occurring supportive connections within an adolescent's existing social network, *e.g.* extended family or neighbours). These were analysed as separate categories to distinguish between implementable services and the organic supportive ecosystems they seek to emulate. Only three studies included natural mentors and focused on relationships that emerged organically rather than on formal mentor-mentee pairing [21-23].

#### Mentoring at its core

Mentors served in various capacities, including confidants, educators, advocates, brokers, and role models [15,18–20,25–27]. They provided emotional support, advice, information, practical assistance, and companionship [16,19,22,25,27], and acted as a main link between the young mothers, service providers and educators, guiding them through the health, social, and education systems [18,19,25]. Mentors helped mentees set short- and long-term goals and identify behaviours needed to achieve them, such as school attendance or skills development [19,25]. In some interventions, they specifically reinforced messages around effective parenting and coping strategies for daily life problems [24–26]. The Maikuru programme, for example, encouraged monthly conversations (phone or in-person) and contact *via* e-mail/text, with mentors acting as advisors on childbearing, relationships, school, employment, and navigating adolescence; however, Maikurus were discouraged from lending money or providing transportation to group meetings [24]. The 2YoungLives scheme provided a small business start-up fund and supported mentees to run small businesses, helping them eat well and prepare for birth; mentors assisted with business identification and adaptation based on seasonal supply and demand [25].

#### Health and parenting education

Interventions like Project Redirection [26] and the Adolescent Parenting Program [19] offered workshops on parenting, child development, and perinatal/paediatric care. The 2YoungLives scheme used pictorial maternal and infant health resources in group settings and one-on-one discussions to share health messages [25]. Mentors in various programmes also provided specific guidance on nutrition, stages of labour, infant care, stress management, and family planning [18,19,24,25]. The Three Generation Study used a 15-minute video featuring successful adolescent mothers discussing conflict avoidance and caregiving; although condoms were provided at every contact in this programme, the main focus was on personal values and decision-making, rather than an overt message advising against second pregnancies [15].

#### Educational and vocational support

Some interventions aimed to encourage academic retention and re-entry into education [18,25]. Project Redirection placed considerable emphasis on employability development, including workshops on job applications and on-the-job behaviour [26]. The Maikuru programme encouraged aspirations to higher education or employment [15], while the 2YoungLives programme encouraged girls to return to education or start vocational training (*i.e.* plumbers, electricians, hospitality) with educational bursaries [25].

#### Psychosocial support and autonomy

Creating a warm, supportive, and non-judgmental environment was crucial to increasing self-esteem and encouraging open discussion of problems. The Three Generation Study aimed to help young mothers achieve autonomy and negotiation skills related to childrearing, particularly in their relationship with their own mothers (the infant’s grandmother) [15]. Adolescent mothers in Malawi expressed a strong desire for peer-led, age-appropriate psychosocial support from mentors with whom they could relate personally – ideally someone of a similar age (18–24 years old) [19]. Few programmes also explicitly involved the mentors assisting with interpersonal disputes between the mothers and their family members or partners [15,25].

#### Community Integration and access to services

Interventions frequently connected participants with existing community educational, social or health services. The ‘brokerage model’ used by Project Redirection involved coordinating services already available in the community rather than duplicating them, through referrals or inviting agency representatives to deliver workshops [26]. Beyond simple referrals, meaningful community engagement was defined by specific, high-intensity strategies. The 2YoungLives scheme deliberately built strong relationships with government clinic staff, where they encouraged mentees to register and accompanied them for maternity care if desired. Advocacy with services such as maternity care providers, schools and vocational training institutes was seen as important aspects of the role to support access and integration [25]. The scheme used a three-visit community engagement strategy to build trust and buy-in; stakeholders included traditional leaders, healthcare providers, teachers, and community members who participated in monthly site gatherings focused on peer support, shared meals, and group discussions on health and education. Similarly, mentor mothers in Malawi engaged traditional leaders to reduce stigma and increase service uptake [17]. Mentors and community health nurses often accompanied adolescents to healthcare or community resource appointments, guiding them through the system [19]. Community mentor mothers in Malawi engaged traditional leaders to increase buy-in and support for health services and to reduce stigma and discrimination [17].

#### Mentor recruitment, training, and supervision

Formal mentors were often community or local volunteers (often with a small stipend) and were recruited from the local community based on: their experience, community knowledge, commitment, kindness, and trustworthiness [25]; their shared experiences, such as being teen mothers themselves or having a daughter who was a teen mother [16]; their experience as a person living with HIV who had navigated the prevention of mother-to-child transmission [17]; and college-educated, black, single mothers in their 20s and the ‘big sisters’ model [15,19].

Recruitment methods varied, including public service announcements, advertisements, mailings, word-of-mouth, and referrals from schools and health centres. Few studies required legal clearances or criminal history checks for mentors [16,24]. Training durations varied, from four-hour seminars [16,18] to one or several days [24–26] or even six to seven weeks-long programmes [20,27]. Unlike formal mentoring programmes, natural mentors were typically non-parental supportive adults who were already part of an adolescent's existing social network [21-23]. They were often identified as extended family members, such as grandmothers, aunts, and older siblings, but could also include godparents, parents’ friends, or neighbours. In some studies, individuals acting as mentors adopted a ‘big sister’ role to foster a supportive rather than authoritarian relationship [23]. These mentors were identified by asking adolescents to nominate an adult (excluding parents, stepparents, romantic partners, or peers) who provided support and guidance, believed in them, inspired them, or significantly influenced their choices and actions [21–23]. Unlike formal mentoring programmes, natural mentoring relationships tended to emerge spontaneously from within the youth's natural support system.

Training content covered the needs of pregnant adolescents, basic maternal, newborn, and infant health (*i.e.* recognition of early warning signs, seeking help), communication skills, safeguarding, programme requirements, and specific types of social support [15,16,24,26], as well as child development, stress management, and child abuse prevention [19,25]. Techniques often include discussion, role-playing, scenarios, and audiovisual materials [16,25,27]. Mentors were trained to foster empathy for girls and understand the subjective experiences underlying girls’ behaviours [27]. Ongoing supervision was critical for mentor success, and this often included weekly or monthly meetings with a coordinator or supervisor [16,19,24–28]. Support group meetings for mentors were common, providing peer support and a forum to discuss problems and solutions [16,20,24,26,27]. Some interventions, like the Adolescent Parenting Program, featured weekly sessions between community health nurses and family support workers to review each adolescent's status and care plan [19]. Mentors in the Three Generation Study were given mentees’ cell phone numbers for frequent contact, even outside scheduled home visits, and often assisted with interpersonal disputes [15].

#### Mentee recruitment and matching

Referrals for recruitment of mentees came from various sources, including hospitals, health clinics, welfare offices, schools, and community agencies. Word-of-mouth and self-referrals were also common. Eligibility often included being pregnant, under a certain age (*e.g.* 17, 18, or under 18/19 years), often first-time parent, and/or low socioeconomic background. Matching mentors and mentees was often done by a programme coordinator, and although not always reported, was sometimes based on interviews about hobbies and interests [18,20,24,27], ethnic background similarity between mentor and mentee [15], or geographical proximity and shared language [25]. Early terminations highlighted the importance of ‘goodness of fit’ and mentors’ ability to navigate initial challenges [16].

#### Mentoring duration

The duration of the mentoring varied from nine month to one year commitments for mentors [16,18,27] or until the baby was around one year old [25]. Some programmes, like the Project Redirection had participants stay for an average of 11.6 months, with a maximum limit of 18 months [26]. Longitudinal studies tracked natural mentoring relationships for several years, demonstrating that enduring relationships were often most meaningful [22,23].

#### Control groups

Three studies included a control group to evaluate the intervention efficacy through randomised trials [15,25] and quasi-experimental designs [26]. Control groups were sometimes vaguely described, and appeared to receive usual care or no specific intervention contact beyond baseline assessment. However, a challenge noted in Project Redirection was that control groups sometimes received more services than anticipated due to an increasing nationwide availability of services, making it a ‘conservative test’ of the programme’s incremental effects. Other studies were qualitative or mixed-methods evaluations that described implementation, challenges, and participant experiences, rather than comparing to a control group.

### Outcomes

#### Maternal and infant health

The included studies reported varied impacts on maternal and infant health, with evidence levels ranging from randomised trials to descriptive surveys. One recent trial found a nearly 50% reduction in a composite of maternal and perinatal deaths, mainly driven by a reduction in perinatal deaths and increased births delivered by skilled birth attendants [25]. Three studies reported reductions in rapid repeat pregnancies [15,24,25]. One study found lower subsequent pregnancy rates at one year, though by five years participants had more children than controls, partly due to fewer abortions [26]. In another study, mentoring appears to reduce low birthweight babies (4.6% *vs*. 13.5%; local and regional: 9.42%) and infant mortality rates (0 *vs*. local 15.8 per 1000, almost twice the state average) [19]. Improved health practices were also noted with improved process outcomes like quality of antenatal visits, referrals, and contraception [25] (although contraception use at last intercourse was similar in other study [26]); and increased prenatal/postpartum visits, smoking cessation, and age-appropriate immunisation [19].

#### Education and employment

Findings regarding education and employment were mixed; while some studies reported positive outcomes like increased school enrolment, these often did not translate into long-term completion or economic stability. One study reported increased school enrolment, but this did not lead to higher completion rates at five years when many girls remained unemployed, nearly half relied on welfare, and the average household income was low (USD 737/month), indicating ongoing poverty [26] Long-term mentoring was reported to help girls stay in school [23] and lower dropout rates among mentees [20]. In one USA-based study, 74% graduated from high school, 47% pursued higher education and 63% gained employment [24]. One intervention supported participants in running small businesses during pregnancy and the first postnatal year through start-up funds and business advice [25]. Broader barriers such as poverty, economic disempowerment, and lack of livelihood resources also persisted in another study [17].

#### Adolescent well-being

Some studies observed improvements in psychosocial well-being, with mentored adolescents reporting reduced symptoms of depression and anxiety [21]; increased self-esteem [17,21,25,26]; greater life optimism and positive life events [17,23]; and improved parenting skills [15,19,26]. Mentees widely valued their relationships with mentors, consistently reporting high overall programme satisfaction [24,26]. They perceived mentors as crucial sources of guidance, support, and friendship [18,19,23], offering emotional, social, and practical assistance [16,19,21,22,26,27]. In one study, monthly gatherings with mentors and other mentees were particularly popular, fostering friendships and peer support [25].

It is important to note that many programmes were multi-component in nature, using mentors to facilitate resource-transfers (*e.g.* business start-up funds, bursaries) and service-navigation (health or social). Consequently, it is difficult to isolate whether positive outcomes were driven by the mentoring relationship itself or by tangible material or structural supports.

### Underpinning theory of change

The systematic application of theory varied across the included studies, with half explicitly stating a guiding framework [15,19,21,25–27], through only a few used articulated logic models to link components to specific mechanisms or outcomes. For example, Project Redirection and 2YoungLives used structured frameworks to connect service brokerage and community advocacy to health and vocational outcomes [25,26]. Other studies mentioned broad theories such as social cognitive theory, which focused on cultural norms, support modelling and self-efficacy to address behavioural mechanisms like repeat pregnancy [15], or resilience theory, which emphasised protective factors that promote positive adjustment despite adversity, such as supportive relationships that buffer against negative outcomes like stress, depression, and anxiety [19]. The concept of mentorship as an intensive, emotionally reciprocal, and often terminal relationship provided a core theoretical framework for understanding relationship quality [16,19]. Some programmes relied on the theoretical premise that mentors serve as role models and sources of social capital [19,20,25,27] to foster self-esteem and provide practical guidance [19,22–26] while the ‘wise woman of the village’ concept specifically aimed to bridge gaps in life skills, social support and educational goals [24].

### Quality assessment

The included studies varied in methodological rigour. While randomised trials were rated as being high quality in MMAT terms, as they met criteria for appropriate randomisation and comparable groups, they remained at risk of bias due to lack of blinding and potential confounding in longitudinal follow-ups [15,25]. Robust mixed-methods designs combined quasi-experimental and qualitative data [19,26], while quantitative descriptive studies used appropriate sampling and validated measures [21,23]. One qualitative study featured clear research questions and well-supported focus group findings [17]. However, several studies were limited by low response rates or high attrition, particularly in longitudinal designs, which potentially introduced selection bias [18,22,24]. Additionally, some mixed-methods studies lacked clear integration [18], and one qualitative study relied on mentor/supervisor notes rather than direct mentee accounts, affecting validity [16]. Quality assessment was inapplicable for one implementation guideline [27].

### Key challenges and lessons learned

Many studies highlighted implementation challenges. For mentee engagement and retention, issues included missed appointments [15,20], high mobility [15,18,29], and mentee and mentor disengagement [17]. Some mentees withdrew early, possibly due to fear of intimacy or rejection, while mentors also disengaged, felt overwhelmed, especially after birth, struggled with boundaries [15], and hesitated to discuss sensitive topics like sexuality and contraception despite training [20,26]. Challenges included unclear roles [18,26,27], high mentor turnover due to limited guidance [15,16,20,26], recruitment and/or retention of qualified volunteers and coordinators [18,24–27], securing adequate funding [18,26,27], and poor communication among staff and committees affecting group cohesion [18]. External barriers like poverty, stigma, food insecurity, and lack of transport appeared to reduce effectiveness [17,26]. Gender inequality and partner violence were major issues for some mothers [17] and ‘brokered services’ in Project Redirection often varied in quality and were sometimes hard to access [26]. Few challenges in trial recruitment were also highlighted in one study, such as preference for other clinics that provided more respectful care, over-reporting of age due to stigma and fear of punitive policies for underage pregnancies, or avoiding care at facilities for similar reasons [25].

Lessons learned emphasised that enduring, high-quality, and consistent mentoring relationships are most impactful, especially when mentors provide emotional support, guidance, and practical assistance [19,21–23,27] and when there is meaningful engagement and trusted relationships with community leaders and healthcare providers [25]. Culturally sensitive communication and matching mentors by similar characteristics or experiences (*e.g.* age or HIV status) can significantly enhance relationship effectiveness [14,15,17,20,24]. Moreover, while formal programmes are valuable, fostering environments where natural mentoring relationships can organically form and thrive may lead to more influential and lasting connections than those established through structured pairings [21-23].

## DISCUSSION

This global scoping review identified 13 studies exploring community-based mentoring interventions for adolescent girls during pregnancy and after birth, mostly in the USA, with two conducted in from sub-Saharan Africa. These interventions, whether formally structured through established programmes (most included studies) or emerging organically as natural mentoring relationships, provided crucial support in areas such as maternal and infant health, educational attainment, stability, and overall psychosocial well-being. Distinguishing between formal and natural mentoring is essential for scalability; natural mentoring studies highlighted active ingredients of successful support, suggesting that formal programmes may be most effective when they foster environments that allow these influential connections to thrive organically. Qualitative perceptions suggest the core mechanism driving these changes may be the provision of sustained, supportive adult relationships, where mentors serve as confidantes, educators, advocates, and role models, offering emotional support, practical assistance, and guidance through complex life challenges. The review findings highlight reports of reduced perinatal and infant mortality and repeat pregnancies; however, the strength of this evidence is influenced by the high proportion of non-comparative designs and potential confounding risks. The most impactful relationships appear to be those that were sustained, resulted from community engagement, were culturally sensitive, and tailored to the specific needs and contexts of the adolescent mothers.

These findings are consistent with broader mentoring literature, which indicates that sustained, high-quality youth mentoring relationships, even with modest effect sizes, could meaningfully improve outcomes among health-related, educational, behavioural, emotional, and social domains [5,28]. Raposa and colleagues’ meta-analysis [5] supports the importance of mentoring duration and relational quality, which this review also highlights as key to successful interventions. Indeed, consistent and supportive mentoring relationships foster mentee development and resilience, influenced by change mechanisms and the personality traits of mentors, mentees, and their match, was also impacted by mechanisms of change and characteristics of mentors, mentees, and their match [8]. Goldner [8] found that traits affected mentoring experiences and outcomes: agreeableness, openness, and extraversion related to positive expectations, with agreeableness also predicting better relationship quality. Agreeableness, conscientiousness, and openness aided adjustment and benefits, while neuroticism and extraversion had negative effects. Traits also influenced how relationship quality impacted self-concept and parental views [30].

However, unlike mainstream youth mentoring programmes, interventions for pregnant and parenting adolescents must navigate complex vulnerabilities, which require sensible and tailored approaches. To be actionable, ‘cultural sensitivity’ should be defined by matching mentors and mentees based on shared lived experiences, such as teen motherhood or HIV status. Furthermore, ‘integration’ was most effective as a ‘brokerage model’, where mentors actively accompanied adolescents to appointments and advocated within health and education systems to bypass structural barriers. Our review suggests that cultural sensitivity is best operationalised by matching mentors and mentees based on shared lived experience, such as teen motherhood or HIV status. Additionally, service integration is most effective when mentors adopt a brokerage model, actively accompanying adolescents to appointments to navigate structural barriers. Stigma was a significant barrier for HIV-infected adolescent mothers impacting their adherence to treatment and access to work opportunities [17], as well as for urban, low-income, African American adolescent mothers [21]. Gender inequality and the associated risk of intimate partner violence and sexual abuse are critical underlying issues that impact some adolescents. Interrupted schooling was a prevalent challenge in the review, as many adolescent mothers drop out or face difficulties balancing education with their new responsibilities, requiring tailored support for academic retention and advancement. Meaningful community engagement and involvement and co-production of mentoring programmes are crucial for developing collaborative approaches that enable effective support and fostering positive change for girls, families and their communities, particularly in LMICs [25,29]. Also, adolescent pregnancy outcomes and mentoring success are inextricably linked to structural factors, including systemic racism, welfare policy, and school exclusion in the USA, as well as distinct implementation challenges like food insecurity and geographical barriers in LMICs.

A few studies were excluded because of their adolescent population definition (<22 or <24 years old), although they reported similar results. For example, one study including Latin adolescent mothers with natural mentors reported better mental health outcomes, such as higher self-esteem and lower levels of depression, particularly among individuals who experienced low early parental acceptance during childhood, suggesting mentors can serve a compensatory role [31]. Another study found formal mentoring enhanced both job prospects and social support for African American and Latina teen mothers, helping them better navigate the challenges they face [32]. Locally developed programmes and those that integrated local mentorship, such as 2YoungLives in Sierra Leone and Mothers2Mothers model adaptation in Malawi [17,25], exemplified how community-rooted and peer-based mentoring models can improve outcomes, reduce stigma, increase service uptake, and foster culturally relevant engagement. These resonate with resilience and social capital theories, which suggest that relational buffers, especially from trusted adults or peers, can mitigate structural inequalities [33]. However, few studies in this review examined long-term effects on life trajectories or intergenerational outcomes [34]. The Maikuru mentoring programme showed increases in high school completion, enrolment in higher education, employment, and notably low rates of repeat teen pregnancy persisted up to five years post-intervention [24]. Although the Mothers2Mothers model found improvements in service uptake, contraceptive use, and reduced repeat pregnancies, the long-term economic situation of participants often remained a challenge, with many still facing poverty and limited access to livelihood resources [17]. This suggests that, while mentoring provides crucial individual support, it may need to be more explicitly integrated with broader structural interventions to address systemic issues like social and economic disempowerment for adolescent mothers. To achieve this, the 2YoungLives model found meaningful and sustained community and stakeholder engagement alongside the mentoring scheme was crucial [25].

A key strength of this scoping review was its comprehensive global search strategy, which aimed to identify evidence across diverse settings and overcome geographical limitations of previous reviews. The inclusion of various study designs, from randomised trials to qualitative explorations, allowed for a broad understanding of mentoring modalities, components, challenges, and reported outcomes. The systematic approach to study selection and data extraction enhanced the rigor and transparency of the review. However, the included studies predominantly came from the USA, with many published over two decades ago. This raised concerns about the relevance and applicability of findings to current contexts, especially in LMICs, where most adolescent pregnancies occur and contexts differ significantly. Furthermore, these older studies may not fully capture digital-era shifts in adolescent social life, such as mobile messaging and tele-mentoring, which present distinct opportunities and safeguarding challenges for modern intervention applicability [34]. Some studies lacked rigorous designs, standardised outcomes, or sufficient follow-up, and variability in intervention content and delivery further complicated cross-study comparisons and scalability. Methodological limitations in several studies, such as small samples, lack of control groups, short follow-up periods, and inconsistent outcome measures, also limited comparability. To enhance impact, future studies should prioritise more randomised trials, including in humanitarian and rural low-resource settings, with longer follow ups to measure health and well-being and other understudied outcomes (*e.g.* violence exposure, mental health, child development) and adhering to minimum reporting standards to describe interventions. Importantly, research should investigate which specific intervention components are most cost-effective for scale-up, helping to isolate the effects of relational bonds from tangible resource transfers. Future studies should also evaluate the cost-effectiveness of different mentoring models and how these can be integrated and scaled up with existing systems. Research should also focus on deeper exploration of mechanisms of change by examining mentor-mentee matching, relational processes and effective strategies for mentor training and supervision. Participatory co-design with adolescent mothers can ensure interventions resonate with their realities and priorities.

## CONCLUSIONS

Community-based mentoring interventions hold significant promise for supporting adolescent girls during pregnancy and after birth. When provided with trust, respect, and ongoing support, mentoring could address both immediate and long-term health and development needs. Yet robust and globally representative evidence is still needed to maximise their potential.

## Additional material


Online Supplementary Document
