# Supplementary material for: A global scoping review of community-based mentoring interventions for adolescent girls during pregnancy and after birth
Source: J Glob Health. 2026 Jun 2;16:04135. doi: 10.7189/jogh.16.04135 (PMC13225466; doi:10.7189/jogh.16.04135)
Supplement: Online Supplementary Document [file jogh-16-04135-s001.pdf]

Supplement to: Fernandez Turienzo C, Kamara M, November L, Kamara P, De Backer K, Rayment-Jones H, Kingsford AM, Williams PT, Sandall J. A global scoping review of community-based mentoring interventions for adolescent girls during pregnancy and after birth. J Glob Health. 2026;16:04135.

Text S1: Search terms, keywords and strategies

Research question: What theoretical evidence supports the use of community-based mentoring interventions for pregnant and/or parenting adolescent girls?

Keywords			
**Pregnant/Parenting**	**adolescent**	**mentoring**	**community**
Pregnancy	child	Mentor*	Community based
Pregna*	(adolescent* or girl* or youth* or child or children or teen* or minors or juvenil* or pubert* or pubescen* or pre-pube* or kid or kids or underage*)	(Mentor* or mentee*) Sponsor*	community based or (community adj3 based
Parent*			
mother			

EMBASE

1. exp pregnant woman/
2. exp pregnancy/
3. exp expectant parent/
4. exp adolescent parent/
5. exp mother/
6. exp adolescent mother/
7. exp expectant mother/
8. (pregnan* or parent* or mother*).mp
9. 1 or 2 or 3 or 4 or 5 or 6 or 7 or 8
10. exp adolescent/
11. exp child/
12. (adolescen* or girl* or young or youth* or child * or teen* or minor* or juvenil* or pubert* or pubescen* or pre-pube* or kid or underage*).mp
13. 10 or 11 or 12
14. exp mentoring/
15. exp mentor/
16. (Mentor* or mentee* or sponsor*).mp
17. 14 or 15 or 16
18. exp community/
19. exp community program/
20. community.mp.
21. (community based or (community adj3 based)).mp
22. 18 or 19 or 20 or 21
23. 9 and 13 and 17 and 22
24. limit 23 to (female and humans)
25. limit 23 to dc=20220720-20230623

Medline

1. exp Pregnant woman/
2. exp Pregnancy/
3. Pregnancy/ or exp Pregnancy in Adolescence/
4. exp Parents/
5. exp Parenting/
6. exp Mothers/
7. (pregnan* or parent* or mother*).mp
8. 1 or 2 or 3 or 4 or 5 or 6 or 7
9. exp Adolescent/
10. exp Adolescent Mothers/
11. exp Adolescent Behavior/
12. exp Adolescent Development/
13. exp Adolescent Health/
14. exp Adolescent Health Services/
15. exp Psychology, Adolescent/
16. exp Adolescent Psychiatry/
17. exp Child/
18. (adolescen* or girl* or young or youth* or child * or teen* or minor* or juvenil* or pubert* or pubescen* or pre-pube* or kid or underage*).mp.
19. 9 or 10 or 11 or 12 or 13 or 14 or 15 or 16 or 17 or 18
20. exp Mentors/
21. exp Mentoring/
22. (Mentor* or mentee* or sponsor*).mp.
23. 20 or 21 or 22
24. exp Community Health Services/
25. community.mp.
26. community based.mp.
27. (community based or (community adj3 based)).mp
28. 24 or 25 or 26 or 27
29. 8 and 19 and 23 and 28
30. limit 29 to (female and humans)

Global Health

1. pregnant adolescents/
2. pregnant women/
3. pregnancy/
4. parents/
5. mothers/
6. (pregnan* or parent* or mother*).mp
7. 1 or 2 or 3 or 4 or 5 or 6
8. adolescents/
9. children/
10. youth/
11. disadvantaged youth/
12. rural youth/
13. youth programmes/
14. (adolescen* or girl* or youth* or young or child* or teen* or minor* or juvenil* or pubert* or pubescen* or pre-pube* or kid or underage*).mp
15. 8 or 9 or 10 or 11 or 12 or 13 or 14
16. mentor.mp

17. mentoring.mp
18. (mentor* or mentee* or sponsor).mp.
19. 16 or 17 or 18
20. communities/
21. community based.mp.
22. (community based or (community adj3 based)).mp
23. 20 or 21 or 22
24. 7 and 15 and 19 and 23

APA PsycInfo
1. exp Pregnancy/
2. exp Adolescent Pregnancy/
3. exp Expectant Parents/
4. exp Parents/
5. exp Parenting/
6. exp Parenting Skills/
7. exp Parenting Style/
8. exp Authoritarian Parenting/
9. exp Authoritative Parenting/
10. exp Adolescent Mothers/
11. exp Expectant Mothers/
12. exp Mothers/
13. exp Single Mothers/
14. exp Unwed Mothers/
15. (pregnan* or parent* or mother*).mp.
16. 1 or 2 or 3 or 4 or 5 or 6 or 7 or 8 or 9 or 10 or 11 or 12 or 13 or 14 or 15
17. exp Adolescent Attitudes/
18. exp Adolescent Behavior/
19. exp Adolescent Characteristics/
20. exp Adolescent Development/
21. exp Adolescent Health/
22. exp Adolescent Psychiatry/
23. exp Adolescent Psychology/
24. exp Adolescent Psychopathology/
25. exp Adolescent Psychotherapy/
26. exp Child Attitudes/
27. exp Child Behavior/
28. exp Child Care/
29. exp Child Characteristics/
30. exp Child Health/
31. exp Child Neglect/
32. exp Child Self Care/
33. exp Child Support/
34. (adolescen* or girl* or young or youth* or child * or teen* or minor* or juvenil* or pubert* or pubescen* or pre-pube* or kid or underage*).mp.
35. 17 or 18 or 19 or 20 or 21 or 22 or 23 or 24 or 25 or 26 or 27 or 28 or 29 or 30 or 31 or 32 or 33 or 34
36. exp Mentor/
37. mentoring.mp.
38. (Mentor* or mentee* or sponsor*).mp.
39. 36 or 37 or 38

40. exp Communities/
41. exp "Communities of Practice"/
42. exp Community Services/
43. exp Community Involvement/
44. community.mp.
45. (community based or (community adj3 based)).mp.
46. 40 or 41 or 42 or 43 or 44 or 45
47. 16 and 35 and 39 and 46
48. limit 47 to (human and female)

IBBS
pregnan* OR mother* OR parent*
AND
adolescen* OR girl* OR young OR youth* OR child* OR teen* OR minor* OR juvenil* OR pubert* OR pubescen* OR pre-pube* OR kid OR underage*
AND
mentor* OR mentee* OR sponsor*
AND
Community OR community based OR (community adj3 based)

Cochrane Library
#1. pregnan* OR mother* OR parent*
#2. adolescen* OR girl* OR young OR youth* OR child * OR teen* OR minor* OR juvenil* OR pubert* OR pubescen* OR pre-pube* OR kid OR underage*
#3. mentor* OR mentee* OR sponsor*
#4. Community OR community based OR (community adj3 based)
#5 AND #2 AND #3 AND #4
#6. limit #5 to (human and female)

GoogleScholar
(pregnant* OR mother* OR parent*) AND (adolescent* OR girl* OR young OR youth* OR child * OR teen* OR minor* OR juvenil* OR pubert* OR pubescen* OR pre-pube* OR kid OR underage*) AND (mentor* OR mentee* OR sponsor*) AND (community OR community based)
[Custom range: inception – 2025; any result type; sort by relevance]
[Screened first ten pages – 23 potentially eligible]

Open grey
Social Sciences and Humanities and Life Sciences Database
Text word: mentoring
[8 potentially eligible but non]

Trial registries
ISRCT: Intervention: mentoring; Text search: adolescent AND pregnancy [up to 2025]
ANZCTR**:** Intervention: mentoring; Gender: females; Age group: <18 [up to 2025]

Open Access Thesis & Dissertations
OATD: mentoring + pregnant + community [up to 2025]

Text S2: Characteristics of included studies

Study ID: Black 2006 *Reviewers initials: AKM/CFT*

Reference:

Black MM, Bentley ME, Papas MA, Oberlander S, Teti LO, McNary S, Le K, O'Connell M. Delaying second births among adolescent mothers: a randomized, controlled trial of a home-based mentoring program. Pediatrics. 2006 Oct 1;118(4):e1087-99.

Other publications from same study: NA

Methods	**Study design**: randomized control trial.<br>**Primary aim**: to examine whether a home-based mentoring intervention was effective in preventing second births within 2 years of the adolescent mother's first delivery.<br>**Intervention**: a home-based intervention curriculum, based on social cognitive theory, and focused on interpersonal negotiation skills, adolescent development, and parenting (postnatal)<br>**Baseline measurements**: maternal: age at first delivery, education, WIC (Supplemental Nutritional Services for Women, Infants, and Children), paying job outside of home, romantically involved with baby's father. Infant: birth weight, gender<br>**Duration of study**: 1997-1999.
Participants	**Setting**: Baltimore, United States<br>**Inclusion criteria**: (1) Eligibility for mothers also included age < 18 years at delivery, (2) first-time delivery, black race, no indication of cocaine or heroin use in the medical chart, and no chronic illnesses that would interfere with parenting or adolescent development (3) to ensure that early parenting would not be complicated by the infant's health needs, infants of eligible mothers had to be term (<37 weeks) and birth weight <2500 g, with no congenital problems, chronic illnesses, or disabilities.<br>**Total number of participants**: 181 (I = 87 & C = 94) Participants. Follow up at 24 months: I=70 and C=79.<br>**Mean age of participants (years) (SD) (at delivery):** 16.3 (1.02) intervention; 16.3 (0.96) control.<br>**Mean gestational age at first visit (weeks)**: Not Applicable<br>**Mean gestation age at time of intervention (weeks)**: Not Applicable – postnatal<br>**Ethnicity**: Black Americans (100%)<br>**Marital status**: Single mothers (100%)<br>**Socio-economic status**: Poor Socio-economic status<br>**Education**: Intervention = 10.2 (SD 1.35) Control = 10.1 (SD 1.04)<br>**Other lifestyle/health behaviors**: fighting, marijuana use, arrest, sexual partners, STIs (baseline).
Intervention	**Type and content of intervention**: home-based intervention curriculum, based on social cognitive theory, and focused on interpersonal negotiation skills, adolescent development, and parenting. The curriculum was delivered biweekly until the infant's first birthday by college-educated, black, single mothers who served as mentors, presenting themselves as "big sisters.<br><br><u>Components (activities, sessions, characteristics, or behaviours)</u>:<br>- After a baseline evaluation, all of the mothers were given information on community resources for young mothers and their children.<br>- The comprehensive curriculum manual that included 19-lessons (deliverable over 1 year) home-based intervention (the Three Generation Study) - participants could receive 1 lesson per visit or more. After the initial 2 lessons, which introduced the blended themes of adolescent development and parenting, the mentors could vary

the order of the lessons, combine lessons, or repeat lessons, depending on the needs of the participants. The intervention focused on the relationship and negotiation skills between the adolescent mother and her mother (grandmother of the child)

- Thus ome visits were organised every other week until the infant's first birthday, for a maximum of 19 visits
- The young mothers learned what to expect of their infant's first year of life, to interpret their infant's cries and bids for interaction, and to provide developmentally enriching activities. Throughout the intervention, there was a focus on personal values and decision-making regarding subsequent pregnancies, access to birth control, and goal setting, rather than an overt message advising participants to avoid a second birth. Condoms were provided at every contact.
- Mothers were compensated for baseline and follow-up visits.
- In-home follow-up evaluations were conducted when infants were 6, 13, and 24 months, and at each evaluation visit, mothers were provided information on: demographics regarding their education, marital status, living arrangements, romantic relationships, and whether they had given birth since their first delivery (birth rather than pregnancies). Several scales were used to measure: life aspirations; risky behaviors (at baseline, participants reported on their lifetime history of risk behaviors at baseline; behaviors since delivery of their first child at follow ups); forms of birth control; mental health status; academic skills, maternal self-esteem, adolescent-infant relationship; parenting competence; life events in the last year.

<u>Recruitment of mentors</u>: 2 college-educated (degrees in psychology and sociology) black women in their 20s who were single mothers, raising 1 preschool- aged child, and living independently. They presented themselves as "big sisters" who had been through the experience of raising a child but were not authority figures (By portraying themselves as big sisters, the mentors took on a supportive role, rather than an authoritarian role). After extensive training, the mentors participated in weekly supervisory sessions. They each worked 20 –30 hours per week, with a caseload of 15 mothers who were seen twice per month. Participants were given their cell phone numbers. The mentors kept a log of visits completed, together with lessons covered and ratings of mothers' responsiveness, but they did not record cell phone contacts.

<u>Training and supervision of mentors</u>: Extensive training (not specified).

<u>Recruitment of girls</u>: Mothers were recruited from 3 urban hospitals in Baltimore from Sept 1997-Dec 1999. They were approached shortly after delivery and given a brochure explaining the study. Those who expressed interest in enrolling were scheduled to receive a baseline evaluation at home 3 weeks after delivery. More than 83% (181 of 219) of the eligible mothers agreed to participate and completed the baseline evaluation.

<u>Matching</u>: No reported.

<u>Duration of mentoring program</u>: 1 year

<u>Follow ups</u>: Families received home visits every other week until the infant's first birthday, for a maximum of 19 visits (*follow-up evaluations* for data collection were specifically conducted at 6, 13, and 24 months after recruitment).

<u>Safeguarding, support issues</u> (e.g. clean criminal records): Not reported

Type and content of control (if applicable):
The controls were like the intervention in terms of maternal age, education, location, ethnicity, and other related risk factors. After baseline evaluation, families in the control group received no further contact until the evaluation visits

Outcomes	**Outcome considered in the review and reported in the study:**<br><br>Adolescent Risk Behaviours by Second Baby Status at Baseline (3 Weeks After Delivery) and at 24 Months**:**<br>Friends have baby - baseline: Second baby 29; No second baby 19; 24months: Second baby 13; No second baby 13.<br>Fighting - baseline: Second baby 26; No second baby 28; 24months: Second baby 19; No second baby 17.<br>Substance use:<br>Steal - baseline: Second baby 4; No second baby 3; 24months: Second baby 0; No second baby 0.<br>Arrested - baseline: Second baby 30; No second baby 14; 24months: Second baby 4; No second baby 7.<br>Jailed - baseline: Second baby 11; No second baby 3; 24months: Second baby 4; No second baby 2.5.<br>1 sex partner - baseline: Second baby 68; No second baby 62; 24months: Second baby 95; No second baby 92.<br>Sexually transmitted infection - baseline: Second baby 30; No second baby 25; 24months: Second baby 27; No second baby 34.<br><br>Adolescent Mother Baseline Family Formation Psychological Characteristics by Second Baby Status<br>Maternal age at delivery, mean (SD): second baby 16.7(1.0); no second baby 16.2(0.8)<br>Dropped out of school%: second baby 11%; no second baby 5%<br>Breastfeed, %: second baby 19%; no second baby 21%<br>Romantic relationship with father of baby, %: second baby 63%; no second baby 67%<br>Plan to have a second baby in next 5 y, mean (SD): second baby 1.7 (0.9); no second baby 1.9 (1.2)<br>Self-esteem, mean (SD): second baby 3.6(0.3); no second baby 3.4(0.4)<br>Depressed (BDI), % : second baby 44%; no second baby 49%<br>Parenting satisfaction, mean (SD): second baby 4.0(0.8); no second baby 4.6(0.7)<br>Parenting efficacy, mean (SD): second baby 4.8(0.6); no second baby 4.6(0.7)<br>Negative life events, mean (SD): second baby 2.2(1.8); no second baby 2.1(1.7)<br>Positive life events , mean (SD): second baby 4.5(2.5); no second baby 4.7(2.0)<br>Support from infant's grandmother, mean (SD): second baby 3.8(o.7); no second baby 3.5(0.7)<br>Conflict with infant's grandmother, mean (SD): second baby 1.8(0.7); no second baby 1.9(0.8)<br><br>Adolescent Mother 24-Month Family Formation and Psychological Characteristics by Second Baby Status<br>Maternal age at delivery, mean (SD): : second baby 18.8(0.8); no second baby 18.3(1.0)<br>Advanced in education since first delivery, % : second baby 65%; no second baby 77%<br>Married, %: second baby 7%; no second baby 1%<br>Live with partner, %: second baby 33%; no second baby 17%<br>Live with grandmother, %: second baby 54%; no second baby 78%<br>Romantic relationship with father of first baby, %: second baby 54%; no second baby 29%<br>Romantic relationship with new partner, %: second baby 25%; no second baby 56%<br><br>Self-esteem, mean (SD) : second baby 3.7(0.4); no second baby 3.4(0.5)<br>Depressed (BDI 9), %: second baby 31%; no second baby 40%<br>Parenting satisfaction, mean (SD) : second baby 4.3(0.9); no second baby 4.2(0.8)<br>Parenting efficacy, mean (SD) : second baby 4.9(0.7); no second baby 4.8(0.6)<br>Negative life events, mean (SD) : second baby 2.5(2.3); no second baby 3.2(2.8)<br>Positive life events, mean (SD) : second baby 7.4(3.6); no second baby 5.5(2.9)

	Support from infant’s grandmother, mean (SD) : second baby 2.4(0.9); no second baby 2.0(0.9)<br>Conflict with infant’s grandmother, mean (SD) : second baby 1.0(0.8); no second baby 1.1(0.9)<br>Reading and math, mean (SD) : second baby 91.3(11.4); no second baby 92.8(11.4)
Underpinning theory of the intervention	The intervention was based on social cognitive theory, which relies on cultural norms, modelling, and the concepts of self- efficacy and support. Self-efficacy, the belief a person holds regarding her ability to enact specific behaviours, can be enhanced through strategies such as role playing and goal setting, which they incorporated into the intervention. Support represents the feeling of connectedness with family or friends and the perception that others are encouraging of your life choices. We promoted support by conducting the intervention in the home, involving family members as much as possible, and using a mentorship model.
Challenges & strategies, lessons learnt.	Although participants could receive >1 lesson per visit, only 40% of intervention group mothers received > or = 8 visits. As previously reported, mothers were often unavailable, resulting in many missed appointments and multiple reschedules. Reasons for missed appointments varied, often reflecting the complex living situations that characterized the lives of many of the young mothers; occasionally missed appointments were caused by administrative/scheduling demands of home visitors.<br><br>Mothers in the control group were 3 times more likely to have a second birth compared to mothers who received 2 intervention visits, raising the question of how the intervention worked. Indeed, most mothers did not receive the bulk of lessons. Possible explanations:<br>1) mothers benefited from the support of a mentor e.g., young mothers frequently contacted mentors (who had cell phones), and mentors often assisted with interpersonal disputes between the mothers and either other family members or partners. This assistance was often provided outside of the regularly scheduled home visits.<br>2) Another possibility is that the information in the initial lessons was powerful enough to enable the mothers to avoid a second birth. The first 2 lessons focused on negotiation strategies, parenting skills, and “listening” to infants’ cues. In the first lesson, mothers viewed and received a 15-minute videotape that featured adolescent mothers with their children, partners, and mothers talking about strategies to avoid conflict, to promote caregiving, and to incorporate personal values into goals. The videotape, which was made by a group of adolescent mothers who had been nominated by the administration of a local school for pregnant and parenting girls for their success in academic performance, citizenship, and parenting behaviour, was well received by the young mothers.<br>3) adolescents benefited from an approach that appealed to both their egocentrism and their emerging autonomy. Operating from the principles of social cognitive theory, the videotape enabled the young mothers to identify and model from successful peers. At the same time, mentors provided the opportunity for the young mothers to view a successful future.<br><br>Only 1 of the mothers who received 6 home visits had a second infant within 2 years. As the number of visits increased, the young mothers were exposed to more content and to the ongoing support of the mentor, perhaps helping them build the desire and skills needed to avoid a second birth. However, it is also possible that the mothers motivated to avoid a second birth may have been motivated to participate in the intervention. The CACE model reduces the likelihood that the difference in second births could be it is possible that a mentorship model that includes a structured intervention, along with a strong focus on building a supportive relationship, may be an effective strategy in

	reducing second births. The findings also suggest that a relatively brief intervention may be adequate.<br><br>Main limitations: 1) although intervention group enrolment led to significantly fewer second births than control group enrolment, the numbers were small. In previous reports, we found no effects of the intervention on children's developmental status or on father involvement at 24 months; 2) unsure whether to attribute the positive effects to the curriculum or to the mentoring provided by the home visitors. Because most home visiting programs use paraprofessionals, rather than college-educated single mothers, further investigation is warranted to determine whether similar effects can be obtained with paraprofessionals; 3) no information on abortions and, therefore, cannot rule out the possibility that the reduction in second births among intervention group mothers was associated with elective abortions.
What is new? Conclusions	A home-based intervention founded on a mentorship model and targeted toward adolescent development, including negotiation skills, was effective in preventing rapid repeat births among low-income, black adolescent mothers. The effectiveness of the intervention could be seen after only 2 visits and increased over time. There were no second births among mothers who attended 8 sessions. There was no evidence that risk behaviour or contraceptive use was related to rapid second births. There was some evidence that rapid second births among adolescent mothers were regarded as desirable and as part of a move toward increasing autonomy and family formation, thereby undermining intervention programs that focus on risk avoidance. Findings suggest the merits of a mentoring program for low- income, black adolescent mothers, based on a relatively brief (6 – 8 sessions) curriculum targeted toward adolescent development and interpersonal negotiation skills.
Additional references / Other comments	

**Quality Assessment –Black 2006**	*Reviewer initials: AKM/CFT*
Critical Appraisal	*The Mixed Methods Appraisal Tool (MMAT) 2018 will be used for quantitative, qualitative and mixed-methods studies.*<br><br>**Screening questions**<br>1. <u>Are there clear research questions?</u> — Yes<br>Yes, to evaluate the effect of a teen mentoring program aimed at preventing second birth<br><br>2. <u>Do the collected data allow to address the research questions?</u> — Yes<br>Yes, second pregnancy/birth data was collected at different timepoints (6, 13 and 24 months).<br><br>**MMAT for quantitative RCT**<br>1. <u>Is randomization appropriately performed?</u> — CT<br>Not enough information.

	2. Are the groups comparable at baseline?<br>Yes, there were no differences in maternal age, maternal education, infant birth weight, infant gender, or intervention status.<br><br>3. Are there complete outcome data?<br>Yes, > 82% of girls completed 24 months evaluation.<br><br>4. Are outcome assessors blinded to the intervention provided?<br>Yes, evaluators were unaware of intervention status.<br><br>5. Did the participants adhere to the assigned intervention?<br>Not sure. For, example, only 40% of the intervention group mothers received =or> 8 visits.	Yes<br><br><br><br>Yes<br><br><br>Yes<br><br><br>CT

**Study ID: Bogat 2008**	*Reviewers initials: CFT/XXX*

Reference:

Anne Bogat G, Liang B, Rigol-Dahn RM. Stages of mentoring: An analysis of an intervention for pregnant and parenting adolescents. Child and Adolescent Social Work Journal. 2008 Aug;25:325-41.

Other publications from same study:

*Contacted authors for linked studies or modified RCTs – not available. A linked study did not include mentoring only alternative schools (Bogat, G. A., Liang, B., Caldwell, R. A., Davidson, W. S. II, Bristor, M., Phillips, M., & Suurmeyer, M. (1993). Alternative schools: A school transition for adolescent mothers. *Prevention in Human Services, 10,* 151-168)

Methods	**Study design**: qualitative (within a modified RCT)<br>**Primary aim**: to describe the stages of the relationships that developed between mentors and mentees in a program for pregnant and parenting adolescent, in particular aspects of the relationship that led to problems and premature endings.<br>**Intervention**: mentoring program<br>**Baseline measurements**: age, ethnicity<br>**Duration of study**: Unclear. Publication in 2008.
Participants	**Setting**: an alternative school in a midsize, Midwestern city, US.<br>**Inclusion criteria**: (a) attending the school for at least 7 days, (b) being a first-time parent, and (c) being pregnant at the time of admission.<br>**Total number of participants**: 175 students enrolled at the school during a 4-year period, 68 were eligible to be randomly assigned to the experimental group. Of those eligible, 51 received mentors. Students who did not participate either declined or relocated prior to being assigned a mentor.<br>**Mean age of participants (years) (SD):** Mentees ranged in age from 12 to 18; the average age was about 15.<br>**Mean gestational age at first visit (weeks)**: Not reported.<br>**Mean gestation age at time of intervention (weeks)**: Not reported.<br>**Ethnicity**: 63% African-American; 18% European-American, 12% Latino, 5% Native American, and 2% were Asian American.<br>**Marital status**: Not reported.<br>**Socio-economic status**: Not reported.<br>**Education**: Not reported<br>**Other lifestyle/health behaviors**: Not reported.
Intervention	**Type and content of intervention**: T<br><br><u>Mentors</u>: Fifty-six women from the local community expressed interest in becoming mentors; 42 eventually did so. For those who did not participate, reasons included mentor time constraints (n = 9), lack of a suitable mentor–mentee match (n = 4) and mentee refusal to participate (n = 1). Mentors ranged in age from 21 to 59, the average age was 38.4. Fifty percent (n = 21) of mentors were African American, 36% (n = 15) were European-American. The remaining 14% were Latino (n = 2), North American Indian (n = 2), Asian (n = 1), and Lebanese (n = 1).<br><br><u>Recruitment</u>: Public service announcements, advertisements, and mailings were used to recruit mentors from the local community. In addition, several mentors recruited friends and acquaintances to participate in the program. Mentors had to be females who were also parents. All mentors were screened for participation and underwent a criminal history clearance (required by the school district) before participating in the program.

	Matches: 51 mentor–mentee matches took place over the course of 4 years. About 20% of these matches (n = 11) required transfers to another mentor due to differences in mentor–mentee personality style, mentor relocation, and mentor lack of time. The average number of mentor–mentee matches per mentor was 1.5. The length of matches ranged from 1 to 9 months, with an average length of 6.8 months (SD = 2.6). Approximately 54% of mentor–mentee matches extended the full 9- month period.<br><br>Training: All potential mentors were required to complete a 4-h training seminar that took place over 1 or 2 days, depending on the schedules of the mentors. The training seminar provided information about the needs of pregnant adolescents and adolescent mothers, communication skills, and requirements for participation. Most importantly, the mentors were trained to provide four types of social support to their mentees: emotional; advice and information; practical assistance; and companion- ship. A training manual which explained the four types of support was given to each mentor. Discussion and role playing during the training sessions promoted mentor understanding and experiential learning of these forms of support and the communication skills necessary to convey them.<br>Supervision: mentors received "weekly support group meetings" and "effective supervision by social workers should be sensitive to and" was given.<br><br>Time commitment of mentor: Mentors were expected to sustain a relationship with their mentees for 9 months and were required to make a minimum commitment of 2 h per week to the project. One hour a week was devoted to face-to-face contact with the mentee, while the other hour was scheduled for participation in weekly mentor support meetings.<br><br>Safeguarding: All mentors were screened for participation and underwent a criminal history clearance (required by the school district) before participating in the program.<br><br>**Type and content of control (if applicable):**
Outcomes	**Outcome considered in the review and reported in the study:**<br>Experiences of mentors and mentees were described by mentors and supervisors.
Underpinning theory of the intervention	Not specifically articulated for their mentoring intervention in the methods section. Yes background information on theory of mentoring interventions for youth and pregnant/parenting girls.
Challenges & strategies / lessons	1) In the early stage of the relationship, one of the most salient was the mentor's difficulty in persevering toward the establishment of the mentor–mentee relationship in the face of the mentee's apparent disengagement. Fear of intimacy and rejection seemed to underlie these relationship difficulties. Withdrawal early in the relationship was a significant sign that the relationship would not mature in later stages. Mentees avoided their mentors; mentors withdrew by limiting their attempts to establish contact with mentees. Failure to complete weekly logbooks and lack of attendance at mentor meetings were further manifestations of mentor disengagement. The best indicator of a positive mentor– mentee relationship was continued involvement in both the mentor/mentee relationship and the mentor support group meetings.<br><br>2) In the second stage, relationships between mentors and mentees floundered over conflict. Mentors sometimes felt overwhelmed by the mentees' neediness brought on by the birth of her baby. Relationships in which the mentor had provided much tangible assistance to the mentee seemed most fragile. In these relationships, the mentees were most demanding and the mentors were most frustrated by these demands and had problems with limit-setting.

	3) In the final stage, difficulties on both the part of the mentor and mentee in discussing termination often led to less frequent contact during this time. This, in turn, prevented the chance to negotiate the possibility of an informal relationship with the mentee when the program ended.<br><br>There are several recommendations that might minimize or help mentors and mentees navigate the problems that arose in the relationship during each of the stages:<br>1) more intensive mentor training, with an emphasis on cultural sensitivity and better understanding of interpersonal process involved in youth mentoring relationships (i.e. Testing behavior, distrust, apparent lack of interest, lack of initiative, and rejection).<br>2) Mentors and mentees may benefit from participating in a formal introduction to the relationship. That is, social workers might devote the first month of the relationship to a thorough introduction to the mentor–mentee intervention and the identification of what is expected from both members of the pair. This would provide more opportunity for mentors and mentees to discuss and prepare for the kind of issues that arise in the first stage of the relationship.<br>3) more intensive tracking of mentors' activities by supervisors. Lack of participation on the part of both mentor and mentee was associated with less positive mentor–mentee relationships. Tracking by supervisors would therefore serve as a check on the status of the mentor–mentee relationship. Weekly calls to mentors (in addition to weekly support group meetings) would facilitate early exploration and resolution of problems developing in the mentoring relationship. Some mentors are reluctant to write down or discuss within a group setting some of these more sensitive issues.
What is new?	In summary, one of the keys to a successful and ongoing mentor–mentee connection may be culturally-sensitive communication at each of the various stages of the relationship. Training and tracking by social work supervisors are useful techniques to promote such communication and may assist in the development of these relationships to their fullest potential.
Additional references / Other comments	Experiences of mentors and mentees were only described by mentors and supervisors in the context of their process notes and logbooks (not the girls themselves).

**Quality Assessment – Bogat 2008**	*Reviewer initials: XXX/XXX*
Critical Appraisal	*The Mixed Methods Appraisal Tool (MMAT) 2018 will be used for quantitative, qualitative and mixed-methods studies.*<br><br>**Screening questions**<br>1. <u>Are there clear research questions?</u> Yes<br>Yes, to describe stages of the relationships that developed between mentors and mentees in a program for pregnant and parenting adolescents, particularly failures<br><br>2. <u>Do the collected data allow to address the research questions?</u> CT<br>Data was collected only from mentors and supervisor's logbooks and notes (and none from mentees), difficult to capture their experiences but mentor's perceptions of their experiences<br><br>**MMAT for qualitative studies**<br>1. <u>Is the qualitative approach appropriate to answer the research question?</u> No<br>Maybe - Experiences of mentoring were described by mentors and supervisors in the context of their process notes and logbooks - this qualitative approach used data simultaneously

	obtained from multiple sources and informants and gave voice to mentors' own perceptions of their experiences, as well providing confirmatory input from their supervisors, but not direct experiences of the mentees (key factors may have been missed here). Authors suggest that although it is not certain whether the frequency of mentioning certain experiences represents the frequency of behaviours within the mentoring relationships, it is likely that this frequency relates to how salient certain behaviours are within the mentoring relationships examined in our study.<br><br>2. Are the qualitative data collection methods adequate to address the research question?<br>Review of process notes and logbooks (unclear what data was collected exactly) is not the most rigorous qualitative methodology for the research questions. Qualitative interviews with mentors and mentees would have probably been more adequate to address the research question.<br><br>3. Are the findings adequately derived from the data?<br>Findings and discussion are presented jointly, not easily to see how findings derived from the data collected.<br><br>4. Is the interpretation of results sufficiently substantiated by data?<br>Findings and discussion are presented jointly, not easily to see how findings derived from the data collected.<br><br>5. Is there coherence between qualitative data sources, collection, analysis and interpretation?<br>Probably yes – although this is a relatively old paper, and some of them could have been better articulated in the different sections (paper presents results and discussion jointly) and data analysis as 'Researcher-as-instrument' statement.	 <br><br><br><br><br><br><br>No<br><br><br><br><br><br>CT<br><br><br><br>CT<br><br><br><br>Yes

**Study ID: Carbone 2019**	*Reviewers: AKM/CFT*

Reference:

Carbone NB, Njala J, Jackson DJ, Eliya MT, Chilangwa C, Tseka J, Zulu T, Chinkonde JR, Sherman J, Zimba C, Mofolo IA. "I would love if there was a young woman to encourage us, to ease our anxiety which we would have if we were alone": Adapting the Mothers2Mothers Mentor Mother Model for adolescent mothers living with HIV in Malawi. PloS one. 2019 Jun 7;14(6):e0217693.

Other publications from same study: NA

Herce ME, Chagomerana MB, Zalla LC, Carbone NB, Chi BH, Eliya MT, Phiri S, Topp SM, Kim MH, Wroe EB, Chilangwa C. Community-facility linkage models and maternal and infant health outcomes in Malawi's PMTCT/ART program: A cohort study. PLoS medicine. 2021 Sep 17;18(9):e1003780 (this is all women)

Methods	**Study design**: Qualitative study<br>**Primary aim**: to examine barriers to, and facilitators of, PMTCT care for HIV-infected AGYW in Malawi, and explore strategies for adapting the mothers2mothers (m2m) Mentor Mother Model to better meet AGYW service delivery-related needs and preferences.<br>**Intervention**: Mothers2Mothers Mentor Mother Model<br>**Baseline measurements**: district, age, marital status, pregnancy status,<br>**Duration of study**: 2017
Participants	**Setting**: four districts: Mzimba North (Northern Region), Lilongwe (Central Region), Thyolo (Southern Region), and Mangochi (Southern Region), Malawi.<br>**Inclusion criteria**: To be eligible for participation in the FGDs were to: have documented HIV infection; be between 15 and 19 years of age; be currently pregnant, post-partum and/or breastfeeding (up to 2 years post-delivery); be living in the four regions where the study participants were to be sampled.<br>**Total number of participants**: 72 participants: divided in 16 FGDs and categorised in two groups: 1) those who had experience with m2m programming (8 FGDs, n = 38); and 2) and the control group (8 FGDs, n = 34).<br>**Mean age of participants (years) (IQR):** 17 (2).<br>m2m 18 (1.75); non-m2m: 18.5 (1)<br>**Mean gestational age at first visit (weeks)**: Not reported.<br>**Mean gestation age at time of intervention (weeks)**: Not reported.<br>**Ethnicity**: Black Africans.<br>**Marital status**:<br>Married: all: 57% (41); m2m: 58% (22); Non-m2m: 56% (19)<br>Widow: all: 1% (1) ; m2m: 0% (0); Non-m2m: 3% (1)<br>Divorce/separated: all: 18% (13); m2m: 11% (4); Non-m2m: 26% (9)<br>Never married: all: 18% (13); m2m: 11% (4); Non-m2m: 26% (9)<br>**Socio-economic status**: low socio-economic status/Districts:<br>Lilongwe: All 29% (21): m2m 32% (12), Non- m2m 26% (9)<br>Mzimba North: All 22% (16); m2m 24% (9), Non- m2m 21% (7)<br>Mangochi: All 25% (18), M2M 24% (9) Non- m2m 26% (9)<br>Thyolo: All 24% (17), M2M 21% (8), Non- m2m 26% (9)<br>**Education**: Not specified.<br>**Other lifestyle/health behaviors**: Pregnancy status:<br>Pregnant: all: 32% (23); m2m: 37% (14); Non-m2m: 26% (9)<br>Postpartum & Breastfeeding: all: 47% (34); m2m: 50% (19); Non-m2m: 44% (15)<br>Postpartum/not breastfeeding: all: 21% (15); m2m:13% (5); Non-m2m: 29% (10)<br>**Number of interactions with m2m mentor mothers**<br>1–2 times m2m 32% (12)<br>3–4 times m2m 18% (7)<br>5–9 times m2m 8% (3)<br>10 times m2m 42% (16)

Intervention	**Type and content of intervention:**<br>Mentor Mother Model pioneered by the international non-governmental organization (NGO), mothers2mothers (m2m), employs women living with HIV, who have successfully navigated the PMTCT cascade, to ensure that other mothers living with HIV are engaged with, linked to, and retained in care. Launched in Malawi 2008.<br><br><u>Components (activities, sessions, characteristics, or behaviours)</u>:<br>- m2m Malawi uses the mentor mother model to provide psychosocial support and education to HIV-pregnant women and new mothers on PMTCT. Mentor Mothers provide motivational education and psychosocial support to clients and work as fully-integrated members of the PMTCT and Reproductive, Maternal, Newborn and Child Health (RMNCH) teams in the health facilities in which they are based.<br>- MMs share first-hand knowledge and experience through one-on-one and group education sessions with women at the facility.<br>- M2m has a community cadre of MMs who interact with clients in the community. MMs and CMMs are rigorously trained and paid stipends, with the facility cadre working full-time and community cadre working three days a week.<br>- Retention: m2m's traces women who miss key appointments in the PMTCT cascade and follows them through phone calls and home visits to encourage their return to care. Where a community cadre is deployed, Community MMs work hand-in-hand with MMs at the facility to identify, track and engage clients. Where there is no community cadre, MMs dedicate time each week to trace clients. Staff members are given bicycles, but given to staffing constraints, the m2m limits tracing activities to a 5km radius. To trace clients beyond this radius, m2m coordinates with HSAs and other tracing cadres. At present, m2m traces clients who have missed their 2nd antenatal visit, the six week infant visit including tests, prophylaxis and immunizations, and tests and results at 12 and 24 months.<br>CMMs also engage traditional leaders to increase buy-in and support for PMTCT and RMNCH services, and to reduce stigma and discrimination in the community. Formal linkages are developed through orientation and sensitization workshops and periodic feedback sessions with traditional leaders. Community health talks are organized in consultation with traditional leaders as well.<br>- Adherence: m2m Malawi currently supports adherence through group and one-on-one education and support. The organization is currently preparing to enhance its offerings to support adherence.<br>- Other: m2m engages site staff in quarterly Let's SOAR (Strengthening Outcomes by Analyzing Results) sessions to both improve the quality of services delivered to clients and improve data quality. SOAR is a continuous program quality improvement methodology designed to build m2m staff capacity to collect, analyze and use data for evidence- informed decision making at the site level. SOAR utilizes a statistically robust sampling methodology that is simple, quick, and manual and enables site staff to review logbook data about client outcomes on a quarterly basis. MOH facility staff members are invited to participate in this program, and are engaged in problem-solving.<br>- m2m provides care and support over an enhanced continuum of RMNH care to HIV-positive and HIV-negative women and their families through its Enhanced Program Model. Under the Enhanced Program, MMs provide education on neonatal male circumcision and referral; tuberculosis pre-screening and education; nutrition support and mid-upper arm circumference measurement; and cervical cancer education and referral.<br><br><u>Recruitment of mentors</u>: m2m's program model identifies and employs mothers living with HIV and with recent PMTCT experience to serve as Mentor Mothers to pregnant women and recently diagnosed HIV-positive pregnant women and new mothers.<br><br><u>Training and supervision of mentors</u>: MMs are mothers living with HIV who are trained, and empowered to work alongside doctors and nurses in understaffed health centres as paid

	members of the healthcare team. Some mentor mother cadres are trained to recognize early warning signs for clinical deterioration in order to decrease maternal mortality.<br><br>Recruitment of girls: m2m logbooks, clinic registers. Uptake: MMs at facilities provide group health talks and one- on-one education to women in antenatal facilities to promote uptake of services. CMMs are deployed to communities within the catchment areas for facilities in which m2m provides services. The community cadre goes door-to-door and conducts community health talks to identify new clients, provide education and support, and link women to facility-based care. CMMs and MMs reinforce messaging to couples at the facility and within the household setting to promote the understanding of reproductive choices, family planning methods, and dual protection. m2m also refers partners and wider family members to facilities for HTC.<br><br>Matching: Not reported.<br><br>Duration of mentoring program: Unclear.<br><br>Follow ups: Not reported.<br><br>Safeguarding, support issues (e.g. clean criminal records). Not reported.<br><br>**Type and content of control (if applicable):** usual care, no mentoring programme (however, amongst the control some reported to have interacted with those in the m2m mentoring).
Outcomes	**Outcome considered in the review and reported in the study:**<br><br>Poverty, stigma, food insecurity, lack of transport, and absence of psychosocial support were crosscutting barriers to PMTCT engagement. While most participants highlighted resilience and self-efficacy as motivating factors to remain in care to protect their own health and that of their children, they also indicated a desire for tailored, age-appropriate services. FGD participants indicated preference for support services delivered by adolescent HIV-infected mentor mothers who have successfully navigated the PMTCT cascade themselves.
Underpinning theory of the intervention	The researchers suggest that having access to a peer-mentor of the same age group of similar circumstance, living in the same community and being part of the health force for PMTCT services can help increase retention, reduce psychological barriers, and adherence to ART amongst HIV-infected AGYW in Malawi.<br>Mothers2mothers (m2m) works to close the gap between the increasing availability of PMTCT and health services and the lack of uptake of these services by the women and children who need them most. Since 2008, m2m has worked in Malawi to empower pregnant women and mothers to live healthier lives by educating and supporting them to access and adhere to lifesaving interventions for themselves and their babies.
Challenges & strategies, lessons learnt	Although not explicitly mentioned by adolescent participants, issues of gender inequity underlined their reported experiences of discrimination, structural barriers to service access, and economic marginalization. Previous work from sub-Saharan Africa has documented the association between gender inequality for AGYW and their increased risk of intimate partner violence and adverse sexual and reproductive health outcomes, including incident HIV infection, thus comprehensive gender empowerment interventions needed. Deeply intertwined with gender inequality were participant descriptions of economic vulnerability and lacking sufficient financial support to meet their basic needs. This finding suggests a gap in research and programming around PMTCT-integrated socioeconomic interventions, such as cash transfer programs, micro-finance interventions,

	and income generating activities designed to economically empower HIV-infected adolescent mothers and their families. Limited evidence from SSA on the association between financial incentives and other economic empowerment interventions and care retention for PMTCT clients.<br><br>Recommendations to adapt m2m for adolescents' mothers:<br>1) preference for engaging with mentor mothers of a similar age with whom they could relate (e.g. "adolescent peer" mentor mothers)<br>2) peer mentor mothers should have experience successfully navigating the PMTCT cascade, sustaining ART for at least 2 years, and surmounting those health-system, psychosocial, and structural barriers disproportionately affecting HIV-infected adolescent mothers (dual stigma from being HIV-infected and a young mother, challenges of staying in school).<br>3) adolescent mentor mother cadre must safeguard beneficiary privacy and confidentiality at all times, as a few participants described their concerns about inexperienced mentor mothers potentially not keeping sensitive health information secret.<br>4) to facilitate confidentiality, promote care navigation, and provide psychosocial counselling, the same cadre of adolescent mentor mothers must engage beneficiaries with support both in the community as well as in the facility, serving as a bridge between home and clinic.<br>5) to address barriers of poverty and gender inequality, adolescent mentor mothers can serve as a resource to link adolescent mothers to economic opportunities and interventions in their communities. Such interventions can be incorporated into existing programs (I,e m2m).<br><br>Limitations: recruited participants at health facilities, we may not have been able to reach AGYW without access to, or who completely disengaged from, public health services. At the two of the sites, we were only able to enrol three participants per FGD, which can limit the diversity of opinions and input available for those FGD data. While we did successfully enrol adolescent mothers as young as 15 years (n = 4 participants), the perspectives of the youngest adolescent mothers may have been underrepresented. As this implementation research was intended to help adapt the m2m Mentor Mother Model, our results may not be fully generalizable to all PMTCT support programs. However, we expect our findings to have general relevance to PMTCT programs involving other community-based or task-shifted lay health providers, such as peer educators and community health workers.
What is new?	HIV-infected adolescent mothers expressed a preference for peer-led, non-judgmental PMTCT support services that bridge communities and facilities to pragmatically address barriers of stigma, poverty, health system complexity, and food insecurity. Future research should evaluate implementation and health outcomes for adolescent mentor mother services featuring these and other client-cantered attributes, such as provision of livelihood assistance and peer-led psychosocial support.
Additional references / Other comments	Teasfale C, Besser M. Enhancing PMTCT programs through psychosocial support and empowerment of women: the Mothers2Mothers model of care. S Afr J HIV Med. 2008; 1:60–64.<br><br>Chihana NK, Chapman S, Sandfolo S, Scheepers E, Schmitz K (2016) Uptake of prevention of mother- to-child transmission (PMTCT) services by adolescents and young women compared to older women: evidence from a retrospective cohort in six Sub-Saharan African countries. Poster presented at: 21st International AIDS Conference; 18–22 July 2017; Durban, South Africa.

**Quality Assessment – Carbone 2019**	*Reviewer initials: AMK/CFT*

Critical Appraisal	*The Mixed Methods Appraisal Tool (MMAT) 2018 will be used for quantitative, qualitative and mixed-methods studies.*<br><br>**Screening questions**<br>1. Are there clear research questions? — Yes<br>Yes, to examine barriers to, and facilitators of, PMTCT care for HIV-infected AGYW in Malawi, and explore strategies for adapting the mothers2mothers (m2m) to better meet service delivery-related needs and preferences.<br><br>2. Do the collected data allow to address the research questions? — Yes<br>Yes, data collected from 16 focus group discussions with more than 70 participants<br><br>**MMAT for qualitative studies**<br>1. Is the qualitative approach appropriate to answer the research question? — Yes<br>Yes, thematic analysis to assess major and minor themes and to compare findings.<br><br>2. Are the qualitative data collection methods adequate to address the research question? — Yes<br>Yes, sampling variations in terms of district, age, intervention/non-intervention.<br><br>3. Are the findings adequately derived from the data? — Yes<br>.Yes and quotations presented.<br><br>4. Is the interpretation of results sufficiently substantiated by data? — Yes<br>Yes, it looks interpretation of results derived from the data and the study details quality control review by the research team and data saturation checks.<br><br>5. Is there coherence between qualitative data sources, collection, analysis and interpretation? — Yes<br>Yes, there appears to be coherence about data collected at FGD, the thematic analysis and the findings presented.

**Study ID: Estrada 1997**	*Reviewers initials: CFT/AHK*

Reference:

Estrada L. Program evaluation of a mentoring program for teen mothers" (1997). Theses Digitization Project. 1301.

Other publications from same study: NA

Methods	**Study design**: mixed-method exploratory evaluation<br>**Primary aim**: to evaluate the impact of a mentoring program's accountability, utilization, and entrenchment. Main goals: increase the delivery of healthy infants, decrease the incidence of future unplanned pregnancies, and to provide motivation for adolescent women to develop the expansion of their horizon which includes self-actualization.<br>**Intervention**: teenage mentoring intervention<br>**Baseline measurements**: gender, age, ethnicity, primary language, marital status, years of education, school degree, living arrangements, income source, family income.<br>**Duration of study**: 1996-1997
Participants	**Setting**: California, USA.<br>**Inclusion criteria**: 1) Teenagers: less-than/equal to 18yrs must be pregnant and have referral tickets from 2 local hospitals or 3 district schools; or self-referrals. 2) steering committee members, 3) coordinator, 4) mentors<br>**Total number of participants**: Planned: 1 program coordinator, 8 steering committee members, 18 mentors, 18 program participants (mentees). Achieved: 34 participants: 10 steering committee members; 1 coordinator, 14 mentors and 9 mentees.<br><br>Description of Demographic Data for Steering Committee.<br>**Gender: m**ale 1 (11.1%); female 10 (98.9%)<br>**Age:** 32-39: 5 (45.5%); 40-53: 6 (54.5%)<br>**Ethnicity: White:** 4 (36.4); Mexican American: 6 (54.5); Other: 1(9.1)<br>**Primary Language:** English: 7 (63.6%); Spanish: 4 (36.4%)<br>**Marital Status:** Single: 3, (27.3%); married: 7 (63.6%); common law/ free union: 1 (9.1%)<br>**Years of Education** 16-18: 8 (72.8%); 19-22: 3 (27.2%)<br>**School Degree:** Associates/Bachelors: 5 (45.5%); Masters/Other: 6 (54.5%)<br>**Family Income:** 10.000-20,000:2 (18.2%); 20.001-40,000:1 (9.1%); 40,001-above:8 (72.7%)<br><br>Description of Demographic Data for Mentors.<br>**Gender:** male 0 (0.0%); female 14 (100.0%)<br>**Age:** 20-30: 4 (28.6%): 31-40: 6 (42.8%); 41-55: 4 (28.6%)<br>**Ethnicity:** White: 4 (28.6%); Mexican American: 9 (64.3%); Other: 1 (7.1%)<br>**Primary Language:** English 9 (64.3%); Spanish 2 (14.3%); Bilingual 3 (21.4%)<br>**Marital Status:** Single: 5 (35.7%): married 8 (57.1%); divorce 1 (7.1%)<br>**Years of Education:** 12-13: 3 (21.4%); 14-16: 8 (57.1%); 17-18: 3 (21.5%)<br>**School Degree:** No HS Graduate 2 (14.3%); HS Graduate 4 (28.6%); Associates/Bachelors 6 (42.9%); Masters/Other 2 (14.2%)<br>**Family Income** 10.000-20,000: 2 (14.3%); 20.001-30,000: 4 (28.6%); 30,001-40,000: 1 (7.1%); 40,001-50,000: 3 (21.4%): 50,001-above: 4 (28.6%)<br><br>Description of Demographic Data for Mentees<br>**Age:** 13-15: 1 (11.2%); 16-17y: 4 (44.4%); 18-19: 4 (44.4%)<br>**Ethnicity:** White 1 (11.1%); Mexican American 8 (88.9%)<br>**Primary Language:** English 4 (44.4%); Spanish 5 (55.6%)<br>**Marital Status:** single: 7 (77.8%); married: 1 (11.1%): free union: 1 (11.1%)<br>**Number of Children:** 0-1: 7 (77.8%); 2-3: 2 (22.2%)

	**Years of Education:** 2-8: 2 (21.4%); 9-10: 3 (57.1%): 11-12: 4 (21.5%)<br>**Living Arrangements:** Parents 4 (44.4%); relatives 1 (11.2%); husband/partner: 2 (22.2%; own: 2 (22.2%)<br>**Employed**: 1 (88.9%); non-employed: 8 (11.1%)<br>**Income Source:** parents: 4 (44.4%) husband/Partner: 2 (22.2%); own: 1 (11.2%); AFDC 2 (22.2%)<br>**Family Income:** 10,000-20,000: 6 (66.7%); 20,001-30,000:3 (33.3%)<br>**Other lifestyle/health behaviours**: Not reported.
Intervention	**Type and content of intervention**: volunteer mentors are trained to work with pregnant and parenting adolescents for up to one year. The program is guided by a steering committee composed by eight members (seven women and one man). These committee members are community stakeholders who are concerned with the high teen pregnancy rate, and have a personal commitment to support the program. Some people are voluntarily donating their time and others serve as representatives of community agencies. The program coordinator is responsible for the delivery and acquisition of applications for both, mentors and mentee referrals, and organising the recruitment and training of mentors.<br><br><u>Components (activities, sessions, characteristics, or behaviours)</u>:<br>- Mentors are expected to provide teen mothers with support, guidance, referrals to community resources. Mentors may also serve as educators, advocates, brokers, and confidants.<br>- The program trained mentors who come from all walks of life and are members of the local community. They mentor one adolescent woman throughout their pregnancy and possibly through the first year after the baby's birth. During this process, the mentor becomes the main link between young girl and service providers. Also, the mentor acts as an advocate to facilitate/support the teen.<br>- The mentor assesses the adolescent's individual needs and becomes a primary source of information. Mentors guide mentees on questions regarding drug use issues, health care, school and social service programs, child development, contraceptive methods and other areas.<br>- During the first year of the infant the mentor continues to assist the mother to receive health care for herself and the infant. She also encourages academic retention, helps to prevent unplanned and repeat pregnancies, assists in exploring and planning for employment, training and enhances protective factors.<br>- If a mentor decides to continue their mentoring commitment, they may do so. However, if at the end of their first year of service, they decide not to continue, the mentee may be assigned to a new mentor.<br>- A buddy system provides supportive assistance to mentors. This buddy system pairs one steering committee member with two or three mentors for guidance, support, information and assistance.<br>- An additional support measure for mentors are the bi-annual gatherings where the mentors and the steering committee members socialize and build rapport. In addition, mentors gather twice a year to meet with other mentors and steering committee members. These gatherings are also used to recognize the mentors' commitment and contributions to the program.<br><br><u>Recruitment of mentors</u>: The program coordinator is responsible for the initial face-to-face interviews with volunteer unpaid mentors to provide general information about expectations and commitment to the program. Four mentors who had been teen mothers themselves or had a daughter who was a teen mother.<br><br><u>Training and supervision of mentors</u>: Once the initial interview is complete, the prospective mentors will participate in a half a day (4 hour) training program. Also, existence of buddy system for mentors and bi-annual gatherings with mentors and steering committee members.<br><br><u>Recruitment of girls</u>: Mentee referrals are received primarily from two major local hospitals and three local school districts. The only criterion for participation is for the teen mother to be pregnant, have a referral from a local community agency, and voluntarily agree to commit and

	follow the program's expectations. Teen mothers can also be self-referred and make contact with the program directly. Mentees are also interviewed by the program coordinator, prior to being assigned. Those initial interviews are for the purpose of successfully matching of mentor and mentee.<br><br>Matching: Based on the initial interviews and training, a mentee is assigned to a trained mentor, by the program coordinator.<br><br>Duration of mentoring program: The duration of the mentoring commitment is of at least one year.<br><br>Follow ups: Not applicable as follow-ups were not reported in the document.<br><br>Safeguarding, support issues (e.g. clean criminal records). Not reported issues on mentors' criminal status/background checks.<br><br>**Type and content of control (if applicable):** not applicable.
Outcomes	**Outcome considered in the review and reported in the study:**<br><br>**Steering Committee Level:**<br>1) Stage of the program: Most of the participants agreed that the accomplishment of initial program goals were met: the program was able to recruit and train mentors (including development of policies, procedures, training handbook, additional funding) and matched the first 18 mentors with mentees.<br>2) Program strengths: The majority of the participants agreed that the strongest part of the program was the steering committee members; their commitment, interest, and dedication to these community stakeholders was the foundation of the program. Also, mentors' time, commitment and community support were identified as strength.<br>3) Program weaknesses: Recruitment of mentors seemed to be the greatest challenge. Involving recruits takes time and commitment by community members, and this problem was closely related to the marketing and fundraising activities. Added to this problem was the absence of a consistent, “qualified", full-time coordinator, and the potential for decreased communication/cohesiveness of the Steering Committee members.<br>4) Barriers to growth and development: most participants agreed that the greatest problem was the lack of time volunteers devoted to the program (appeal and interest to working/professional women who can be good mentors, but they were already busy volunteering with other agencies). Another challenge was finding a program coordinator with the knowledge, experience, commitment and interest in the program.<br>5) Program accessibility: Numerous resources were identified by participants as necessary for the program to grow and develop e.g., resources to hire a full-time coordinator, the need to add clerical support services, money allocation to pay for supplies, equipment, and for marketing. Though, the majority of the participants agreed that the program had very good accessibility, the lack of mentors for matching of new referrals could become a problem.<br>6) Program accountability: Participants agreed that a certain measure of accountability seemed to already be in place, but the existing measures were not clear or not being consistently enforced e.g., reporting of funding sources, recording of services provided, formal recording and approval of minutes, maintenance of attendance records for meetings/training sessions.<br>7) Future plans: Participants agreed that a future plan was necessary to ensure the program's growth and expansion, including e.g. measurement of success and satisfaction, adapting the model to the school/hospital system, expanding the program for young fathers, developing networking relationships with other agencies as well as with mentees and their families.<br><br>**Mentors level**:<br>1) Reasons for becoming a mentor: Half of the mentors interviewed were volunteering because they saw the need to mentor a young woman and felt a need to help. Four mentors had been teen

mothers themselves or had a daughter who was a teen mother. Participants also felt a need to encourage mentees to become mentors of other young women, to open the communication between adults/adolescents and to promote mentoring as an effective intervention for reducing teen pregnancy.

2) Mentor and mentee relationship: 11/14 mentors had established a friendly- relationship with a mentee, and provided information about resources, they listened, guided, and encouraged their mentee to continue in school. 3 mentors failed to establish a rapport with their mentees.

3) Problems encountered as a mentor: Mentors were mostly concerned about the lack of knowledge and clear roles of mentors/mentees. This problem, they felt, was made worse by the lack of guidance and assistance to mentors in their relationships with their mentees. Other challenges: lack of telephone service by mentees, lack of networking and cooperation among mentors, lack of resources geared to teen mothers' needs, and conflicts in mentees' family members.

4) Positive experiences as a mentor: 13/14 mentors reported having more than one positive experience. The most common valuable experience was the opportunity to watch the mentee's growth and development. Other positive experiences identified by mentors was to see the impact and changes occurring with the mentee. One mentor noted: "It was a pleasure being the birth coach for my mentee," others discussed the reciprocity in learning and the insight mentees provided about the adolescent stage of development.

5) Lack of resources identified by mentors: Participants were divided on how best to use the resources for becoming better mentors. 5/14 mentors felt that it would be useful to have an updated community resource list of services used by teen mothers. While 4/14 mentors felt there were enough community resources to meet their needs. The remainder expressed their desire to become more familiar with the procedures and requirements to access these community resources.

6) Suggestions for training improvement: Participants expressed mixed opinions.

4/14 stated that training was not useful for them while the rest 10/14 T stated that it was very useful and informative. Some suggestions for improvement: follow-up instruction by short mini-trainings, teen mothers be invited to share their experiences and make recommendations, use of role playing as a teaching activity, use of mailings with written information on different topics, or the opportunity for mentors to attend other community agencies' training programs.

7) Mentor and mentee matching process: 8/14 mentors expressed happiness and were pleased with the matching process, while 6/14 experienced some difficulties such as incompatibility, long waiting period before matching occurred, and in some case, mentors had to pursue own matching.

8) Suggestions for program improvement: sponsoring gatherings, outings, or group baby showers for the mentees; finding ways to improve communication between mentors and Steering Committee members; and for mentors to use the committee as a support and as an immediate source for feedback.

Mentees Level:

1) Mentor and mentee relationship: All of the mentees felt very comfortable and felt they had established a good relationship with their mentors. They all described their relationship in a positive way stating that their mentor acted as a guide, helped them, listened to them, supported them, advised them and encouraged them. Only 1 mentee mentioned she felt conflicted over the lack of time available to meet with her mentor.

2) Areas influenced by mentoring process: Most mentees stated they were influenced by the support, guidance, and communication they received from mentors. 4 said they were positively influenced by the encouragement and assistance they each received e.g. helping them to achieve their educational goals.

3) Resources used by mentee: These most useful services included college and other schools information, Medical and Aid for Families with Dependent Children application process, Catholic Charities, Women Infants and Children (WIC), immigration services, child care, pediatricians. Child Protective Services, voucher for babys' products and acquisition of baby's birth certificate.

4) Mentee's perception of program: mostly positive. All the felt that the assistance, advice and knowledge they gained from a more experienced person was invaluable. They appreciated the suggestions and attention that was afforded them during this period of time. They felt supported by their mentors.

	5) Suggestions to improve the program: social gatherings to expand the opportunity to encounter other young mothers and their children; provision of day care in the afternoons to attend training schools to acquire job skills. One mentee asked for services to help her deal with her depression.
Underpinning theory of the intervention	Proposed in this study was that this mentoring program would have a significant influence among the teen program participants (mentees) to prevent future unplanned pregnancies, increased delivery of healthy infants and academic retention. It was proposed that mentors would provide a positive role model, guidance and support to mentees who lacked meaningful relationships. The mentor was a source of information for those adolescents who we're not familiar with community programs and who could benefit from information on drug use issues, pregnancy, health care and use of contraceptives. Education and networking of community resources was a major component of the mentor's role.
Challenges & strategies, lessons learn.	*Most probably already captured in Outcomes*<br><br>A few barriers were identified to the program's further growth and development. These barriers included volunteers availability, problems with time and commitment, the recruitment of a qualified full-time coordinator and the securing of enough funding to effectively continue running the program. The mentees reported that the program provided support, guidance, encouragement, and assistance. The mentors expressed their satisfaction with the program and felt positive about the contribution it makes to the development and growth of young mothers. Mentors agreed that they were influential and made an impact in the education, emotional support and advocacy for the mentees. All of these benefits were accomplished by community stakeholders and individuals who are concerned with the rapid increase of teenage pregnancy.<br><br>The recruitment of mentors and the replacement of steering committee members, who have resigned or requested a leave of absence due to lack of time, was a concern. This problem was a possible cause in the breakdown of communication between steering committee members, mentors and mentees. Improvement of communication between steering committee members and mentors was also identified as a major problem. The program appeared to be accessible to the target population, but the lack of trained mentors continued to be a problem. When mentees are referred to the program, the waiting period becomes longer than usual when there are no mentors available. Consequently, the mentees' willingness to participate is greatly diminished. Also, their instability and precarious situation forces mentees to change residence and to move often.<br><br>Recommendations: to expand, to grow and to become a viable community resource; to develop an evaluation tool to measure the program's success and participant satisfaction; to introduce the mentoring model to local school districts where similar interventions can be used to reach the population of interest; or to adapt to young fathers; social gatherings; day care or respite to allow for after school attendance and to learn a vocational career or to obtain on the-job-training.
What is new? (conclusions)	The results showed that the mentoring program had accomplished its original goals of training mentors and matching them with mentees.
Additional references / Other comments	NA

**Quality Assessment – Estrada 1997**	*Reviewer initials: CFT/AMK*
Critical Appraisal	*The Mixed Methods Appraisal Tool (MMAT) 2018 will be used for quantitative, qualitative and mixed-methods studies.*<br><br>**Screening questions**<br>1. Are there clear research questions? — Yes<br>Yes, to evaluate the impact of a mentoring program's accountability, utilization, and entrenchment.<br><br>2. Do the collected data allow to address the research questions? — Yes<br>Probably yes, mixed methods data (mainly baseline data + qualitative interview data) from mentees, mentors, steering committee members, and coordinators.<br><br>**MMAT for mixed methods studies**<br>1. Is there an adequate rationale for using a mixed methods design to address the research question? — No<br>Not specifically reported in the text.<br><br>2. Are the different components of the study effectively integrated to answer the research question? — CT<br>Not sure, baseline characteristics are the only quantitative results presented in Tables, and interview data is mainly qualitative (some quant content analysis)<br><br>3. Are the outputs of the integration of qualitative and quantitative components adequately interpreted? — CT<br>.Not sure, results from the interviews analysis are presented mainly qualitatively with some content analysis, but does not appear consistent though the results/interpretation.<br><br>4. Are divergences and inconsistencies between quantitative and qualitative results adequately addressed? — CT<br>Can't really tell as reported in 3 and 4 – difficult to distinguish between qual and quant results (except baseline)<br><br>5. Do the different components of the study adhere to the quality criteria of each tradition of the methods involved? — CT<br>It doesn't look so, but not enough information to judge.

**Study ID: Flynn 1999**	*Reviewer initials 1&2: CFT/LN*

Reference:

Flynn L. The adolescent parenting program: Improving outcomes through mentorship. Public Health Nursing. 1999 Jun;16(3):182-9.

Other publications from same study:

NA

Methods	**Study design**: before and after intervention study<br>**Primary aim**: to analyse the efficacy of a program designed to improve infant outcomes through the enhancement of health practices and parenting skills in low- income, pregnant and parenting adolescents who reside in an urban area and who screened positive for risk of child maltreatment.<br>**Intervention**: an adolescent parenting program that provided intensive home visitation by nursing paraprofessionals, indigenous to the community, and based on theories of mentorship and social support.<br>**Baseline measurements**: maternal age, ethnicity, income level, Initial risk of child maltreatment, mean gestation age at the time of intervention (weeks)<br>**Duration of study**: 2 years
Participants	**Setting**: Newark, New Jersey, United States<br>**Inclusion criteria**: high-risk, adolescent mothers who were: age 18-years-old or younger; Medicaid eligible; residents of Newark, New Jersey; not currently clients of the Division of Youth and Family Services; either pregnant with their first child or within 6 weeks postpartum; and who screened at risk for potential child maltreatment using the Family Stress Checklist (FSC).<br>**Exclusion criteria**: Not specified.<br>**Total number of participants**: 137 adolescents.<br>**Mean age of participants (years) (SD):** 16.89 (1.12)<br>**Mean gestational age at first visit (weeks)**: Not reported.<br>**Mean gestation age at time of start of intervention (weeks)**: 28.84 (11.23). Around 43% started before 26 weeks of pregnancy.<br>**Ethnicity**: African American: 71%; Hispanic: 27% ; other: 2%<br>**Marital status**: Not reported.<br>**Socio-economic status**: Not reported.<br>**Education**: Not reported.<br>**Other lifestyle/health behaviors**:<br>Initial risk of child maltreatment (FSC)*: 100% scored at 25 or above indicating all were at risk for child abuse or neglect. Scores ranged from 25 to 168 with a mean of 41.55 (17.40) at the time of admission to the program.<br><br>**The FSC is a semi-structured interview tool that measures a respondent's risk factors relative to family stress and child maltreatment. Rated risk factors include mental health history, use of drugs and alcohol, support systems and family coherence, history of childhood abuse, coping mechanisms, and knowledge of child development. Each item is rated on a scale ranging from 0 (absence of risk) to 10 (severe risk). Total scores can range from 0 to 100; a total score of 25 or more indicates a respondent is at risk for child abuse. Reliability coefficients have not been reported in the literature.*
Intervention	**Type and content of intervention**: The Adolescent Parenting Program of the Essex Valley Visiting Nurse Association was designed to enhance the parenting skills and maternal health behaviours of high-risk, adolescent mothers; it was based on a Healthy Families America model (National Committee to Prevent Child Abuse, 1997) of intensive home visitation for a maximum period of 3 years.<br><br>Components (activities, sessions, characteristics, or behaviours):

- The program was designed to provide services concurrently to 100 adolescents and their infants and was staffed by 5 paraprofessional family support workers and 2 community health. All program staff received training provided by Healthy Families America in child development, stress management, and child abuse prevention.
- Congruent with a mentorship framework, 3 visits were conducted by paraprofessional family support workers and additional home visits were provided by a community health nurse. Referrals to the program were predominately made through local clinics and health care providers.
- Once admitted to the program, participants were placed on Level I, the first of four levels of mentor intervention. In Level I, characterized by more intensive support, the participants were visited weekly by their family support worker and monthly by their community health nurse. Through example and discussion, the family support worker taught and reinforced parenting skills and healthy behaviours such as making and keeping prenatal and post-partum appointments, smoking cessation, age-appropriate immunization, and family planning. Acting in the role of a ‘‘big sister,’’ the family support worker provided the adolescent with emotional and social support. She frequently accompanied the adolescent to health care or community resource appointments, guiding the adolescent through the local health and human services system. Additionally, the family support worker helped the adolescent identify short- and long-term goals as well as the basic, instrumental behaviours needed to meet those goals, such as school attendance or skills development. Through these activities, the family support worker fulfilled the mentorship function of teacher, guide, advocate, and advisor, providing education, information, support, and nurturance.
- Although most adolescents remained at Level I for at least 1 year, achievement of standardized program goals (such as ‘‘no crisis in home for past 30 days’’), would allow participants to be promoted to Level II. At Level II, family support worker visits were decreased to every other week; at Level III, visits were decreased to monthly; and finally at Level IV, visits were decreased to quarterly.
- The type of support functions provided by the family support worker remained constant across the four levels; the only difference between the levels was the frequency of home visitation.
- Throughout the program, the community health nurse visited monthly to assess health needs and instruct the adolescent in nutrition, stages of labor, infant care, child development, and stress management.
- The community health nurse and family support worker held weekly conferences to review each adolescent’s status, needs, and continuing plan of care.

Recruitment/training and supervision of mentors: paraprofessional family support workers were specially trained certified home health aide or nursing paraprofessional who was indigenous to the community and who served as the family support worker or mentor. Two community health nurses (baccalaureate-prepared registered nurses specially trained who also provided direction and supervision to the paraprofessional family support worker). All program staff received training provided by Healthy Families America in child development, stress management, and child abuse prevention.

Recruitment of girls: Potential participants were predominately referred to the project by local health care providers. Referrals were accepted from any source, however, including self-referral. A family support worker then contacted the potential participants and explained the project scope, duration, and inclusion criteria. A home visit was scheduled by the family support worker to further discuss the project and complete initial screening, including administration of the Family Stress Checklist (FSC). Signed consents to participate in the program as well as the accompanying research were obtained from the adolescent and her parent or guardian.

Matching: Not reported.

Duration of mentoring program: 24 months

	<u>Follow ups</u>: Of the 137 adolescents who had given birth, 44 were lost to attrition by the end of the study period.<br>Of the 44 who left the program, the length of participation ranged from 1 month to 24 months with a mean of 9.75 months (*SD* 4 6.16). Of those who left, only 20% refused further participation, while 25% moved out of the catchment area, 22% (*n* 4 12) moved and were unable to be located, and 13% completed Level IV and were discharged by the nurse. Among those who left the program, the mean age of the baby at time of termination was 7.14 months (*SD* 4 5.28) and ranged from 1 month to 24 months. No differences in demographics between those who left the program and those who remained active.<br><br><u>Safeguarding, support issues</u> (e.g. clean criminal records)<br><br>**Type and content of control (if applicable):** NA
Outcomes	**Outcome considered in the review and reported in the study:**<br>Findings were compared to local or national data<br>Mean (SD) length of gestation: 39.27 weeks (41.55)<br>Low birthweight: program: 4.6%; local and national: 13.5% and 9.42%<br>Infant mortality rate: program: 0 local and national: 15.8 per 1,000.<br>Child neglect cases: program: 4 (2.91%) local and national: 11%.
Underpinning theory of the intervention	'Home visitation programs utilizing paraprofessionals who are indigenous to the community have been found to be effective in improving maternal health practices within high-risk families (Bradley & Martin, 1994). These programs are frequently based on a model in which the home-visiting paraprofessional serves as a mentor, or role model, to the at-risk mother, providing social support and nurturance as well as education regarding child development and parenting (Mitchel & Donnelly, 1993). Mentorship, as conceptualized by Vance (1982), is one theoretical framework that may provide an explanation for the efficacy of these programs'.<br><br>**Mentorship**<br>'Vance describes a mentor as an older, more experienced person who guides and nurtures a less experienced person. Based on a parental model, mentorship is an expression of Erickson's (1963) developmental stage of generativity, in which an older adult guides the next generation (Vance, 1982). Motivated by the needs of another, the concept of caring is at the core of the mentoring relationship. Although Vance applies mentorship principles to professional career development, this study found her mentorship theory to be extremely applicable to the issues of adolescent parenting. Vance (1995) explains that a mentor is someone who inspires, instructs, nurtures, and encourages the less experienced person. A mentor fulfils several roles including teacher, guide, advocate, and advisor. Within these roles, the functions of the mentor are to act as a role model and to provide education, guidance, nurturance, and support to the less experienced person'.<br>'Mentorship is described as a helping, relational phenomena with distinct characteristics. One such characteristic is that the mentoring relationship is intensive in nature, in contradistinction to helping relationships that are characterized by infrequent or sporadic interaction. The mentoring relationship is also characterized by emotional exchange; the less experienced person receives support and affirmation, and the mentor receives the satisfaction of having nurtured another human being while observing their developmental progress. Lastly, the mentoring relationship is by definition, terminal. The goal of mentorship is for the less experienced person to achieve a level of independence that no longer requires the guidance of the mentor. Vance proposes that mentorship results in a positive outcome for the less experienced person through increased perceived support.'<br><br>**Social support**<br>'Social support is defined by Kahn and Antonucci (1980) as an interpersonal transaction that includes the elements of (1) affect, such as respect; (2) affirmation, or approval; and (3) aid, such as tangible assistance that includes information. Importantly, Weiss (1974) theorizes that social

	support consists of six relational provisions. One of those provisions, guidance, is defined as advice and information usually obtained from mentors or parent figures. Therefore, based on Weiss' theory, mentorship—via guidance, information, and advice—is a provider of social support. With respect to the effects of social support, Mechanic and Cleary (1980) theorize that social support has a positive influence on health practices and that, conversely, social alienation has a negative effect on health practices.<br><br>'The positive effect of social support on health practices has been empirically supported by nursing research. Yarcheski and Mahon (1989) found that social support had a direct effect on health practices in a sample of 165 adolescents; in a refinement of their causal model investigating the effects of social support, age, self-esteem, gender, and future time perspective on health practices, other colleagues found that social support had the strongest effect on the positive health practices of adolescents and they concluded that perceived social support is a critical variable in the promotion of health behaviours among adolescents. Additionally, studies have shown that perceived social support among pregnant women is related to improved maternal health practices and positive perinatal outcomes. Lastly, guidance and information, identified by Weiss (1974) as a relational provision of social support, has specifically been found to positively influence maternal health practices among adolescent and low-income mothers. Programs providing maternal guidance and information during the first 5 to 24 months of the infant's life have demonstrated an improvement in maternal health practices, increased mother–infant interaction, and a reduction in infant mortality.<br><br>'Thus, a theorized outcome of mentorship (perceived support) has been demonstrated both theoretically and empirically to promote positive health practices among adolescents and pregnant women. Therefore, a mentorship framework stressing multiple aspects of support has utility in the development of a program designed to foster positive maternal health behaviors among adolescent mothers'.
Challenges & strategies	Only 74 participants completed the Child Abuse Potential Inventory (CAP) at Time 1. Only 19 of the participants completed the CAP at both Time 1 and Time 2 and had valid scores. The length of the instrument, which was 160 items and took approximately 1 hour to complete, was identified by participants and family support workers as the barrier to completion.
What is new?	This study suggests that an adolescent parenting programe based on the principles of mentorship and that provides intensive home visitation by nursing paraprofessionals who are indigenous to the community, may be effective in reducing infant mortality, low birthweight, and child maltreatment within a sample of high-risk, low-income, urban-residing adolescents. Further research that includes a large, geographically diverse sample, measures of social support and mentorship, and alternate measures of child abuse potential is recommended.
Additional references / Other comments	

**Quality Assessment – Flint 1999**	*Reviewer initials: CFT/LN*
Critical Appraisal	*The Mixed Methods Appraisal Tool (MMAT) 2018 will be used for quantitative, qualitative and mixed-methods studies.*

Screening questions

S1. Are there clear research questions? — Yes

Yes, to analyse the efficacy of a program designed to improve infant outcomes through the enhancement of health practices and parenting skills in a sample of low- income, pregnant and parenting adolescents who reside in an urban area and who screened positive for risk of child maltreatment.

S2. Do the collected data allow to address the research questions? — Yes

Yes, data was collected at baseline and parent and infant outcomes were captured and compared to local/national data.

MMAT for quantitative descriptive

1. Is the sampling strategy relevant to address the research question? — Yes

Yes, the sample included high-risk, pregnant and parenting adolescents over a 2-year study period.

2. Is the sample representative of the target population? — CT

The sample consisted of those 137 adolescents who had given birth, but can't tell if representative. Maternal age on admission ranged from 14 to 18 with a mean of 16.89 years (*SD* 4 1.12). Approximately 71% of the sample were African American, 27% were Hispanic, and 2% identified as other.

3. Are the measurements appropriate? — Yes

Yes, demographic data was obtained using the Healthy Families Baseline Data Survey. Outcome /process measures were obtained using a data collection tool designed for this project by the PI. Tools used Family Stress Checklist (FSC), Child Abuse Potential Inventory (CAP) (valid, reliable).

4. Is the risk of nonresponse bias low? — No

Among the 137 participants who gave birth, the % of neonatal mortality was 0 (prior to reaching 12 months of age, 29 infants were lost to follow up). However, only 74 participants completed the CAP at Time 1, only 19 at both Time 1 and Time 2 and had valid scores.

5. Is the statistical analysis appropriate to answer the research question? — CT

A descriptive study would not be the most appropriate design to test the efficacy of the programme (that would require a trial) but methods appropriate to assess the outcomes of the programme.

**Study ID: Havens 1997**	*Reviewer initials 1&2: CFT/AMK*

Reference:

Havens KK, Wagstaff DA, Mercer PA, Longeway K, Gutman M. Lessons learned from a mentoring program for teenage mothers. WMJ: Official Publication of the State Medical Society of Wisconsin. 1997 Sep 1;96(9):38-43.

Other publications from same study:

NA

Methods	**Study design**: randomised study/trial<br>**Primary aim**: to evaluate a mentoring program (Milwaukee Collaborative Teenage Pregnancy Prevention Program (MCTPP) designed to decrease the risk of repeat pregnancy among unmarried primiparous teens, ages 12-19.<br>**Intervention**: a mentoring programme that included social support and assistance dealing with community agencies from mentors who were trained community volunteers.<br>**Baseline measurements**: ethnicity, failure of a grade, last grade attended, grade point average, teen age (at first period, first intercourse, first use of birth control, first birth), mother age at fist birth, age of baby's father, desired age at second birth, sexual behaviors.<br>**Duration of study**: 1988-1990.
Participants	**Setting**: Milwaukee, Wisconsin, USA<br>**Inclusion criteria**: unmarried teens (ages 12-19) and in the third trimester of her first pregnancy. Recruited through an alternative school for pregnant teenagers (52, 46%), hospital's obstetrics clinic (40, 36%) ; and the remaining 18 (16%) teens were recruited through community health centres and private physicians.<br>**Exclusion criteria**: Not reported.<br>**Total number of participants**: 110 teens recruited and randomised: 53 mentor group; 57 control group.<br>**Mean age of participants (years) (SD):** (birth): mentor group: 16.6 ± 1.3; control group: 16.6 ± 1.4, 57. All mean age of enrolment 16.5 years.<br>**Mean gestational age at first visit (weeks)**: Not reported.<br>**Mean gestation age at time of start of intervention (weeks)**: Not reported.<br>**Ethnicity**:<br><u>White</u>: mentor group: 3 (5.7%); control group: 3 (5.3%)<br><u>Black</u>: mentor group: 47 (88.7%); control group: 53 (93%)<br><u>Hispanic</u>: mentor group: 2 (3.8%); control group: 0<br><u>Native American</u>: mentor group: 1 (1.9%); control group: 1 (1.8%)<br>**Marital status**: All unmarried.<br>**Socio-economic status**: 75% of teens were insured through Medicaid.<br>**Education**:<br><u>Failed a grade</u>: mentor group: 29 (54.7%); control group: 28 (49.1%)<br><u>Last grade attend</u>:<br>7th Grade: mentor group: 1 (1%); control group: 0<br>8th Grade: mentor group: 5 (9.4%); control group: 7 (12.3%)<br>9th Grade: mentor group: 13 (24.5%); control group: 14 (24.6%)<br>10th Grade: mentor group: 11 (20.8%); control group: 11 (19.3%)<br>11th Grade: mentor group: 12 (22.6%); control group: 14 (24.6%)<br>12th Grade: mentor group: 7 (13.2%); control group: 10 (17.5%)<br>GRADE Programme: mentor group: 3 (5.7%); control group: 1 (1.8%)<br>Missing: mentor group: 1 (1%); control group: 0<br>**Other lifestyle/health behaviors**:<br><u>Mean age for first intercourse</u>: 14.3 years.<br><u>First intercourse between the ages of 11 and 13</u>: 76%<br><u>Used birth control the first time they had intercourse</u>: 29%<br><u>Never used birth control prior to their first delivery</u>: 41%

Intervention	**Type and content of intervention**: The programme assumed that the mentor would serve as a role model, an information/education resource, and a source for social support for the teen and her family. MPPCT was a joint venture of a medical school, an urban hospital and a community-based minority operated social service agency.<br>Key roles: Initially, staffing of the mentor component consisted of the director of the Social Service agency and a volunteer coordinator, who was an experienced social worker responsible for the day to day running of the program and also recruited mentors, conducted the 6 weeks mentor training program and quarterly training meetings, and served as the mentor's mentor.<br><br>Components (activities, sessions, characteristics, or behaviours):<br>- Mentors were expected to spend at least 12h per month with their teens including telephone conversations or social outlings (i.e. movies, shopping, eating out, visiting each others home). During the initial relationships mentors were expected to build relationships with teens that fostered trust and friendship; and as the relationship developed, mentors were expected to devote more time on setting goals, spend time talking about contraception, continuing in school, day-care, parenting, budgeting, and also spend more time with the teens helping them to deal with problems.<br>- Mentors were paid 125USD for the 6 weeks training, 25USD for attending each quarterly meeting, plus 100USD for each month they spend 12-24h with their teens and 150USD if they spent more than 24hours. They could also claim back up to 50USD per month as reimbursement for theatre tickets, meals etc.<br>- By project's end, 57 women had served as mentors. The mean age was 35.9 + 8.7 years (n=52); the youngest mentor was 2 1 .5 and the oldest mentor was 57.3. The majority were African American working women. They had lived in Milwaukee for 18.3 + 11 .8 years (n=50). Thirty-seven mentors had children; 43% (16/37) had their first child when they were teenagers.<br><br>Recruitment of mentors: in the first year, recruitment using small media (eg bus signs, posters, broachers) and word of mouth. Mentors then recruited through word of mouth and personal contact. Each woman had to complete a written application and interview to assess her experience, availability, knowledge of the community resources, commitment to working with teen's family and attitudes of birth control.<br><br>Training and supervision of mentors: 6 weeks mentor training programme consisted of 20 hours of classroom work, and 5-10 hours of homework. The training emphasised mentor's role as a resource of support and resource for teens, her family and her siblings; identified available community resources and information on access; provided mentors with the knowledge they needed to discuss healthcare and contraception with their teens, and emphasised importance of commitment and confidentiality. Quarterly training meetings were used to enhance the mentor's basic training and to deal with any problems she was experiencing. These meetings also allowed mentors to receive support from other mentors, the Volunteer Coordinator, and the Social Worker.<br><br>Recruitment of girls: see details above in eligibility.<br><br>Matching: Teens were formally matched by the Volunteer Coordinator. The match was made after the Volunteer Coordinator (or Social Worker) met with the teen and her mother/family, and after the Volunteer Coordinator and Social Worker had observed the mentor in the training program. 58 (30) were successfully matched with one mentor who worked with her teen for the entire two-year period; the remaining teens (22) were matched with two or three mentors. (One teen declined further participation shortly after she was assigned to the mentor group.)<br><br>Duration of mentoring program: 12 months.<br><br>Follow ups: follow-up interviews conducted at 6, 12, 18, and 24 months postpartum. While the paper discusses repeat pregnancy outcomes at 33 months, and references Project Redirection's 5-

	year follow-up, its *own* empirical data collection extends to 24 months. The 5-year follow-up data is not from this study)<br><br>Safeguarding, support issues (e.g. clean criminal records): Training and support was seeing as a crucial component.<br><br>**Type and content of control (if applicable):** Not described.
Outcomes	**Outcome considered in the review and reported in the study:**<br>Repeated pregnancy at 33 months: 33 (66%) mentor group; 33 (68.%) control group<br><br>Number of Repeat Pregnancies:<br>0: 17 (34.0%) mentor group; 15 (31.2%) control group<br>1: 20 (40.0%) mentor group; 20 (41.7%) control group<br>2: 12 (24.0%) mentor group; 11 (22.0%) control group<br>4: 1 (2.0%) mentor group; 2 (4.2%) control group<br>Unknown: 3 mentor group; 9 control group<br><br>School advancement:<br>Graduated or Advanced 2 grades: 28 (52.8%) mentor group; 27 (47.4) control group<br>Delayed: 15 (28.3%) mentor group; 14 (24.6%) control group<br>Dropped out: 5 (9.4%) mentor group; 7 (12.3%) control group<br>Missing: 5 (9.4%) mentor group; 9 (15.8%) control group<br><br>The Piers-Harris Self-Concept Scale, the Nowicki-Strickland Locus of Control Scale, and the Beck Depression Inventory were administered to determine if pregnant teens suffer from low self-esteem or psychological distress that intensifies after they become parents – no sig differences.<br><br>36% 1 repeat pregnancy; ; 24% had two or more pregnancies<br>62% of the pregnancies with known resolution (89) resulted in live births; 26% abortion.
Underpinning theory of the intervention	The programme assumed that the mentor would serve as a role model, an information/education resource, and a source for social support for the teen and her family.
Challenges & strategies & lessons learnt	Due to relocation, they could not determine the status of three of the mentored teens and nine control teens; and because of the nature of our data, they could not determine if the two groups differed with respect to the timing or spacing of the various pregnancies. By 33 months, they knew of 98 repeat pregnancies; of the 89 pregnancies with known resolution, there were 55 live births, 23 abortions, 9 miscarriages, and 2 stillbirths (not given by groups). A total 60% repeat pregnancy rate was much larger than that typically reported (50% in Project Redirection)<br><br>The high percentage of teens advancing in school may reflect the fact that 52 teens were recruited from an alternative school that emphasized the importance of a high school diploma. It is also likely that the school's daycare center may have contributed to the high percentage of teens making progress in school. Still their 50% school completion/advancement rate may be lower than that reported in the literature.<br><br>Main reasons for findings:<br>1) Possibly the mentoring program may have truly had no impact on repeat pregnancy or educational outcomes - the domains targeted, but may be due to an inadequate theoretical model, faulty implementation, or both. Authors believed that the teens needed a role model whose struggles and achievements exemplified the belief that life offers options that are within one's grasp. Life options and mentoring programs are still sufficiently new such that formal program evaluations have yet to be reported in the literature. From the reports of both mentors and teens it was clear that some mentors

	experienced great difficulty maintaining contact with their teens, and that some teens moved frequently, were unable to maintain continuous telephone service, or simply did not wish to do things with the mentor. Thus, it is possible that the mentored teens did not receive sufficient mentoring. And thus it is possible difficulty mentors had discussing sexuality and contraception with their teens.<br><br>During their "exit" interviews, when asked to reflect upon their experiences, mentors most frequently recalled discussing with their teens the importance of education, school and relationship problems. In contrast, mentors seldom documented in their logs or problem lists any discussions of sexuality or contraception. Despite their training, mentors were unprepared to initiate personal conversations that most often occur in a recognized social context between the teen and her mother, the teen and her doctor (or minister), or the teen and her best friend. This role ambiguity may have contributed to the mentor and teen's inability to engage one another on such sensitive topics.<br><br>2) The mentoring program implemented may have had an impact; however, the effects may have been subtle or the effects may appear at a later date. The applicant interview data suggested that some women volunteered as mentors because someone from their community had taken an interest in them when they were young. Thus, it is possible that volunteerism at a future date may be one of the ways the mentor experience would affect the teens. It certainly impacted the mentors themselves. While they were mentors, 45% of all mentors reported returning or completing advanced educational degrees and/or upgrading their jobs.<br><br>3) It may be that the control group teens received support from their immediate families, relatives, boyfriends, and friends that was comparable to the support that mentored teens received from their mentor, and their immediate family, relatives, boyfriends, and friends. Underlying this explanation is the hypothesis that mentor and control group teen mothers experienced similar, basic needs which were met by the teens distributing the needs to the different resources for action.
What is new?	This study suggests that the mentoring scheme did not have any impact on repeat pregnancy, educational outcomes, and teen's psychological adjustment. Key points<br>1) the teen has to want to have a mentor. We chose to assign teens at random; however, mentored teens who did not want a mentor did not have to meet with her, and control teens who wanted a mentor found one.<br>2) we do not yet know how to provide mentors with the knowledge, language, and skills needed to talk with teens about sexuality and contraception. Mentors and teens did talk about school and they did talk about boyfriends and relationships. However, they seldom talked about sex, a behavior that is far more private and personal. In hindsight, our mentors had the difficult task of negotiating a role that does not yet exist in most social networks: as teens, few of us talked about intimate matters with an individual who was not our parent or grandparent, a friend, relative, physician, or teacher.<br>3) Individual, intensive mentor-teen bonds may be less effective in preventing undesirable outcomes than multiple, diffuse social bonds. In MCTPPP's predecessor, the teens participated weekly in a neighborhood support group and talked with other teens about contraception, their feelings about becoming pregnant and starting a family, and any problems that they were having with family members and friends. It was our hope that the mentor relationship in MCTPPP would allow similar discussions.<br><br>If applied research is to inform program design and social policy, applied researchers must learn from their efforts. As policy makers turn their attention to welfare reform and adolescent parenthood, we hope that they take a detailed look at the substance that underlies past and current pregnancy prevention efforts.

Additional references / Other comments	Baseline characteristics similar in terms sexual histories, school achievement profiles, age when they had a second child.

**Quality Assessment – Havens 1997**	*Reviewer initials: CFT/AMK*
Critical Appraisal	*The Mixed Methods Appraisal Tool (MMAT) 2018 will be used for quantitative, qualitative and mixed-methods studies.*<br><br>**Screening questions**<br>1. <u>Are there clear research questions?</u> — Yes<br><u>Yes,</u> to evaluate if a mentoring scheme decreases the risk of anticipated repeat pregnancy among participating unmarried primiparous teens and impact on school achievement too.<br><br>2. <u>Do the collected data allow to address the research questions?</u> — Yes<br>Yes, data was collected from interviews at baseline (during the third trimester) and additional interviews at 6, 1 2, 1 8, and 24 months postpartum. They provided self-report data on repeat pregnancy, educational outcomes, and the teen's psychological adjustment.<br><br>**MMAT for quantitative RCT**<br>1. <u>Is randomization appropriately performed?</u> — CT<br>A block randomization procedure was used to ensure that the two groups were comparable with respect to age at enrolment and recruitment source, but not details about how that block randomisation took place.<br><br>2. <u>Are the groups comparable at baseline?</u> — Yes<br>Yes they were both similar in terms of race, school performance (percentage enrolled, percentage ever failing a grade, last grade attended, cumulative grade point average), or sexual history (age at menarche, age at first intercourse, age at first use of birth control, age at first birth). Mentor and control teens also did not differ with respect to the age they wanted to be when they had a second child.<br><br>3. <u>Are there complete outcome data?</u> — Yes<br>87% completed the 6-month interview; 80% completed the 12-month interview; 77% completed the 18-month interview; and 74% completed the 24-month interview.<br><br>4<u>. Are outcome assessors blinded to the intervention provided?</u> — CT<br>Can't tell. Medical and psychosocial data were also obtained from teen's physicians and mentors.<br><br>5. <u>Did the participants adhere to the assigned intervention?</u> — CT<br>Can't tell. No measures of fidelity reported.

**Study ID: Hurd and Zimmerman 2010**	*Reviewers' initials: AKM/CFT*

Reference:

Hurd and Zimmerman. Natural Mentoring Relationships among Adolescent Mothers: A Study of Resilience 2010 Jun;16(3):182-9.

Other publications from same study: N/A

Methods	**Study design**: Quantitative (longitudinal).<br>**Primary aim**: to assess potential natural mentoring long term effects on adolescent mothers' wellbeing as they transitioned from adolescence into adulthood.<br>**Intervention**: Not applicable (longitudinal study)<br>**Baseline measurements**: ethnicity, marital status, socio-economic status, pregnancy/postpartum stage.<br>**Duration of study**: Not reported. Study published in 2010
Participants	**Setting**: Michigan, USA<br>**Inclusion criteria**: a) African American adolescent mothers who were pregnant and/or parenting during their senior year of high school thus likely 17-18 years old (or what would have been their senior year for those who dropped out of school), b) an eighth-grade GPA of 3.0 or lower and the absence of an emotional or developmental disability.<br>**Total number of participants**: 93*<br>Mean age of participants (years) (SD): Average participant age during the 12th grade year was 17.66 (SD = .65)<br>Mean gestational age at first visit (weeks): Not reported.<br>Mean gestation age at time of intervention (weeks): Not reported.<br>**Ethnicity**: African American<br>**Marital status**: Single Mothers**<br>**Socio-economic status**: Poor Socioeconomic Status (disadvantaged background).<br>**Education**: High School Students<br>**Other lifestyle/health behaviors**:<br><u>Pregnant with first child</u>: 23<br><u>Had one child</u>: 57<br><u>Had two children by their senior year of high school</u>: 13<br>**The final analyses included all African American adolescent mothers with at least two waves of data (n = 93).*<br>**Majority of the participants were living with their parents and only 15% of participants reported being married or living with a partner at any time during the study.*
Intervention	**Type and content of intervention:** Natural mentoring.<br><br><u>Components (activities, sessions, characteristics, or behaviours)</u>: Not reported. Natural mentoring.<br><br><u>Recruitment of mentors</u>: Not applicable. Participants were asked, "Is there an adult 25 years or older who you consider to be your mentor? That is, someone you can go to for support and guidance, or if you need to make an important decision, or who inspires you to do your best?" If participants responded in the affirmative, they were asked, "What is his/her relationship to you?" If participants identified a parent or step-parent as their mentor, they were asked the first question again, but asked to identify someone other than a parent or person who raised them. Participants who identified a mentor who was not a parent, step-parent, or person who raised them qualified as having a natural mentor. This item was used to create a dichotomous natural mentor variable (0 = no mentor, 1 = mentor).

	Training and supervision of mentors: Not reported (natural mentors)<br><br>Recruitment of girls: Not reported.<br><br>Matching: Attempts were made to match interviewers by race and gender.<br><br>Duration of mentoring program: Not reported.<br><br>Follow ups: Participants were interviewed each year of high school and four times across the five years following high school.<br><br>Safeguarding, support issues (e.g. clean criminal records): Not reported.<br>No many details – Adolescent girls who met the inclusion criteria were invited to complete structured interviews and self-report questionnaires (paper- and-pencil format) after the interview to collect info about participants' drug and alcohol use. Participants who were enrolled in school in the 9th through 12th grade years were called from their classrooms and interviewed at school. Participants who were not enrolled in school were contacted and interviewed at home or at a location specified by the participant. Data collection in the years following high school completion involved contacting participants and interviewing them at home or at a specified location in the community.
Outcomes	**Outcome considered in the review and reported in the study:**<br><br>Multiple scales and models to measure: depressive symptoms, anxiety symptoms, stress, parental support, presence of natural mentor and demographics.<br><br>Participants reported having a natural mentor: 57/ 93 participants (61%). The natural mentors identified were primarily female extended family members (i.e., grandmothers, aunts, and cousins) and older siblings. Other roles included Godparent, parent's friend, neighbour, and minister.<br><br>Having a natu ral mentor moderated the relationship between stress and depressive symptoms as well as the relationship between stress and anxiety symptoms among African American adolescent mothers. Thus, natural mentors may have helped youth be resilient in the face of stress (e.g. effective coping strategies, emotional support, material aid or physical assistance/support (e.g., assist with childcare) that may help to buffer against the negative outcomes associated with stress.<br><br>Participants with natural mentors demonstrated less symptoms of depression and anxiety over time.
Underpinning theory of the intervention	Evolving evidence suggest that resilience theory may provide a useful approach for studying long term outcomes among adolescent mothers because a resilience model focuses on factors that promote successful adjustment despite adversity.<br><br>Resilience theory helps explain why some youth who experience adversity are able to thrive in the face of risk. The resilience process refers to positive adjustment among youth who have been exposed to one or more risk factor(s). Risk factors increase the likelihood of developing negative outcomes. Promotive factors, on the other hand, contribute positively to youth outcomes (i.e., compensatory factors) and/or buffer youth from negative outcomes associated with risks (i.e., protective factors). Promotive factors may be individual assets (e.g., self-efficacy) or resources from an individual's environment (e.g., mentors).<br><br>Resilience theory is a useful approach for estimating outcomes among at-risk populations because it allows researchers to focus on factors that may predict positive development within these populations. Thus, a resilience approach is unique because it focuses on

	strengths within the individual and the individual's environment as opposed to solely focusing on deficits and blaming at-risk populations for their own problems. Few researchers have used a resilience approach to discover specific factors that may counter or protect adolescent mothers from the negative outcomes associated with the risks they face. Of the few researchers who have investigated promotive factors for this population, some have found that the presence of a strong supportive relationship may contribute significantly to resilience among African American adolescent mothers.<br><br>Authors hypothesized that adolescent mothers who reported having natural mentors during their 12th grade year (or what would have been their 12th grade year for those who dropped out of school) would demonstrate greater decreases in symptoms of depression and anxiety over time. They also hypothesized a weaker relationship between stress and mental health problems over time among participants with natural mentors.
Challenges & strategies & lessons learn	Findings proved that by effective coping strategies, natural mentors may help adolescent mothers cope more effectively with stress and experience fewer symptoms of depression and anxiety. Also, natural mentors may provide emotional support to adolescent mothers and thus, provide adolescent mothers with a safe outlet for expressing their emotions and requesting guidance. In addition, natural mentors may be contributing independently to healthier psychological outcomes among adolescent mothers. Through their interest in and commitment to adolescent mothers, natural mentors may cultivate a more positive self-appraisal among adolescent mothers and add to young mothers' perceptions of self-worth. By promoting healthier self-images among adolescent mothers, natural mentors may be reducing adolescent mothers' vulnerabilities to mental health problems – consistent with findings from previous results regarding the potential promotive effects of natural mentors on adolescent mothers' psychological well-being.<br><br>Several limitations: 1) participants were only asked about the presence of a natural mentor during their senior year of high school. Thus, we do not know when these relationships began or if they persisted beyond participants' senior year of high school; 2) no assessment of individual characteristics that may have both contributed to participants' ability to form relationships with natural mentors and contributed to more positive mental health outcomes, 3) no data collected on support received from fathers of adolescent mothers' children or from adolescent mothers' partners, 4) small sample size, but yet, authors found theoretically consistent findings, suggesting that the effects of natural mentors may be robust even after controlling for several other variables.
**What is new?**	This is one of the first studies to examine this relationship longitudinally among a group of African American adolescent mothers transitioning into adulthood. The results suggest that encouraging the formation of natural mentoring relationships may be an effective strategy for promoting resilience among African American adolescent mothers. Adolescent mothers could benefit from learning how to identify supportive nonparental adults in their lives and how to cultivate relationships with these adults. Likewise, informing extended family members and adults that work with adolescent mothers of the potential positive effects associated with natural mentoring relationships may help motivate these adults to take advantage of mentoring opportunities.<br><br>Creating environments where adolescent mothers and familiar adults can form mentoring relationships naturally may lead to more influential and enduring mentoring relationships, particularly in comparison to mentoring relationships wherein adults and youth are paired through formal programs. Several researchers have suggested that some formal mentoring relationships may be more vulnerable to early termination due to mismatches between mentees and mentors (a lack of chemistry), poor relationship quality, and a lack of commitment and follow-through by mentors and mentees. Whereas, allowing mentoring relationships to form naturally in a supportive environment may protect these relationships from some of the early-termination risks faced by some formal mentoring relationships.

**Additional references / Other comments**	

**Quality Assessment – Hurd and Zimmerman 2010**	*Reviewer initials: AMK/CFT*
Critical Appraisal	*The Mixed Methods Appraisal Tool (MMAT) 2018 will be used for quantitative, qualitative and mixed-methods studies.*<br><br>**Screening questions**<br>1. Are there clear research questions? — Yes<br>Yes, to assess potential natural mentoring effects on adolescent mothers' wellbeing as they transitioned from adolescence into adulthood.<br><br>2. Do the collected data allow to address the research questions? — Yes<br>Yes, questionnaires were administered to adolescent after 5 years.<br><br>**MMAT for quantitative descriptive**<br>1. Is the sampling strategy relevant to address the research question? — Yes<br>Yes, sampling strategy (schools, communities/homes) was appropriate to identify African American adolescent mothers who were pregnant and/or parenting during their senior year of high school, but also those who were not attending school.<br><br>2. Is the sample representative of the target population? — Yes<br>Yes, it looks so – plus no difference between participants who were pregnant, and those parenting on socio economic status.<br><br>3. Are the measurements appropriate? — Yes<br>Used valid, reliable scales/questionnaires to measure depressive/anxiety symptoms, stress and support.<br><br>4. Is the risk of nonresponse bias low? — Yes<br>Yes, this study included only all African American adolescent mothers with at least two waves of data completed.<br><br>5. Is the statistical analysis appropriate to answer the research question? — CT<br>Probably yes, limitations acknowledged. Not really known what the mentoring relationship included at all, duration, content etc.

**Study ID: Klaw 2003**	*Reviewers initials: CFT/MK*

Reference:

Klaw EL, Rhodes JE, Fitzgerald LF. Natural mentors in the lives of African American adolescent mothers: Tracking relationships over time. Journal of youth and adolescence. 2003 Jun;32:223-32.

Other publications from same study:

*Klaw EL, Rhodes JE. Mentor relationships and the career development of pregnant and parenting African American teenagers. Psychology of Women Quarterly. 1995 Dec;19(4):551-62.

*Authors contacted, unclear if different study population/sample so data extracted independently to Klaw 1995

Methods	**Study design**: Quantitative (interview administered survey)<br>**Primary aim**: to explore the academic attainment of African American adolescents as they made the transition from pregnancy or recent delivery to 2 years postpartum<br>**Intervention**: Natural mentoring<br>**Baseline measurements**: age, marital status, number of children, and living arrangements.<br>**Duration of study**: Unclear. Study published in 2003.
Participants	**Setting**: an alternative school for pregnant and parenting students, located in a large midwestern city, US<br>**Inclusion criteria**: African-American adolescents between the ages of 11and 19 year who were enrolled in the school.<br>**Total number of participants**: 198*<br>**Mean age of participants (years) (SD):** 15.94, (1.40)<br>**Mean gestational age at first visit (weeks)**: Not reported.<br>**Mean gestation age at time of intervention (weeks)**: Not reported<br>**Ethnicity**: African American<br>**Marital status**: All: unmarried.<br>**Socio-economic status**: More than half (64%) were receiving public assistance benefits,<br>**Education**: Not reported.<br>**Other lifestyle/health behaviors**: 73.2%: pregnant; 26.8%: had recently given birth to their first child at the time of the first interview.<br>5% of the participants had 2 or more children.<br><br>*The study categorized adolescent mothers into groups based on the presence and stability of natural mentor relationships over a two-year period. Of the total sample, 51.5% had a mentor at Time 1. At follow-up, 48.5% still had the same mentor (long-term mentor group), while 18.2% no longer did (terminated mentor group). Another 26.8% never had a mentor (never mentor group), and some participants (about 27%) were excluded due to missing data or having a different mentor at follow-up. The analysis focused on comparing outcomes between those with long-term mentors and those with no mentors during the postpartum period to understand the impact of sustained mentoring relationships.
Intervention	**Type and content of intervention**: Natural mentorships<br>Components (activities, sessions, characteristics, or behaviours):<br>As part of the standardized interview process, participants were first asked, "Other than your parents or whoever raised you, do you have a role model or mentor who you go to for support and guidance? A mentor is not someone around your age or a boyfriend. He or she is an adult who is older than you, who has had more experience than you, and who has taken a special interest in you. This person may be a teacher, a relative, a neighbour, or someone else whom you look up to for support and guidance.<br>Four characteristics of the relationship were then listed as criteria for persons to be nominated as mentors: (1) you can count on this person to be there for you, (2) he or she believes in and cares deeply about you, (3) he or she inspires you to do your best, and (4) knowing him or her has really affected what you do and the choices you make. The verbal nature of the interviews allowed the

	interviewer to clarify that the definition of mentor did not include peers or romantic partners, but nonparental relatives could serve as mentors. Care was taken to ensure that each student understood the criteria for mentoring.<br>The participants were then categorized into one of several relationship groups. The "long-term mentor" group included participants who nominated the same mentor at Time 2 as they had nominated at Time 1 (*N*=36,18.18%). The "terminated mentor" group included participants who nominated a mentor at Time 1, but not at Time 2 (*N*=36, 18.18%). The "no mentor" group included participants who had not nominated a mentor at Time 1 or at Time 2 (N=53, 26.77%).<br><br><u>Recruitment of mentors</u>: NA.<br><br><u>Training and supervision of mentors</u>: NA.<br><br><u>Recruitment of girls</u>: All pregnant and parenting adolescents' part of the alternative school invited to participate in the study.<br><br><u>Matching</u>: NA<br><br><u>Duration of mentoring program</u>: 2 years.<br><br><u>Follow ups</u>: 1 year and 2 year (this study just focused on 2 year follow up)<br><br><u>Safeguarding and support issues</u> (e.g. clean criminal records): Informed consent of the students and parents was obtained, and participants received $10 vouchers. Interviews were conducted by a trained, female, African American research associate, keeping confidentiality.<br><br>**Type and content of control (if applicable):** NA
Outcomes	**Outcome considered in the review and reported in the study:**<br>School records were used to assess each participant's school enrolment status subsequent to leaving the alternative school (dropped out versus enrolled/graduated) and highest grade levels attained. In addition, participants were asked to report their level of education attained at Times 1 and 2.<br><br><u>Differences in educational attainment as function of relationship duration:</u> educational attainment differed based on the duration of mentor relationships. At the two-year follow-up, participants with long-term mentors were significantly less likely to have dropped out of school compared to those with no mentors, only 35% in the long-term mentor group had dropped out, versus 64% in the no mentor group. Logistic regression showed that having a long-term mentor reduced the odds of dropping out by more than three times. Maternal support did not significantly impact school dropout rates, highlighting the unique role of sustained mentoring in promoting educational persistence among adolescent mothers.<br><br><u>Long term relationships:</u> Long-term mentors were more likely to be relatives compared to mentors whose relationships ended. Half of the participants viewed their mentors as parental figures, while others saw them as older siblings or other caregivers. These long-term mentor relationships were highly significant, with most participants interacting weekly or even daily. On average, the mentoring relationships lasted about 14 years, and most participants expected them to continue lifelong. Mentors supported the young women in many ways, including helping them stay in school, offering personal advice, lending items, teaching employment skills, and providing opportunities for fun and relaxation.<br><br><u>Long term mentor vs maternal support</u>: The study compared support from adolescents' long-term mentors and their mothers over time. Results showed no difference in the total amount of maternal support across groups or over time. However, satisfaction with maternal support was higher in the group with terminated mentors compared to those with long-term or no mentors.

	When comparing mentors and mothers directly, both provided less support at the later time point, but mentors consistently offered more emotional support, socializing support, and guidance than mothers. Participants rated mentor support as more important and were more satisfied with mentor support than maternal support at both times measured. Overall, long-term mentors provided a uniquely valuable and appreciated source of support compared to mothers.
Underpinning theory of the intervention	Natural mentoring relationships act as a protective factor that can improve life outcome, particularly educational attainment, for African American adolescent mothers. The authors hypothesize that enduring, supportive relationships with nonparental adults provide emotional, informational, and instrumental support that is often lacking or diminished in maternal relationships during the postpartum period. These mentors, typically older women within the adolescent's social network, can help young mothers navigate the complex transition to parenthood by modelling positive behaviours, reinforcing the value of education, and offering practical help such as childcare and career guidance. Through these mechanisms, mentors are thought to foster resilience, increase motivation, and reduce the likelihood of school dropout, ultimately promoting long-term educational and developmental success.
Challenges & strategies, lessons learn.	African American adolescent mothers often face a confluence of challenges, including early parenthood, economic hardship, racial oppression, and diminished educational opportunities. These difficulties frequently contribute to school dropout and limited career prospects. This study revealed that support from natural mentors, nonparental adults such as aunts, grandmothers, and older siblings, can help buffer against these adversities. However, not all mentoring relationships are equally effective. The greatest educational benefits were observed among young mothers who maintained the same mentor relationship over a two-year postpartum period. These enduring connections were typically characterized by frequent, emotionally supportive interactions and were often rooted in long-standing familial or community ties.<br><br>To address these challenges, the researchers emphasized the importance of sustained, high-quality mentoring relationships. Strategies that proved effective included fostering consistent and emotionally rich mentor engagement, emphasizing educational encouragement, and supporting adolescent mothers with tangible assistance such as childcare and guidance on employment. A key lesson learned was that enduring mentorships were more impactful than maternal support in promoting educational attainment. Young mothers in long-term mentoring relationships were significantly more likely to stay in school or graduate, and they reported higher satisfaction and perceived importance of mentor support compared to maternal support. These findings underscore the critical role of consistent adult allies in promoting resilience and positive outcomes among at-risk youth.
What is new? (conclusions)	Authors found that enduring relationships with natural mentors significantly enhance educational outcomes for African American adolescent mothers during the challenging two years postpartum. These mentor, often female relatives, provided emotional support, guidance, and encouragement that participants found more impactful and satisfying than maternal support. Long-term mentor relationships were associated with a higher likelihood of staying in school or graduating, suggesting that consistent, nonparental adult support plays a critical compensatory role when maternal support is limited. Overall, the findings highlight the importance of fostering stable, supportive adult relationships and suggest that structured mentoring interventions could help replicate the benefits of natural mentorship for those without access to such relationships.
Additional references / Other comments	Participants with mentors at Time 1 were approximately 6 months younger than those without mentors ($t(343)=-3.65$, $p < 0.01$), but there were no other mentor versus no-mentor differences

**Quality Assessment – Klaw 2003**	*Reviewer initials: CFT/MK*
Critical Appraisal	*The Mixed Methods Appraisal Tool (MMAT) 2018 will be used for quantitative, qualitative and mixed-methods studies.*<br><br>**Screening questions**<br>1. Are there clear research questions?<br>The study clearly asks how the presence and duration of natural mentoring relationships affect educational attainment in African American adolescent mothers<br><br>2. Do the collected data allow to address the research questions?<br>*Yes* – Data on mentoring relationships, maternal support, and educational outcomes were collected and analysed over two years to address the research objectives.<br><br>**MMAT for quantitative descriptive**<br>1. Is the sampling strategy relevant to address the research question? Yes<br>Yes, all girls attending an alternative for pregnant and parenting girls were invited.<br><br>2. Is the sample representative of the target population?<br>Probably yes, sample includes African American adolescent mothers in one Midwestern city and attending a specific school, limiting generalizability. However, it is appropriate for understanding this subpopulation. Yes<br><br>3. Are the measurements appropriate? Yes<br>*Yes*, validated tools such as the Social Support Network Questionnaire (SSNQ) and school records were used. Mentor support was measured using clear criteria consistent with existing literature.<br><br>4. Is the risk of nonresponse bias low? No<br>No, there was a substantial amount of missing data and attrition over the two-year follow-up, with some participants excluded due to incomplete data or changing mentors<br><br>5. Is the statistical analysis appropriate to answer the research question? Yes<br>Yes, Chi-square analyses, t-tests, ANOVAs, and logistic regression were used to examine group differences and associations with educational outcomes.

**Study ID: Klaw and Rhodes 1995**	*Reviewers initials: CFT/MK*

Reference:

Klaw EL, Rhodes JE. Mentor relationships and the career development of pregnant and parenting African American teenagers. Psychology of Women Quarterly. 1995 Dec;19(4):551-62.

Other publications from same study:

Methods	**Study design**: Quantitative (interview administered survey)<br>**Primary aim**: to explore the extent to which natural mentors were associated with positive educational and career outcomes in pregnant and parenting African-American adolescent<br>**Intervention**: Natural mentoring<br>**Baseline measurements**: age, parenting/pregnant previous children, marital status, benefits<br>**Duration of study**: Unclear. Study published in 1995.
Participants	**Setting**: an alternative school for pregnant and parenting students, located in a large midwestern city, US<br>**Inclusion criteria**: African-American adolescents between the ages of 11and19 year who were enrolled in the school during the **1992-1993** academic year.<br>**Total number of participants**: 204<br>**Mean age of participants (years) (SD):** 15.9, (1.5)<br>**Mean gestational age at first visit (weeks)**: Not reported.<br>**Mean gestation age at time of intervention (weeks)**: Not reported<br>**Ethnicity**: African American<br>**Marital status**: All: never married.<br>**Socio-economic status**: More than half (66.2%) were directly receiving welfare benefits.<br>**Education**: Not reported.<br>**Other lifestyle/health behaviors**: 61%: expecting first child; 34%: had one child; 5%: had two or more children<br>*More than half of the participants nominated adults they considered to be mentors.
Intervention	**Type and content of intervention**: Natural mentorships<br><u>Components (activities, sessions, characteristics, or behaviours)</u>:<br>The literature on mentoring served as a basis for our definition of mentoring relationships and the conceptualization of mentor support in the sample. Participants were first asked, “Other than your parents or whoever raised you, do you have a role model or mentor who you go to for support and guidance. A mentor is not someone around your age or a boyfriend. He or she is an adult who is older than you, who has had more experience than you, and who has taken a special interest in you.” Several characteristics of the mentor relationship were then listed, including: “(1) that you could count on this person to be there for you, (2) that he or she believes in and cares deeply about you, (3) that he or she inspires you to do your best, and (4) that knowing him or her has really affected what you do and the choices you make, (5) that he or she is a model for the kind of person you would like to be, and (6) that he or she is a model for the kind of career successes you would like to have.<br><br><u>Recruitment of mentors</u>: NA.<br><br><u>Training and supervision of mentors</u>: NA<br><br><u>Recruitment of girls</u>: NA<br><br><u>Matching</u>: NA<br><br><u>Duration of mentoring program</u>: Not reported.

	<u>Follow ups</u>: Not reported.<br><br><u>Ethics, safeguarding and support issues</u> (e.g. clean criminal records): Not reported. Students who were contacted agreed to participate in the study, and the informed consent of the participants and their parents was obtained.<br><br>**Type and content of control (if applicable): NA**
Outcomes	**Outcome considered in the review and reported in the study:**<br>Mentor support, aspiration and expectation gap, mentor relationships, career related activities, opportunity structure benefits, life optimism.<br><br>• Relationship to participant: 118 (57.8%): nominated adults whom they considered to be mentors. 46.3% of the young women had known their mentors for at least 15years and more than 80% expected to maintain the relationship forever. 32% nominated aunts and 25.7% nominated grandmothers. Mentors also included older relatives, godmothers, teachers, counsellors, and boyfriends 'relatives.<br>• Frequency of interaction: nearly half (47.7%) of the participants reported that they saw their mentors daily, and an additional 48 % reported that they saw their mentors at least once a week.<br>• Proximity to participant: 46.8" same neighbourhood,46.8: which an hour away; 1% more than an hour away.<br>• Mentor support was significantly associated with participants' involvement in activities related to their career goals, more positive beliefs about the opportunity structure, and heightened optimism.<br>• Mentor support was not associated with diminished discrepancies between participants aspirations and their expectations. In fact, 66% (135) reported expectations that were equal to their aspirations. Only 27% (56) reported occupational expectations that were lower than their aspirations. The majority of participants envisioned professional careers, many of which were related to health or social service provision.<br>• When using path analysis to examine associations between mentor support and participants' career activities, beliefs about the opportunity structure, and aspiration-expectations, as well as associations between these variables, mentor support, and life optimism, authors found that, with one exception (mentor support to the aspiration-expectation gap) all of the paths were significant.
Underpinning theory of the intervention	Natural mentoring relationships are generally characterized as powerful, supportive emotional ties between older and younger persons in which the older member is trusted, loving, and experienced in the guidance of others. Unlike assigned mentor relationships, which originate through programs such as Big Brothers, Big Sisters, natural mentor relationships tend to emerge from within the youth's natural support network. Adolescents who have adjusted well despite profound, ongoing stress often attribute their success to the influence of a natural mentor, such as a special aunt, grandparent, or teacher (mentors' protective qualities are corroborated by the literature on resilience).<br><br>Natural mentor relationships appear to be extremely important to young African American mothers, who may be particularly receptive to nonparent adult support and guidance; as they rely on nonparent adults, adolescent mothers can gain some autonomy while simultaneously obtaining much needed emotional support and advice. Girls who identified natural mentors derived more benefits from their social networks and reported lower levels of depression than those who did not, despite comparable levels of support, stress, and economic strain. In addition to promoting psychosocial functioning, natural mentors might also facilitate young mothers' school achievement and involvement in career-related activities. It is possible that mentors help

	their proteges to define their "Dream," or vision of the future, and instill a sense of optimism about actually realizing this future dream.<br><br>Protective effects may depend on the nature and characteristics of the relationship, as they appear to differ greatly in terms of their intensity, content, and activities. Some mentors and proteges focus on issues related to academic and career development, others are more oriented toward interpersonal problems.
Challenges & strategies, lessons learn.	Mentor support was not associated with diminished discrepancies between the young women's career aspirations and their expectations of the future. This may have been caused, in part, by the limited variance in participants' responses or a sense of a "foreshortened future" among many inner-city youth. Also, Given the exclusive reliance on cross-sectional, self-report measures, our results must be interpreted with caution, and larger longitudinal studies needed (opportunity structure and life optimism scales had low internal consistency in this study too). Future research should look at the ways in which young women at various developmental stages construct their future goals, refine the operational definition of natural mentor.<br><br>Important implications for programs that pair volunteer mentors with at-risk adolescents - Like natural mentors, adults serving in this capacity might be able to offer inner-city youth some pro-taction against the many stressors in their lives. Similarly, pregnant and parenting adolescents might be taught techniques for recruiting the support of natural mentors in their own social networks. Encouraging adolescents to reach out to supportive adults, while **also** providing such adults with the time and incentives to informally interact with youth, might go a long way toward facilitating intergenerational contact.
What is new? (conclusions)	Natural mentors are an important protective resource for pregnant and parenting, African-American adolescents. In addition to promoting emotional well-being, natural mentor support may influence young mothers' educational and career development. A path model suggested that the support of mentors was positively related to participants' increased life optimism as well as their participation in career-related activities and beliefs that education would lead to future jobs. Furthermore, participation in career-related activities and the belief that education would lead to opportunities were each positively related to increased optimism. Taken together, the model suggests that mentor support may increase life optimism, both directly and through its influence on participants' career-related activities and beliefs in education as a link to opportunity.<br><br>A path model indicated that mentor support was associated with increased life optimism, beyond its indirect effects on career activities and beliefs about the opportunity structure. These findings suggest that natural mentors are an important resource in the career development of pregnant and parenting African American adolescents.
Additional references / Other comments	

**Quality Assessment – Klaw and Rhodes 1995**	*Reviewer initials: CFT/MK*
Critical Appraisal	*The Mixed Methods Appraisal Tool (MMAT) 2018 will be used for quantitative, qualitative and mixed-methods studies.*<br><br>**Screening questions**<br>1. Are there clear research questions? — Yes<br>Yes, to explore the extent to which natural mentors were associated with positive educational and career outcomes in pregnant and parenting African American adolescent<br><br>2. Do the collected data allow to address the research questions? — Yes<br>Yes, interview administered questionnaires<br><br>**MMAT for quantitative descriptive**<br>1. Is the sampling strategy relevant to address the research question? — Yes<br>Yes, all girls attending an alternative for pregnant and parenting girls on the 1993 academic were invited.<br><br>2. Is the sample representative of the target population? — Yes<br>Yes, it looks representative looking at the narrative of the findings.<br><br>3. Are the measurements appropriate? — Yes<br>Yes, piloted and tested many of the measures used – although a couple had relatively low reliability and acknowledged as a limitation.<br><br>4. Is the risk of nonresponse bias low? — CT<br>A total of 218 girls completed the interviews but unknown how many were initially approached.<br><br>5. Is the statistical analysis appropriate to answer the research question? — Yes<br>Yes, looking appropriate to measure associations and acknowledging limitations of cohort study design.

**Study ID: Lin 2019**	*Reviewers initials: CFT/AMK*

Reference:

Lin CJ, Nowalk MP, Ncube CN, Aaraj YA, Warshel M, South-Paul JE. Long-term outcomes for teen mothers who participated in a mentoring program to prevent repeat teen pregnancy. Journal of the National Medical Association. 2019 Jun 1;111(3):296-301.

Other publications from same study:

Methods	**Study design**: quantitative survey<br>**Primary aim**: to examine educational attainment, employment, and pregnancies of these adolescent mothers 1-5 years post program.<br>**Intervention**: Maikuru mentoring program<br>**Baseline measurements**: age at enrolment, age at birth of their baby, race, current educational level, educational and employment goals.<br>**Duration of study**: 2011-2015
Participants	**Setting**: Pittsburgh, USA<br>**Inclusion criteria**: girls: being the mother of a single child, under 20 years old and not currently pregnant.<br>**Total number of participants**: 74 teen mothers (23 dropped out, 51 completed the programme, 19, 37.3% successfully reached for follow up)<br>**Mean age of participants (years) (SD) (birth):** 16.7 (1.20) respondents; 16.8 (1.36) non respondents.<br>**Mean gestational age at first visit (weeks)**: Not reported.<br>**Mean gestation age at time of intervention (weeks)**: Not reported.<br>**Ethnicity** (Programe):<br>Of the 51 included: 94% African American/biracial, 6% were Caucasian/other<br>Of the 23 dropouts: 83% were African American/ biracial and 17% were Caucasian/other.<br><u>Ethnicity (Follow up survey):</u><br>African American race: 19 (100%) respondents; 29 (32%) non respondents<br>**Marital status**: Not reported.<br>**Socio-economic status**: Not reported.<br>**Education**:<br><u>Education during the program</u>:<br>Attending high school: 15 (78.9) respondents; 18 (56.3) non respondents<br>High school drop-out/working on GED: 2 (10.5) respondents; 6 (18.7) non respondents.<br>High school graduate/GED completed: 1 (5.3) respondent; 4 (12.5) non respondents.<br>Some post high school education: 1 (5.3) respondent; 3 (9.4) non respondents.<br>Other: 0 (0.0) respondent; 1 (3.1) non respondents.<br>**Other lifestyle/health behaviors**: Not reported
Intervention	**Type and content of intervention**: The Maikuru Program was an adult-teen mother mentoring program that 1) stressed delaying subsequent pregnancies while still in one's teens by discussing birth control op- tions; 2) offered support for staying in school, and 3) encouraged aspirations to higher education or employment in order to secure financial independence and enhance quality of life for themselves and their children.<br><br><u>Recruitment</u>: Teen mothers and adult Maikurus were recruited using a wide range of strategies including bus ads, flyers, word of mouth, referrals from schools, health centers treating teen mothers, social service organizations serving teens, and online advertising (special focus in poor neighbourhoods). Maikurus: being a mature adult at least 15 years older than the teen mother who demonstrated stability and willingness to work with a teen mother and who received

	appropriate legal clearances (Pennsylvania Act 33/34). Race was not a factor in determining eligibility.<br><br><u>Matching</u>: After signed consent, participants were interviewed and completed a set of surveys, one of which assessed their hobbies and interests. This survey was the basis of the Maikuru-teen matching process.<br><br><u>Training</u>: Maikurus were required to attend a one-day mentoring training session that was facilitated by an experienced leader from a local mentoring agency.<br><br><u>Components</u>: The program required that teen mothers attend eight weekly group sessions and subsequent follow-up sessions every three months, accompanied by their Maikurus. Each meeting offered a presentation and/or guided discussion led by a professional facilitator about topics such as birth control, personal finances, relationships with the child's father, etc. The investigators, research staff, professional presenters and the Maikurus were primarily African American.<br><br>The Maikuru served as an adult advisor and support system regarding child-rearing, relationships between the teen mother and her own family, as well as her child's father, school, employment or simply navigating adolescence. The Maikurus and teens could be in contact at their discretion, but the program team encouraged them to have a conversation at least once a month (either by phone or in person) and then keep contact through emails or text messages in between those meetings. Maikurus were discouraged from lending money or providing transportation to the group meetings. Support was offered to the Maikurus through Maikuru mentor sessions that were held every 4e6 weeks to allow Mai- kurus to share concerns among themselves and share potential solutions.<br>Following active intervention, the teens and their Maikurus were encouraged to stay in contact via phone, email, text, or Facebook. The project team formed a monthly continuation group to enable participants to gather at the meeting site to facilitate their continued interaction. Enrollment was rolling, that is, as soon as 6e8 teen mothers had consented to participate and were matched with Maikurus, a group was formed and inter- vention began, with groups meeting sequentially in 2010 and continuing through 2015.<br><br>Program follow- up meetings and surveys at 6, 12, 18 and 24 months (key follow-up for this specific paper's outcomes was the 1-5 years post program survey conducted in 2016.<br><br>**Type and content of control (if applicable): NA**
Outcomes	**Outcome considered in the review and reported in the study:**<br><u>Educational attainment at 1-5 years follow-up</u><br>Attending high school: 5 (26.3)<br>High school graduate/GED completed. 5 (26.3)<br>Some post high school education: 8 (42.1)<br>College graduate: 1 (5.3)<br><br><u>Employed at follow-up</u>: 12 (63.2)<br><u>No subsequent pregnancies</u>: 10 (52.6)<br><20 years old subsequent pregnancy: 2 (10.5)<br>=and> 20 years old subsequent pregnancy: 5 (26.3)<br>Unknown age subsequent pregnancy: 2 (10.5)<br><br><u>Satisfaction</u>:<br>Participants were asked about their perceptions of the program. Most of the comments we received were positive about its benefits. Some examples were, "It helped teach me a lot about being a good mom. I want to open up an orphanage with my business degree." "Wouldn't have the support, guidance, and resources without the program." "There was a lot of support and

	information to help you with being a mom because young moms always feel bad." "Thankful for the program to help me stay focused."
Underpinning theory of the intervention	Not clearly articulated. The Maikuru Program was an adult-teen mother mentoring program developed by the University of Pittsburgh, Department of Family Medicine beginning in 2009. "Maikuru," derived from the Shona language of southern Africa meaning "wise woman of the village," is the concept that helped shape the vision of the program. The Maikuru mentoring program was designed to 1) improve adolescent mothers' life skills; 2) increase their social support networks; 3) delay a second pregnancy prior to completing their teen years; and 4) achieve their educational goals – all of these by the components mentioned in the intervention.
Challenges & strategies	Low response rate for follow-up and its attendant potential for bias.<br>Lessons learnt: This program was based on a thorough search of teen mentoring programs and was adapted to meet the needs of the teen mothers. The focus lay firmly on clinical observations of teen mothers and consisted of screening for and education about depression, birth control, completing educational and professional goals, and relationships with the child's father as well as new boyfriends. The program was intended to help them better care for their current child and delay conceiving again until they had completed high school and had developed better skills for meeting their own financial needs. Practitioners who care for adolescent mothers should be aware that they may not have sup- portive adult relationships and only their pregnant friends to offer advice and support. Future research programs should focus on intensive training of the adult mentors, supporting the adult-teen mentoring relationship, enrolling teens before they have given birth or while they are still in high school, using as many strategies as possible to maintain contact, such as collecting contact information from family and friends, and build upon the strength of racial congruence between the research team and the participants. To address the lack of a control group, a future study might consist of a randomized controlled trial that offers an educational program compared with a mentoring/educational program.
What is new?	Educational achievement and employment were high among a modest proportion of adolescent mothers who had participated in a culturally tailored, teen mother-adult mentoring program. Repeat teen pregnancy was infrequent and the mentoring program was perceived as contributing to the success of those who responded to the follow-up. A future randomized trial based on this model may confirm these findings.
Additional references / Other comments	Bassline: there were no significant differences between dropouts and completers in race (P 1⁄4 0.117) or age (P 1⁄4 0.464). Completers were more likely to still be in high school and less likely to have dropped out of high school than program dropouts (P 1⁄4 0.016).<br><br>Efficacy of a randomized cell phone-based counseling intervention in postponing subsequent pregnancy among teen mothers. Matern Child Health J, 15(1), 42e53.<br><br>abeea'h, W. A., Hendry, M., Booth, A., et al. (2017). Inter- vention Now to Eliminate Repeat Unintended Pregnancy in Teenagers (INTERUPT): a systematic review of intervention effectiveness and cost-effectiveness, and qualitative and realist synthesis of implementation factors and user engage- ment. BMC Med, 15(1), 155.

**Quality Assessment – Lin 2019**	*Reviewer initials: CFT/AMK*
Critical Appraisal	*The Mixed Methods Appraisal Tool (MMAT) 2018 will be used for quantitative, qualitative and mixed-methods studies.*<br><br>**Screening questions**<br>1. <u>Are there clear research questions?</u> — Yes<br>Yes, to examine educational attainment, employment, and pregnancies of these adolescent mothers 1-5 years post program.<br><br>2. <u>Do the collected data allow to address the research questions?</u> — Yes<br>Yes, but low response rate<br><br>**MMAT for quantitative descriptive**<br>1. <u>Is the sampling strategy relevant to address the research question?</u> — Yes<br>Yes, multi-approach recruitment strategy well described for both girls and mentors<br><br>2. <u>Is the sample representative of the target population?</u> — CT<br>It looks not very representative from the programme or US population, low response rate<br><br>3. <u>Are the measurements appropriate?</u> — Yes<br>Yes, descriptive survey to measure long term outcomes of interest.<br><br>4. <u>Is the risk of nonresponse bias low?</u> — No<br>Yes, very low response rate and from teens with higher educational achievement and employment rates.<br><br>5. <u>Is the statistical analysis appropriate to answer the research question?</u> — Yes<br>Yes, categorical data were summarized by frequency and percentage; continuous data by means and ranges. Kruskal-Wallis tests used to compare the differences in age and educational level between survey responders and non-responders.

**Study ID: Fernandez Turienzo 2025**	*Reviewers initials: HRJ/KDB*

Reference:

Fernandez Turienzo C, November L, Kamara M, Conteh O, Kingsford AM, Koroma PJ, et al. Community-based mentoring to reduce maternal and perinatal mortality in adolescent pregnancies in Sierra Leone (2YoungLives): a pilot cluster-randomised controlled trial. The Lancet. 2025 Jun 18.

Other publications from same study:

Trial registration: ISRCTN32414369 2 Young Lives: mentoring teenagers for safer pregnancy and birth in Sierra Leone. https://doi.org/10.1186/ISRCTN32414369

Fernandez Turienzo C, Kamara M, November L, Kamara P, Kingsford AM, Ridout A, Thomas S, Seed PT, Shennan AH, Sandall J, Williams PT. A community-based mentoring scheme for pregnant and parenting adolescents in Sierra Leone: Protocol for a hybrid pilot cluster randomised controlled trial. Plos one. 2024 Mar 25;19(3):e0294538.

M Kamara, L November, CF Turienzo, J Sandall, A Shennan. 2YoungLives: saving lives of adolescent girls and their babies in Sierra Leone. BJOG, 2022, 129, 95 | added to CENTRAL: 31 October 2022 | 2022 Issue 10 https://www.cochranelibrary.com/central/doi/10.1002/central/CN-02465151/full (Conference proceeding)
November L and Kamara M. 2 Young Lives: mentoring teenagers for safer pregnancy and birth. Project report 2020. 2YoungLives. Available from: https://2younglives.org/wp-content/uploads/2020/07/2YL-2020-report-final.pdf (Accessed 1 August 2023)

Methods	**Study design**: cluster hybrid randomised controlled pilot trial<br>**Primary aim**: to evaluate the feasibility and potential effects of 2YoungLives<br>**Intervention**: 2YoungLives: a community-based mentoring program (matched) from pregnancy up to 1 year after birth.<br>**Baseline measurements**: maternal age, sickle cell disease, Ebola survivor status, disability, parity, multiple pregnancy, and blood pressure measurements.<br>**Duration of study**: 2022-2024
Participants	**Setting**: Five communities (rural and urban) in five districts of Sierra Leone<br>**Inclusion criteria**: All pregnant adolescent girls younger than 18 years living in those cluster communities and presenting for maternity care were eligible.<br>**Total number of participants**: Six clusters (372 girls) were randomised to the intervention group and six clusters (301 girls) to the control group.<br>**Mean age of participants (years) (SD):** 2Younglives: 16·26 (0·84); control: 16·52 (0·76)<br>**Mean gestational age at first visit (weeks)**: Not reported.<br>**Mean gestation age at time of intervention (weeks)**: Not reported.<br>**Ethnicity**: African<br>**Marital status**: Not reported<br>**Socio-economic status**: Not reported but socio-economically vulnerable girls.<br>**Education**: Not reported<br>**Other lifestyle/health behaviors**:<br>Sickle cell disease: 2Younglives: 3/360 (1%); control: 0/275<br>Ebola survivor status: 2Younglives: 1/359 (<1%); control: 0/275<br>Disability: 2Younglives: 8/360 (2%); control: 1/275 (<1%)<br>Parity (0): 2Younglives: 328/360 (91%); control: 264/276 (96%)<br>Multiple pregnancy: 2Younglives: 7/357 (2%); control: 2/274 (1%)<br>Blood pressure BP (abnormal, when available): 2Younglives: 6/345 (2%); control: 2/266 (1%)
	<u>From the pre-trial (informal report November 2020):</u> 80% girls from Kuntorloh, 20% from Port Loko, Sierra Leone. All girl is under 18 years of age or under 20 if disabled. training was added to the training package for mentors, as deafness appears to be a common disability. Total number of participants: 73 (55 Freetown, 18 Port Loko). Married (living with husband): Freetown 0 (%); Port

	Loko 2 (11%). Both parents alive: Freetown 38 (67%); Port Loko 15 (83%); mother alive: Freetown 42 (76%); Port Loko 17(94%); living with mother: Freetown 14 (24%); Port Loko 9(50%; living at boyfriend's family home: Freetown 9 (16%); Port Loko 2 (11%). Percentage whose mother is alive who are living with their mother: Freetown 11 (26%); Port Loko 10 (59%)
Intervention	**Type and content of intervention**: 2YL mentoring scheme + standard maternity care<br><br>Components (activities, sessions, characteristics, or behaviours):<br>The intervention comprised four core components: community engagement and involvement (CEI); recruitment, training, and supervision of mentors; mentor–mentee matching; and mentoring and additional activities.<br><br>CEI: LNP devised a three-visit community engagement and involvement strategy to ensure the intervention's acceptance and tailoring to local contexts, engaging those who could provide diverse perspectives. Building trusting relationships through listening, discussing, and connecting with communities was crucial, with additional community engagement and involvement visits made as needed.<br><br>Recruitment of mentors: Women passionate about supporting vulnerable girls were recruited as mentors based on their experience, community knowledge, commitment, kindness, and trustworthiness, in collaboration with community stakeholders. They are taken on as volunteers and given a monthly stipend. Each cluster had a team of four mentors and one coordinator who was recruited to support mentors and communicate with the central LNP team<br><br>Training and supervision of mentors: All mentors and coordinators received a 4-day manualised training programme covering basic maternal, newborn, and infant health, and topics such as confidentiality, safeguarding, and good communication (also use of scenarios and role play, and the use of the pictorial maternal and infant health resources). Ongoing support and supervision were provided by local coordinators and the central LNP management team.<br><br>Recruitment of girls: Eligible adolescent girls were enrolled as they came forward to a maximum of 36 (three to four girls per mentor) girls per cluster. This process helped us to assess the feasibility of the intervention in a pragmatic way while avoiding selection bias. Girls could join at any stage of pregnancy and received mentoring for 1 year after birth regardless of their pregnancy outcome.<br><br>Matching: The coordinator matched mentors and girls based on geographical proximity and shared language.<br><br>Additional activities: least weekly face-to- face meetings between mentors and mentees for comfortable and confidential conversations; promoting health services uptake (eg, supporting mentees to register at a government health facility, and inviting midwives to monthly meetings to break down barriers to attendance); reminding mentees to attend or attending with them for antenatal care; accompanying mentees to health facilities during labour or for emergency care (or ensuring another birth partner was available) and advocating for skilled birth attendants; visits from mentors to mentees' families to advocate for family support if appropriate; flexible support for pregnancy and parenthood depending on the mentee's network; discussing small business options with mentees and accompanying them to purchase initial supplies; encouraging and subsidising return to school or vocational training; practical sessions on making healthy baby food; promoting postpartum contraception; and emphasising the importance of early health-seeking behaviour for their babies. Monthly site meetings with all mentors and mentees provided peer support, cooking, eating, and group discussions. Visitors such as health-care providers, teachers, and community members attended to discuss health topics and educational opportunities.<br><br>Duration of mentoring program: 12 months after birth. Follow ups: 1 year.<br><br>Safeguarding, support issues (e.g. clean criminal records): Training provided.

	**Type and content of control (if applicable): T**he intervention and control groups received usual maternity care, following local and national guidelines for Sierra Leone,20,21 based on the 2016 WHO antenatal model. This model recommends a minimum of eight antenatal contacts to provide quality, respectful, person-centred care. The National Reproductive, Maternal, Newborn, Child, and Adolescent Health Strategy outlines interventions for positive pregnancy and prioritises skilled birth attendance and essential newborn care within emergency obstetric and newborn care.
Outcomes	**Outcome considered in the review and reported in the study:**<br>**From cluster trial:**<br>Composite of maternal and perinatal mortality: 2YoungLives: 23/361 (6%); control: 35/279 (13%)<br>Maternal death: 0/361); control 1/279 (<1%)<br>Stillbirth: 12/361 (3%); control: 17/279 (6%)<br>Neonatal death: 11/361 (3%); control: 18/279 (6%)<br><br>Post-abortion care: 7/358 (2%); control: 4/277 (1%)<br>Vaginal births: 316/345 (92%); control: 248/266 (93%)<br>Assisted vaginal births: 1/345 (<1%); control: 3/266 (1%)<br>Caesarean sections: 20/345 (6%); control: 9/266 (3%)<br>Vaginal breech: 8/345 (2%); control: 6/266 (2%)<br>Preterm birth: 97/332 (29%); control: 83/261 (32%)<br>Births attended by skilled health professionals: 2YL: 254/346 (73%); control: 109/266 (41%)<br>Place of birth: community: 2YL 21/346 (6%) ; control: 26/267 (10%); peripheral health unit: 2YL: 239/346 (69%); control: 192/267 (72%); or hospital): 2YL: 86/346 (25%); control: 49/267 (18%).<br>Complications (malaria, obstetric fistula, hypertension, pre-eclampsia or eclampsia, haemorrhage, fever, anaemia, or hysterectomy):2YoungLives >50%; <50% control.<br><br>Apgar score greater than 7 at 5 min: 11/295 (4%); control: 8/224 (4%)<br>Mean birthweight: 2895·90 (468·37)); control: 3048·34 (386·23)<br>Resuscitation: 119/317 (37%); control: 71/242 (29%)<br>Immediate breastfeeding: 307/316 (97%) control: 236/243 (97%)<br>Kangaroo mother care: 248/318 (78%)); control: 203/247 (83%)<br>Admission to neonatal intensive care unit: 6/320 (2%); control: 4/243 (2%)<br><br>Additional process outcomes in appendices (no diff between groups): timing and quality of first antenatal visit (under 12 weeks' gestation, provision of long-lasting insecticidal net, HIV and syphilis testing, total number of antenatal checks (including those with blood pressure), total number of antenatal visits and number of visits with BPs, Iron and folic acid supplementation , Intermittent preventive treatment of malaria in pregnancy (IPTp), albendazole - deworming medication, tetanus-diphtheria vaccinations, Delivery monitored with partograph, Uterotonic prophylactic given immediately after delivery, referral (e.g. obstetric fistula, hypertensive disorders, haemorrhage, fever, anaemia, hysterectomy), postnatal contraception, infant immunisations.<br>Process and economic evaluations are ongoing.<br><br>**From informal report (pre-trial):**<br>Maternal mortality: data from 2015 survey: 13%; 2YL: 0%<br>Neonatal mortality: data from 2015 survey: 16%; 2YL: 6%<br>Infant mortality: data from 2015 survey: 26%; 2YL: 11%<br>Place or birth and skilled birth attendance: 61/73 with data: 3/61: home (but with a local nurse) and 1 (2%) happened in the absence of a skilled birth attendant.<br>Did she use a bed-net: Yes: 32 (65%); No 17(35%)<br>Did she have malaria in pregnancy: Yes 4 (12.5%); No: 12 (70%)<br>Family planning: 70% of mothers whose babies were over 1 year were using contraception<br>Second pregnancies: 0.

Small business, training, and education:

- 100% girls ran a small business throughout pregnancy and in the first postnatal year (though they varied in their success, at a minimum this allowed all the girls to eat well every day)
- Most girls had been at school when they became pregnant, and due to the previous ban on visibly pregnant girls attending school/sitting exams (until 2020), had to leave. A change of government policy in 2019 means that secondary schooling is now officially free, although expenses such as uniform and equipment still present barriers to some.
- Of the mothers for whom this data is known (n=25), in the second postnatal year:
  9 were back at school
  2 were on a vocational training course subsidised by 2YL
  2 were continuing to expand their business,
  12 were doing 'nothing'.

High levels of poverty and vulnerability
The majority of girls were told to leave the home where they were staying when they revealed their pregnancy, and mentors felt that even when girls initially came from a home with some financial security, they were still very vulnerable due to this situation.
'Being driven' and reconciliation
Although having a mentor was not a magic bullet for family reconciliation, mentors try to meet all the girls' families as a gesture of respect and to try to build a relationship which might then allow the girl to re- enter the family home after the birth.
Monthly gatherings and peer support
Once a month, all the mentors, the co-ordinator and all the mentees gather to cook and eat together, and to discuss health topics. This has proved to be an extremely popular aspect of the programme. When asked what they liked most about being mentored, most girls identified this monthly gathering, with eating and having fun together and playing with each other's babies as the main factors.
Mentors/mentee contacts
Mentors are required to provide at least one opportunity per week for a 1:1 meeting. However, co-ordinators report almost daily contact. Even at one contact per week antenatally and one per fortnight postnatally, this equates to an average of 22 antenatal contacts and 26 postnatal contacts per mentee, or over 4000 mentor/mentee contacts in total since the project commenced
Accessible health education
Prior to starting the pilot, the authors came across pictorial health education resources which had been developed and tested locally. These laminated picture cards portray common health issues and are designed to be used in a facilitated discussion with community members. They are used in this way as part of the monthly gatherings and then again 1:1 between mentors and mentees to reinforce the message.
Improving confidence and self-efficacy
Despite some of the challenges already discussed, most girls manage to run a successful business for the first time, which allows them to eat well every day and save for their babies' needs. Having been part of the health discussions throughout their pregnancy, girls become confident in their understanding of basic health advice, such as the need to breastfeed exclusively for 6 months, and what to do if their baby had a fever or diarrhoea. Several girls go on to do the health talks at the monthly gatherings as they grow in confidence
Wider community impact
The health messages are effective not just for the young women being mentored, but for the community more generally as mentors share them more widely. For example, one mentor reported that she now tells all pregnant women that carrying very headwater containers could lead to bleeding and miscarriage, and another that she now understands that the common advice for pregnant women to restrict their food intake to ensure their baby is small and easy to deliver is wrong, and she is encouraging all pregnant women to eat well. This is an encouraging finding as it indicates a wider impact for the community than just the mentees of the scheme.

Supporting small businesses
It was recognised that an important factor in determining birth outcomes in this group is their lack of economic capital. This resulted in most girls not eating adequate quantity or quality of food and

	having no means to purchase medicines prescribed in pregnancy, or items for their births or babies. Having a small start-up fund to enable them to engage in petty trading was identified as a key element of the scheme.<br>*The role of the mentor as business advisor*<br>The role of mentors in relation to these small businesses is to help the girls identify a suitable business that will be sustainable and profitable, and help with issues such changing what the girl sells depending on seasonal supply and demand (for example if a girl sells lunch to school children, the mentor helps her to flex during school holidays).
Underpinning theory of the intervention	For the trial: a theory of change was co-developed and relationship building, engagement and advocacy, educational, social and economic empowerment, and respectful community engagement and involvement were important mechanisms of action to consider. It was hypothesised that 2YoungLives could save lives and improve the health and wellbeing of pregnant adolescents and their babies while simultaneously enhancing livelihoods through relationship building, access and engagement with maternity services, advocacy with families and health workers, empowerment (health, social, and economic) and respectful community Engagement.<br>A review of the determinants of delivery service use identified low maternal age as a determinant for not accessing skilled care for delivery, and evidence from other community projects in Freetown indicate that having an advocate when accessing health care helps to reduce some of the barriers such as disrespectful care and informal charges.
Challenges & strategies, lessons learnt	The trial paper cites some challenges and how they were addressed and embedded as opportunities. For example. in a very remote cluster, parents hesitated to support the intervention due to previous unmet promises from non-governmental organisations. The LNP implementing team addressed this by bringing key stakeholders from this cluster to Freetown to witness former mentored girls working as plumbers or electricians and supporting their families, which led to the stakeholders subsequently encouraging participation.<br><br>Three clusters had initially low recruitment, prompting the local research teams and investigators to further investigate. Discussions were held with local community members and stakeholders, health-care providers, and other non-governmental organisations to understand why fewer adolescent girls were attending antenatal care compared with previous years and similar sites. Proposed explanations included successful contraception programmes (not indicated by facility-level data), a preference for a new clinic in the same community that provided more respectful care, over-reporting of age due to stigma and fear of punitive policies for underage pregnancies, or avoiding care at facilities for similar reasons. In the intervention group, the LNP implementing team engaged with community leaders and health-care providers to understand and alleviate these concerns, rebuilding trust and ensuring access to maternity care for the girls
What is new? / Conclusions	2YoungLives was feasible to implement in urban and rural communities and significantly reduced the combined rate of maternal and perinatal deaths by 48%, primarily driven by a significant reduction in perinatal mortality.<br>There are so many stories of impact at many different levels, girls returning to education, not having second pregnancies, becoming plumbers, empowered mentors who support each other and become agents of change in their communities leasing with healthcare providers, chiefs, teachers; and also clear changes in community mindsets about gender norms, blood donation etc. Process and economic evaluations ongoing.
Additional references / Other comments	The reduction in mortality likely stems from several mechanisms. Mentors' health knowledge and their advocacy for early access to care, escalation, and referral during complications were important factors. This support, combined with girls' empowerment and the mentors' companionship during visits, may have positively influenced provider behaviour, thereby improving the quality of antenatal and intrapartum care (such as ensuring a skilled birth

	attendant). The intervention also improved girls' confidence in seeking care, while the economic empowerment from setting up small businesses allowed them to make independent decisions and pay for their own transport to the hospital. Additionally, in some clusters, community members assisted with hospital transfers and arranging blood donations.

**Quality Assessment – Fernandez Turienzo 2025**	*Reviewer initials: HRJ/KDB*
Critical Appraisal	*The Mixed Methods Appraisal Tool (MMAT) 2018 will be used for quantitative, qualitative and mixed-methods studies.*<br><br>**Screening questions**<br>1. <u>Are there clear research questions?</u> Y<br>The paper clearly states the aim was to evaluate the feasibility and potential effects of the 2YoungLives community-based mentoring intervention and to assess implementation feasibility to inform a subsequent larger trial<br><br>2. <u>Do the collected data allow to address the research questions?</u> Y<br>The study collected data on implementation feasibility (e.g., recruitment, implementation processes) and the primary outcome, which directly addresses the stated research aims.<br><br>**MMAT for quantitative RCT**<br>1. <u>Is randomization appropriately performed?</u> Y<br>The paper specifies that allocation of the 12 clusters was performed using a computer-generated central randomisation system (Medscinet). A minimisation algorithm was also used to balance the groups based on cluster size and distance to the referral hospital<br><br>2. <u>Are the groups comparable at baseline?</u> Y<br>The authors state that the characteristics of the girls in the intervention and control groups were similar at baseline (Table 1)<br><br>3. <u>Are there complete outcome data?</u> Y<br>Largely complete. The study reports a low rate of loss to follow-up, accounting for less than 10% in both groups. The authors also note the minimal missing data as a strength.<br><br>4. <u>Are outcome assessors blinded to the intervention provided?</u> N<br>Masking of participants, health-care providers, and local investigators was not possible once the intervention commenced. However, the study assignment was masked for the statistician and the researchers who analysed the data.<br><br>5. <u>Did the participants adhere to the assigned intervention?</u> Yes<br>Not all eligible participants in the intervention clusters received the intervention as this was an intentional feature of the pilot's pragmatic design, which capped enrolment at a maximum of 36 girls per cluster. However most girls in the intervention sire who enrolled in 2YL adhered to the intervention (see per protocol analysis).

**Study ID: Quint 1991**	*Reviewers initials: CFT/AHK*

Reference:

Quint J. Project Redirection: Making and measuring a difference. Evaluation and Program Planning. 1991 Jan 1;14(1-2):75-86.

Other publications from same study:

Polit DF and Kahn JR. Needs and Characteristics of Pregnant and Parenting Teens. The Baseline Report for Project Redirection. New York: Manpower Demonstration Research Corporation; 1982

Levy SB and Grinker WJ. Choices and life circumstances: an ethnographic study of Project Redirection teens. 1983; New York: Manpower Demonstration Research Corporation.

Polit DF, Kahn JR. Project redirection: Evaluation of a comprehensive program for disadvantaged teenage mothers. Family Planning Perspectives. 1985 Jul 1:150-5.

Branch A, Riccio J and Quint J. Building self-sufficiency in pregnant and parenting teens: final implementation report of Project Redirection. 1985; New York: Manpower Demonstration Research Corporation.

Polit D, Khan J and Stevens D. Final Impacts from Project Redirection. A Program for Pregnant and Parenting Teens. New York: Manpower Demonstration Research Corporation; 1985.

Tannen MB. Evaluating the impacts of Project Redirection: A program for disadvantaged pregnant and parenting teens. 1986. *Papers in the Social Sciences, 5,* 81–93.

Polit DF, Kahn JR. Early subsequent pregnancy among economically disadvantaged teenage mothers. American Journal of Public Health. 1986 Feb;76(2):167-71.

Polit DF, Quint JC and Riccio. The Challenge of Serving Teenage Mothers. Lessons from Project Redirection. 1988. New York: Manpower Demonstration Research Corporation.

Polit DF. Effects of a comprehensive program for teenage parents: Five years after Project Redirection. Family Planning Perspectives. 1989 Jul 1:164-87

Methods	**Study design**: mixed methods (quasi experimental study and linked ethnography, implementation, impact analysis, interview questionnaire)<br>**Primary aim**: to improving employment, welfare, and child-related outcomes among disadvantaged school-age mothers.<br>**Intervention**: compressive program that linked enrollees with existing educational and health services and supplemented these with workshops on parenting, employability, and life management, and with group and individual counselling. It also paired teens with adult “community women,” who were mentors, role models, and friends.<br>**Baseline measurements**: age at enrolment, ethnicity, household status, AFCD receipt, education level, pregnancy/parenting status, receipt of services at enrolment.<br>**Duration of study**: 1980-1986
Participants	**Setting**: US: first phase four locations (El Centro de Cardinal in Boston, the Harlem (New York) YMCA, Chicanos por la Causa in Phoenix, and the Children’s Home Society in Riverside, CA)<br>**Inclusion criteria**: ethnically mixed group of pregnant teenagers and mothers who were I7 or younger, lacked a high school diploma, and were receiving or eligible for AFDC. Sites initially drew almost all of their participants from hospitals, health clinics, welfare offices, schools, and graphic and socioeconomic characteristics of the 805 other agencies serving young mothers.<br>**Total number of participants**: (enrolled): 805<br>**Mean age of participants (years) (SD) (enrolment):** 16.4<br>**Mean gestational age at first visit (weeks)**: Not reported.<br>**Mean gestation age at time of intervention (weeks)**: Not reported (some pregnant, some postnatal)<br>**Ethnicity**:<br>Black: 43.5 ; Hispanic: 44.7; White; Other: 1.7<br>**Marital status**: Never married: 93.9

	**Socio-economic status**: <u>Household status</u>:<br>Head of household: 7.9<br>Living in two-parent household: 14.5<br><u>Receiving AFDC (%):</u> 71.6<br>**Education**:<br>Out of school at enrolment (%): 59.1<br>Percent out of school who left school before pregnancy (%): 49.9<br>Mean number of months out of school: 13.4<br>Mean highest grade completed: 8.9<br>**Other lifestyle/health behaviors**:<br><u>Pregnancy status (%):</u><br>Pregnant with first child: 56.3<br>Pregnant with subsequent child: 4.4<br>Parent, Not pregnant: 39.3<br><u>Receipt of services before entering Redirection (%)</u><br>Prenatal care (if pregnant): 95.9<br>Pediatric care (if parents): 87.1<br>Child care (if parents): 82.6<br>Family planning: 21.9<br>Employment: 2.9<br><br>The ethnography study sheds further light on the severity of the problems some young women faced. Of the 18 teens studied in depth, two had been raised by abusive parents (and one was herself suspected of abuse). Two teens were frequently mistreated by their boyfriends, and alcoholism and drug use marked the home environments of a significant minority, The lives of most participants were less troubled, and the majority of young women could and did turn to their mothers for psychological support. But there were often disagreements between mother and daughter about who would take care of the baby and when, or about whether the mother could impose restrictions on her daughter's activities. Moreover, although very few of the teens in the program had intended to become pregnant, many hoped that having a child would bring about a positive change -a solidified relationship with a boyfriend, for example, or greater autonomy. Often, these changes were not forthcoming. In view of the educational gaps, economic handicaps, and psychosocial stresses they confronted, the challenge of "redirecting" program enrollees was a sizeable one.
Intervention	**Type and content of intervention**: Project redirection.<br><u>Components (activities, sessions, characteristics, or behaviours)</u>:<br>- The Project Redirection treatment involved a mixture of messages, relationships, and services. Many of the messages were straight forward and pragmatic, emphasizing the importance of good perinatal and paediatric care, high school completion and preparation for work, knowledge about child development and childcare, and regular use of birth control. Underneath these explicit messages, staff were convinced that what the teens needed most was increased self-esteem, and they saw this as a precondition both to immediate service utilization and to ultimate self-sufficiency. They were concerned, therefore, about creating a warm, supportive, and non-judgmental environment in which teens would feel free to share their problems with and learn from the experiences of others.<br>- Several afternoons a week, the programs offered informational workshops, and group as well as individual counselling were regularly scheduled for all participants.<br>- Project staff typically included a program director, several counsellors, and a person who coordinated the activities of the community women ('mentors'). Staff led the group counselling and often conducted workshops, in addition to meeting with individual participants. Most were social workers or others with experience in the human services.<br>- <u>**The community woman component/mentors**</u>: most distinctive element. Community women, each of whom was matched to between one and five teens (usually from the same ethnic background), performed many different functions: they served as the teens' confidantes, escorted them to appointments, took them shopping or to recreational events, made

reminder or even wake-up phone calls, and acted as paraprofessional case managers, keeping track of participants' attendance at scheduled activities and relaying information back to staff. In so doing, they reinforced the program's messages and offered role models of effective parenting and of coping with the problems of everyday life. Time commitment: They were required to spend a minimum of 5 hours per week with each teen, and for this, received a $15 weekly stipend intended to defray expenses.

- **Service delivery component**: individualization and brokerage. Unusually comprehensive mix of services with considerable emphasis on employability development, with workshops covering such topics as how to complete a job application and proper on-the-job behaviour. (The program did not include actual vocational skills training.) Three other service areas were designated as of major importance: health, education, and "life management" (a term covering topics as diverse as nutrition, parenting education, family planning, budgeting, and assertiveness training). Recreational activities, transportation assistance, and child care were also available. Program guidelines specified that all teens were to receive services in all of these areas, but also recognized that each teen had different strengths and needs, thus an individual Participant Plan (IPP) was drawn up jointly by the enrollee, her community woman, and program staff. The IPP specified short- and long-term objectives for the participant, as well as the services and activities that would help her move toward these goals. Her progress was monitored monthly, and the IPP could be revised to respond to new circumstances.
- From the beginning, it was understood that the sponsor agencies should avoid duplication of services through "brokerage"- that is, bringing together and coordinating on behalf of program participants services already available in the community, rather than providing such services on their own. Brokerage could entail either referring teens to other agencies or inviting representatives of these agencies to deliver workshops at the Redirection sites.

Recruitment of mentors: A major open question at the outset of the demonstration was whether women from low-income communities would come forward in sufficient numbers. They did so, from many sources (including churches and other local organizations, and often on the referral of a friend) and for many reasons. A number had themselves been teenage mothers and wanted to help others realize opportunities they had missed. Others wanted to learn more about their own children, and still others simply wanted to volunteer. They should be able to devote the requisite time to the program, foster its goals, demonstrate community involvement, and be capable of producing written reports and filling out forms. Otherwise, it set no criteria for local sites to follow in selecting the community women, reasoning that each sponsor agency would best known both the sources for recruiting the women and the needs of the teens they served. As a result, the community women were a diverse group, both within and across the sites. Ranging an opportunity and a place to socialize-all of these in age from the early 20s to the late 7Os, half were married, about one in seven had never married, and the rest were divorced, separated, or widowed. Five out of six had at least a high school diploma or its equivalent. The majority were not working when they joined the program, and just over a quarter were receiving welfare.

Training and supervision of mentors: critical to the success of the component. They received several days' training aimed both at helping the women clarify their own values and attitudes with regard to teen pregnancy and parenthood and at imparting concrete information on available social services, communication skills, and documentation procedures. These sessions also gave staff the opportunity to observe and dismiss women who appeared inappropriate and permitted women who decided that the position was not right for them to exit gracefully. Over the course of the demonstration, periodic in- service training sessions were held to sharpen skills and allow the community women to share problems and solutions.

Recruitment of girls: Ethnically mixed pregnant teenagers and mothers who were I7 or younger, lacked a high school diploma, and were receiving or eligible for AFDC. Sites initially drew almost all of their participants from hospitals, health clinics, welfare offices, schools, and graphic and socioeconomic characteristics of the 805 other agencies serving young mothers.

Matching: Each community woman was matched to between one and five teens (usually from the same ethnic background).

	<u>Planned duration of the programme</u>: 18 months.<br><br><u>Follow ups</u>: 1, 2 and 5-year follow-up.<br><br><u>Safeguarding, support issues</u> (e.g. clean criminal records): Not reported.<br><br>**Type and content of control (if applicable):** Comparison sample: comparison group was recruited from among teens who met the Redirection eligibility criteria and who were receiving services from community agencies in cities that did not offer the Redirection program. These cities were selected to be similar to those in which Redirection operated along a variety of socioeconomic and geographic indicators and in terms of the availability of services for adolescent mothers at the inception of the demonstration.
Outcomes	**Outcome considered in the review and reported in the study:**<br><u>Implementation: Patterns of Program Services Receipt and Attendance</u>:<br>Clinic visits:<br>Maternal health: ever scheduled 95.1; ever participated 90.4; attendance rate 96.8<br>Infant health: ever scheduled 83.9; ever participated 83.2; attendance rate 98.1<br><br>Life management<br>Family planning: ever scheduled 71.3; ever participated 63.7; attendance rate 77.3<br>Parenting education: ever scheduled 76.8; ever participated 68.4; attendance rate 64.5<br>Any life management activity: ever scheduled 96.6; ever participated 88.5; attendance rate 69.1<br><br>Education<br>Public school: ever scheduled 20.9; ever participated 19.8; attendance rate 78.6<br>GED program: ever scheduled 9.2; ever participated 49.2; attendance rate 48.1<br>Alternative school: ever scheduled 26.6; ever participated 24.3; attendance rate 75<br>Any educational activity: ever scheduled 79.7; ever participated 73.5; attendance rate 62.<br><br>Employability activities<br>World of work seminars: ever scheduled 75.1; ever participated 63.8; attendance rate 61.9.<br>Individual vocational counselling: ever scheduled 59.9; ever participated 55.9; attendance rate 85.1.<br>Job training: ever scheduled 23.2; ever participated 22.2; attendance rate 83.4.<br>Any employability related activity: ever scheduled 81.9; ever participated 70.6; attendance rate 67.3.<br><br><u>Measures of fertility and contraceptive use at year 5</u><br>Mean no. of pregnancies: project redirection 3.1; comparison 2.9<br>Mean no. of abortions: project redirection 0.3; comparison 0.5<br>Mean no. of live births: project redirection 2.4; comparison 2.0<br><br>% who used a method at last intercourse: project redirection 70; comparison 69<br>% who used the pill at last intercourse: project redirection 43; comparison 41<br>% sterilized: project redirection 12; comparison 14<br><br><u>Main education, employment and welfare dependency impacts at 1 year, 2 years and 5 years</u><br>% ever enrolled in school between baseline and 1 year after baseline: project redirection 75; comparison 51<br>% ever enrolled in school between baseline and 2 years after baseline: project redirection 87; comparison 71<br>% with diploma/GED certificate at 1 year 9: project redirection 9; comparison 7<br>% with diploma/GED certificate at 2 years 20: project redirection 20; comparison 20<br>% with diploma/GED certificate at 5 years: project redirection 48; comparison 48

	% employed at 1 year: project redirection 14; comparison 12<br>% employed at 2 years: project redirection 15; comparison 15<br>% employed at 5 years: project redirection 34; comparison 28<br>% ever employed between baseline & 1 year after baseline: project redirection 49; comparison 38<br>% ever employed between baseline & 2 years after baseline: project redirection 61; comparison 54<br>Mean weekly hours worked at 5 years: project redirection 13; comparison 9<br>Mean weekly earnings at 5 years S: project redirection 68$; comparison $45<br>Mean household income at 5 years: project redirection 737$; comparison 756$<br><br>% in an AFDC household at 1 year: project redirection 70; comparison 70<br>% in an AFDC household at 2 years: project redirection 75; comparison 68<br>% in an AFDC household at 5 years: project redirection 49; comparison 59<br><br>* The impacts at 1 and 2 years after baseline are based on analyses with the full research sample (675 young women); 5-year impact on the 5 year subsample (277).<br><br><u>Main childrearing, Parenting, and Child Development Outcomes at 1 year, 2 years and 5 years</u><br>% with a subsequent pregnancy by 1 year after baseline: project redirection 14; comparison 22<br>% with a subsequent pregnancy by 2 years after baseline: project redirection 45; comparison 49<br>Mean number of pregnancies at 5 years: project redirection 3.1; comparison 2.9<br>Mean number of abortions at 5 years: project redirection 0.3; comparison 0.5<br>Mean number of live births at 5 years: project redirection 2.4; comparison 2.0<br><br>Mean home environment score at 5 years*: project redirection 44; comparison 40<br>% who enrolled children in Head Start by 5 years after baseline: project redirection 47; comparison 34.<br>% who breastfed child (pregnant at baseline group): project redirection 50; comparison 20<br>Child's mean problem behaviour score at 5 years: project redirection 92; comparison 105<br>Child's mean vocabulary score at 5 years: project redirection 86; comparison 80<br><br>* Home Observation for the Measurement of the Environment (HOME) scale used to measure parenting skills too. It consisted of 55 self-reported and observational items that measured the mother's degree of warmth and acceptance of her child and the extent to which the home environment was stimulating and enriching for the child.
Underpinning theory of the intervention	Programme theory and programme mechanisms (i.e. the community women, the individual participant plan and the peer group sessions) are described in the implementation analysis paper 2. (See more details there). Redirection treatment includes both the receipt of services and interaction with caring adults, in the process of which number of close personal relationships are formed. These interventions services and relationships are made available to Project Redirection participants in support of the program objectives of continued education; acquisition of knowledge and skills leading to employment; delay of subsequent pregnancies; and increased personal and economic self-sufficiency. More explicitly, the provision of these services and support primary goals: to bring immediate benefits to the participants and their children, and to influence participants to adopt the attitudes and behaviours essential to meeting the program objectives.
Challenges & strategies, lessons learnt	**Mentors**: Important lessons emerging is that, within certain constraints, very different women could fulfil this role successfully. On one hand, similarity of ethnic or socioeconomic background helped ensure at least some shared body of experience and facilitated comprehension of the teens' life circumstances. But beyond this, it appears that a non-judgmental approach and an ability to communicate with the teens and understand their concerns were the most important determinants of success in the community woman role.

The research does not answer the question whether the community woman component made a difference from the standpoint of program impacts. But interviews with teens held after they left the program indicated that the majority felt their community woman was “important” or ‘very important” The relationship was often especially close when teens became estranged from their families. At the other extreme, some felt their community woman was either distant or nosy, and alienation from the community woman was a major source of dissatisfaction with the program as a whole.
Turnover in the community woman component was especially jarring to teens, who often found it hard to transfer their confidence and affection from one community woman to another. And turnover, which is common in programs involving volunteers, was high in Project Redirection: Only 229 of the women ever enrolled were still active at the end of 1982. The problem was eased as staff came to understand that community women joined the program to meet some of their own needs as well as those of the teens. Providing community women with rewards and recognition, giving them an opportunity and a place to socialize-all of these helped build commitment to the program.

Brokerage model: Perhaps the chief advantage of the brokerage model was economy as it enabled the sites to contain the direct cost of operating the program. Across the sites, this averaged $3,540 per participant, or $3,890 per service year (Le., the cost of maintaining a participant in the pro- gram for a full year). Three-fourths of this amount was used for program management (including planning, ad- ministration, and reporting), and for services offered directly by the sites. The figure does not include costs borne by outside agencies providing brokered services.
Disadvantages: 1) some services proved difficult to locate e.g., employability services, especially ones appropriate to younger teens, were scarce, and the sponsors found that they had to organize and provide these services directly. School placements were also a problem, especially for teens who refused to return to public schools but were too young to attend GED preparation, or who required special or bilingual education. Several sites consequently established tutoring programs, and one developed a pre-GED class in conjunction with the local school system. 2) A second problem with a brokerage model is that it is difficult to ensure that services provided by outside agencies are high quality and worthwhile. For the most part, staff and community women had to rely on the teens’ comments about how they were treated by the agencies to which they were referred. These comments suggest that some services were useful and engaged the teens’ interest while others were less valuable.

Sexuality and contraception: At the outset of the demonstration, the team noted that staff and community women were reluctant to confront teens on their sexual behaviour and use of birth control, and urged that program personnel adopt a more direct and forceful stance. Only 71% of the participants were scheduled for sessions in which they were specifically instructed on sexuality and contraception, and fewer than two-thirds of all participants took part in such sessions. While these figures may understate the amount of informal instruction that occurred in peer sessions and talks with community women – the relatively little attention given to this component was troubling. Indeed, repeat childbearing was characteristic of the sample as a whole: 73% of the young women had at least one additional child by the time of the 5-year interview. Contrary to the program’s objective, Project Redirection participants had more children than women in the comparison group. While the two groups had similar rates of repeat pregnancy, and were thus, presumably, equally effective (or ineffective) contractors, the experimental were less likely to terminate a pregnancy through abortion. Perhaps explained by:

- 1) ignorance - teens were aware of the availability of birth control, but the quality of their contraceptive knowledge was variable as shown ib he ethnographic study which found that many participants underestimated the chances of becoming pregnant and held numerous erroneous beliefs about the adverse health risks associated with oral contraception. For example, many thought the pill caused cancer or could cause them to have deformed babies. Or, they would stop taking the pill at the first sign of side effects but not replace it with another effective method.
- 2) perhaps a stronger and more continuous emphasis was needed by the staff and community women on imparting family planning information, and on monitoring teens’

	actual practices, but likely the quality of the family planning component only partly explains the high rates of repeat pregnancy. Teen moms expressed for their children and the value they placed on parenting classes suggest that they derived many immediate emotional rewards from parenthood. Moreover, by emphasizing the positive aspects of parenting and the development of parenting skills, the program environment may well have discouraged use of abortion.<br><br>**Retention**: On average, teens remained in the program 11.6 months -about two-thirds of the maximum time limit of 18 months. About a quarter of the enrollees left within 6 months. Overall, teens liked Project redirection. In interviews conducted for the impact analysis, 89% reported being either very or fairly satisfied with the program, and about half said that it had been helpful to them in many ways. Parenting education was considered the single most useful component; other services viewed as helpful included the community women, employability workshops, educational activities, and personal counselling.<br><br>If positive teen's attitudes toward the program, what accounts for their sporadic attendance? Problems such as illness, scheduling conflicts, inadequate public transportation, the collapse of childcare arrangements, and family and housing crises were common. But in addition, Project Redirection, in seeking to play a major role in teenagers' lives, required a good deal of participants at a time when they were adjusting to new or impending motherhood, and to a new quasi- adult status. The benefits of the program-frequent interaction with caring adults and improved access to services-could be greatest for teens with the fewest social supports, but so, too, could the burdens that participation placed on them. It appears that teens, facing numerous demands on their time and emotional energies, participated in activities, and interacted with staff and community women for as long as they found these services and relationships enjoyable and helpful, and as long as their complicated life circumstances permitted.<br><br>The program's long-term impact on educations attainment was negligible: By the time they were 22, fewer than half the former Redirection enrollees (or their comparison- group counterparts) had graduated from high school or received a GED certificate. Apparently, further education held little appeal for many participants, who had consistently experienced failure in the classroom. Intensive remedial education, perhaps offering self-paced instruction, might allow more teens to be successful learners.<br><br>Overall, most of the former Redirection participants and their children remained disadvantaged at the S-year point. Most (66%) were not working at the 5-year point, and almost half (49%) were receiving AFDC. Moreover, the mean household income, annualized, came to only $8844 for a household that, on average, contained more than four members. The cognitive skills deficits of participants' children have already been noted.
What is new? / Conclusions	At the 5-year point, when sample members were almost 22 years old on average, Project Redirection participants were more likely to be employed than members of the comparison group (34% vs. 59Y0). In addition, participants showed increased parenting skills, and their children experienced cognitive gains and exhibited fewer behavioural problems. However, at the S-year mark, fewer than half the Project Redirection participants had a high school diploma, half were receiving AFDC, and only a third were working. Equally disheartening is the fact that household income aver- aged only $737 a month, and the majority were living in poverty. While comprehensive pro- grams can help young mothers move toward self-sufficiency, further interventions are needed if more young mothers are to attain that goal, and to ensure a decent standard of living for themselves and their children.
Additional references / Other comments	Some constrains in quasi experimental study findings : 1) intention of matching between the experimental and comparison groups in terms of background characteristics, but sign difference in education attendance and important in interpreting the impact findings: nearly two-thirds of the comparison teens were enrolled in school or in an educational program at the beginning of the study, compared to fewer than half the experimental teens. 2) Initially, the plan was to evaluate

	the project's effects by comparing outcomes for program enrollees with those of young women who did not receive services, or who got only limited /fragmented ones. This was not possible probably because the nationwide increase in services for pregnant and parenting adolescents over the course of the research meant comparison- group teens received many more services than had been anticipated.<br>5-year findings: only a subsample of the women interviewed at the l- and 2-year points were included in the 5-year follow-up. Reanalysis of the earlier data for the 5-year subsample suggests that differences in outcomes over time reflect true behavioural changes, not merely changes in the individuals studied. However, the reduced size of the S-year subsample means that larger experimental-comparison differences were needed in order to attain statistical significance.

Quality Assessment – Quint 1991

Critical Appraisal	*The Mixed Methods Appraisal Tool (MMAT) 2018 will be used for quantitative, qualitative and mixed-methods studies.*<br><br>**Screening questions**<br>1. Are there clear research questions? — Yes<br>Yes, to evaluate a comprehensive programme for disadvantaged school-age mother to improve their employment, welfare, and child-related outcomes.<br><br>2. Do the collected data allow to address the research questions? — Yes<br>Data collected from multiple methods e.g. quasi experimental studies, ethnography, interview questionnaire follow ups.<br><br>**MMAT for mixed methods studies**<br>1. Is there an adequate rationale for using a mixed methods design to address the research question? — Yes<br>Yes, the rational for all method in reported in individual studies.<br><br>2. Are the different components of the study effectively integrated to answer the research question? — Yes<br>Yes, particularly the main study which incorporates findings from the different reports and studies<br><br>3. Are the outputs of the integration of qualitative and quantitative components adequately interpreted? — Yes<br>Findings reported separately, but integration from different data sources and methods occurs in multiple papers/reports.<br><br>4. Are divergences and inconsistencies between quantitative and qualitative results adequately addressed? — Yes<br>There appears not to be divergences/inconsistencies, eg qualitative data from i.e. ethnography was used to explain some of the quantitative data (i.e. contraception).<br>5. Do the different components of the study adhere to the quality criteria of each tradition of the methods involved? — CT<br>The different components are reported separately and discussed together in some of the papers/reports. It looks to adhere to quality but not much detail from some methods.

**Study ID: Waller 1999**	*Reviewers initials: CFT/MK*
**Reference:** Waller MA, Brown B, Whittle B. Mentoring as a bridge to positive outcomes for teen mothers and their children. Child and Adolescent Social Work Journal. 1999 Dec;16:467-80.	
**Other publications from same study:**	
Methods	**Study design**: other (implementing guideline/recommendation based on a current mentoring programme)<br>**Primary aim**: to compare historical and psychosocial realities of teenage parenting to prevailing contemporary myths, and provide guidelines for implementation of a community volunteer mentoring program based on an existing program in an urban community health center<br>**Intervention**: a mentoring program that connect pregnant teenagers with adults in the community who can provide them with nurturing, guidance, and hope.<br>**Baseline measurements**: age, marital status<br>**Duration of study**: Unclear, published in 1999.
Participants	**Setting**: an urban community health center, New England, US*<br>**Inclusion criteria**: Young women needing mentors volunteer for the program. The only stipulation is that they need to agree to keep appointments with their mentors. Includes girls who fall into a moderate risk category (i.e. conditions such as social isolation, childhood abuse, lack of knowledge/ experience related yo parenting, previous child protective services, multiple life stresses) and high risk (serious mental health issues, criminal histories, or active substance misuse)<br>**Total number of participants**: 45 volunteer mentors; 50 teens<br>**Mean age of participants (years) (SD):** Not reported.<br>**Mean gestational age at first visit (weeks)**: Not reported.<br>**Mean gestation age at time of intervention (weeks)**: Not reported.<br>**Ethnicity**: Not reported.<br>**Marital status**: All unmarried<br>**Socio-economic status**: Not reported.<br>**Education**: Not reported.<br>**Other lifestyle/health behaviors**:<br>** Where > 50% of the women receiving prenatal care were between 15 and 18 years of age.*
Intervention	**Type and content of intervention**:<br>Components (activities, sessions, characteristics, or behaviours):<br>- The training consists of 7 weekly two-hour sessions led by the mentor program coordinator and attended by a group of 7-10 mentor trainees. Mentors gain a knowledge and skill base in areas including prenatal care, labor and delivery, child development, child management, family violence, stress management, coping skills, interpersonal skills, problem solving skills, and accessing community resources. The training uses a multi modal approach that combines didactic in-formation, group discussion, role play, audiovisual material, and pre- sensations by health care professionals and representatives of community agencies.<br>- Fostering genuine empathy for the girls is a critical element of training. The group anticipates expectable difficulties related to establishing trust and rapport, and engages in problem-solving together. Experiential exercises and discussion of case narratives help mentors understand the subjective experiences underlying teens' overt behaviours as well as the tensions that are expectable in the mentoring relationship.<br>- The relationship between the coordinator and the prospective mentors mirrors the mentor relationship, as the program coordinator models the qualities and values required of mentors. For example, the program coordinator highlights the strengths and potential of each trainee, listens actively and empathically, validates trainees' experiences, and encourages independent problem solving.

- The 7-week time frame offers an opportunity for relationship building between the program coordinator and mentors, and this gives the program coordinator a basis for making the eventual matches between mentors and teens needing mentors. It also increases the trainees' confidence in the program coordinator, to whom they will turn for ongoing supervision and support. The group format of the mentor training has the additional benefit of establishing a network of supportive relationships among mentors.
- Curriculum:

> Session 1: the first training session focuses on defining the roles that mentors play, clarifying the parameters of each of these roles (experiencial approach)
> Session 2: strategies for establishing rapport and bolstering teens' self-esteem and characteristics of both successful and unsuccessful mentoring relationships (didactic presentation and experiential exercises)
> Session 3: overview of pregnancy, labor, and delivery (didactic and audiovisual presentations)
> Session 4: child development and parenting skills; age-appropriate and behavior-appropriate disciplinary strategies strategies.
> Session 5: family violence, with an emphasis on the dynamics of domestic abuse and child maltreatment; prevention strategies; stress management technique; resources for victims; child abuse reporting laws; limits of the mentoring role; appropriate supervisory intervention.
> Session 6: process of matching based on direct knowledge of mentors and the knowledge of individual teens provided by their caseworkers; the program coordinator determines which pairs will work well together. Mentors learn about the girls, given guidelines for first contacts, and discuss concerns (seasoned mentors invited to this session)
> Session 7: full range of local resources and learn strategies for helping teens create and sustain linkages with the resources they need e.g. linkage to concrete supports such as food, clothing, and shelter; counselling or educational resources; childcare; or expand her social support network. In preparation for this session, each mentor researches a particular community resource, presents this information to the group and provides each group member with accompanying materials. These presentations and materials provide mentors with the beginnings of a resource file to which they can refer as needed. The program coordinator supplements the information presented with additional resource materials and reminds mentors of the availability of ongoing consultation.

'Careful preparation for the initial meeting is critical to an auspicious beginning of the mentoring relationship. Both mentor and teen are provided information about one another and have input into when and how their first meeting will occur. Because the teen is often attached to the social worker and apprehensive about new relationships, it is useful for the social worker to be present at the initial meeting. In addition to facilitating the meeting, the social worker explains the differences between the roles of social worker and mentor and assures the teen that the prior relationship will continue'.

<u>Recruitment of mentors</u>: Recruitment of mentors begins with heightening community awareness of the issues confronting girls and showing members of the community how they can respond in a personal and concrete way. Recruiting can take place through speaking engagements at religious and other community organizations, newspaper articles and advertisements, local bulletins, and word-of-mouth referrals. Personal stories of teens in need of mentors (altered to protect confidentiality) are a particularly powerful means of engaging the interest of potential mentors.

Members of the community who express an interest in becoming mentors are invited to an introductory orientation. Participants learn about the needs of their community, ways the agency responds to community needs, and myths and realities related to teen pregnancy ranging from historical and demographic data to personal stories of pregnant teens in need of mentors.

The program coordinator provides a detailed description of mentor roles, such as educator, friend, coach, liaison, and broker. Presenters specify the amount of time mentors will commit. The time commitment for the mentoring program (7 weekly two-hour training sessions, monthly mentor support meetings, and two hours per week for one year in direct contact with the pregnant or parenting teen). The orientation closes with a question and discussion period.

	Following the orientation, participants who wish to continue sign up for an individual pre-training interview with the program coordinator. The pre-training interview focuses on the prospective mentor's motivations for mentoring, family experiences, views on teen pregnancy, beliefs about raising children, and remaining concerns and questions.<br><br>This interview allows both parties to evaluate the "goodness of fit" between the prospective mentor and the mentoring program.<br><br>Given the fact that teen pregnancy typically occurs in the context of multiple pre-existing psychosocial risk factors, additional criteria include interpersonal skills, cultural sensitivity, motivation, reliability, and perspective related to parenting.<br><br><u>Training and supervision of mentors</u>: The program coordinator provides ongoing supervision and support. There are <u>monthly mentor meetings</u> with continuing peer support and professional supervision, and serves as a buffer to mediate stress related to the inevitable frustrations and dif-faculties that are part of the mentoring experience. They also celebrate one another's achievements. As the mentors gain experience, they become valuable resources to one another and the program coordinator's role typically shifts from leader and teacher to group facilitator. Relationship issues are also discussed (eg set up appropriate limits, foster independence). Meetings also helpful for corrective feedback when expectations are unrealistic.<br><u>Individual supervision</u>: The program coordinator provides additional support and supervision to individual mentors on a weekly basis either by phone or face-to- face contact (plus available on a 24-hour basis in case of emergency). Mentors also receive additional support and information from the multi-disciplinary team that provides health and social services to girls.<br><br><u>Recruitment of girls</u>: Girls needing mentors volunteer for the mentoring program. The only stipulation is that girls agree to keep appointments with their mentors. Likely to benefit young women who fall into a moderate risk category: social isolation, childhood history of abuse, lack of knowledge and experience related to parenting, prior involvement with child protective services, multiple life stresses, and unwanted pregnancy. Young women who fall into the high-risk category because of serious mental health issues, criminal histories, or active substance abuse problems have needs which are beyond the scope of a community volunteer mentor program.<br><br><u>Matching</u>: Done by the program coordinator based on the relationships building during the 7-week training.<br><br><u>Duration of mentoring program</u>: Unclear.<br><br><u>Follow ups</u>: Unclear.<br><br><u>Ethics, safeguarding and support issues</u> (e.g. clean criminal records): Not reported.<br><br>**Type and content of control (if applicable):** NA.
Outcomes	**Outcome considered in the review and reported in the study:**<br><br>Authors suggest two strategies:<br>1) crucial to evaluate the effectiveness of the mentoring programme in terms (in terms of impact on pregnancy/birth outcomes, as well as abuse and neglect reports during the infants' 1 year life.<br>2) but also evaluate the effectiveness of the mentoring program in terms of the impact on the teen parents: social support (i..e. Perceived Social Support Scale by Procidano & Heller, 1983) administered before a mentor has been assigned, and again one year later.<br><br>*Consistent findings indicate that perceived support, defined as the perception that social support is or would be available if it were needed, is the best indicator for the overall construct of social support.

Underpinning theory of the intervention	The most important benefit of mentoring programs for pregnant and parenting teens is social support, a key factor in positive adaptational outcomes. Many of the biopsychosocial risk factors associated with both early pregnancy and child maltreatment may be significantly altered by the social support that mentoring relationships. For example, social support during pregnancy, including encouragement to maintain good nutrition and regular prenatal care is associated with healthy birth outcomes, and encouragement to continue with education can break the cycle of poverty. Emotional support is associated with reduced stress levels in young mothers, and education about child development and parenting skills seems to reduce the incidence of child maltreatment. Just as social isolation is a risk factor for young mothers and their children, the social support that mentoring relationships provide can lead to positive outcomes.
Challenges & strategies, lessons learn.	In the volunteer community mentoring program described here the key ingredients included: (1) community education; (2) effective strategies for recruitment, selection, and matching of mentors and teens needing mentors; (3) a training program that provides mentors with a knowledge base related to teen pregnancy and mentoring; and (4) ongoing support and supervision for mentors, including establishing linkages between mentors and multidisciplinary treatment teams.<br>The viability of a mentoring program also depends on agency investment and support, including funding for a program coordinator for a minimum of ten hours per week. While the mentoring program described here was developed and implemented at a community health center, a variation of this program might be implemented in any health care or social service setting serving pregnant and parenting teens.
What is new? (conclusions)	Punitive legislation, attitudes, and practices toward pregnant and parenting teens are costly and ineffective reactions to the problem of teen pregnancy. Community volunteer mentoring programs are one creative response to teen pregnancy that is cost effective, provides community members with an opportunity to take positive action, and can lead to improved biological, psychological, social, and economic outcomes for teen parents and their children.
Additional references / Other comments	

**Quality Assessment – Waller 1999**	*Reviewer initials: AHP/CFT*
Critical Appraisal	*Not applicable/Not feasible. The Mixed Methods Appraisal Tool (MMAT) 2018 is used for quantitative, qualitative and mixed-methods studie, but Waller 1999 is an implementing guideline/recommendation based on a current mentoring programme.*
